# Genome-wide quantification of homeolog expression ratio revealed nonstochastic gene regulation in synthetic allopolyploid *Arabidopsis*

**DOI:** 10.1093/nar/gkt1376

**Published:** 2014-01-13

**Authors:** Satoru Akama, Rie Shimizu-Inatsugi, Kentaro K. Shimizu, Jun Sese

**Affiliations:** ^1^Department of Computer Science, Tokyo Institute of Technology, 2-12-1 Ookayama, Meguro-ku, Tokyo 152-8550, Japan and ^2^Institute of Evolutionary Biology and Environmental Studies and Institute of Plant Biology, University of Zurich, Winterthurerstrasse 190, CH-8057 Zurich, Switzerland

## Abstract

Genome duplication with hybridization, or allopolyploidization, occurs commonly in plants, and is considered to be a strong force for generating new species. However, genome-wide quantification of homeolog expression ratios was technically hindered because of the high homology between homeologous gene pairs. To quantify the homeolog expression ratio using RNA-seq obtained from polyploids, a new method named HomeoRoq was developed, in which the genomic origin of sequencing reads was estimated using mismatches between the read and each parental genome. To verify this method, we first assembled the two diploid parental genomes of *Arabidopsis halleri* subsp. *gemmifera* and *Arabidopsis lyrata* subsp. *petraea* (*Arabidopsis petraea* subsp. *umbrosa*), then generated a synthetic allotetraploid, mimicking the natural allopolyploid *Arabidopsis kamchatica*. The quantified ratios corresponded well to those obtained by Pyrosequencing. We found that the ratios of homeologs before and after cold stress treatment were highly correlated (*r* = 0.870). This highlights the presence of nonstochastic polyploid gene regulation despite previous research identifying stochastic variation in expression. Moreover, our new statistical test incorporating overdispersion identified 226 homeologs (1.11% of 20 369 expressed homeologs) with significant ratio changes, many of which were related to stress responses. HomeoRoq would contribute to the study of the genes responsible for polyploid-specific environmental responses.

## INTRODUCTION

Genome duplication is a common phenomenon in Eukaryota including animals, fungi and plants. It is estimated that all flowering plant species have experienced one or more rounds of genome duplication (polyploidization) in their history (1). Moreover, 34.5% of land plants are inferred to be polyploid relative to their base chromosome number of the genus, suggesting their recent origins (2). The phenomenon is classified into autopolyploidization (derived from intraspecific genome duplication) and allopolyploidization (or genome duplication with hybridization). Allopolyploidization in particular is considered to have contributed to the adaptation to broader and novel environmental niches (3–8). Polyploid plants often live in extreme habitats, such as cold arctic regions, or dry habitats such as in the Mediterranean climate. Theoretical research performed during recent decades has revealed that the duplication of some functional genes would be advantageous (9). In addition, many of high-yield agricultural species are polyploids, which suggests that genome duplication may have played a fundamental role in the evolutionary history of speciation or adaptation to new environments (10).

To identify the molecular mechanisms underlying potential adaptation and diversification, the gene expression patterns of allopolyploids have been extensively studied (11–13). Because the genome of an allopolyploid consists of those of two (or more) closely related species, the two gene copies that originated from the parents are, in general, highly similar. These duplicated gene copies are called homeologs. Many studies of synthetic and natural allopolyploid plants showed a nonadditive and unstable gene expression pattern compared with that of the parental species, i.e. the sum of the gene expression levels of homeologs is often different from the simple sum of those of the two parents. Synthetic and natural allotetraploids of *Arabidopsis suecica* derived from *Arabidopsis thaliana* and *Arabidopsis arenosa* has been used to study the expression pattern in polyploids (13,14). In cotton species, the expression level dominance has long been discussed, with studies of these plants showing that the expression ratios of two different genomes are not even, and that gene expression could show any possible pattern (11). Such novel expression patterns are proposed to contribute to the adaptation of polyploid species (5).

However, the difficulty in distinguishing homeologs has been a major limitation of expression studies of polyploid species. Because of the high sequence similarity between two homeologs, they may be detected similarly in microarray or in polymerase chain reaction (PCR); thus, the design of homeolog-specific probes or primers for each gene is typically necessary (15,16). Moreover, reference genome sequence data for polyploid species are not readily available in most cases. Geneticists have avoided using polyploids as model species for two main reasons: first, their genome size is, by definition, larger than that of the parental diploid species and second, the genome assembly of polyploid species is extremely difficult, if not impossible (17) because genome-wide homeologous sequences hinder correct assembly.

Another obstacle to the separate detection of homeologs is the lack of large-scale observation techniques. Laborious molecular techniques such as real-time PCR or Pyrosequencing (PyroMark) can often be applied to detect homeologs even with minor differences in single nucleotide polymorphisms (SNPs). However, these techniques allow the identification of only a limited number of genes (18). Single-strand conformation polymorphism assay was applied to study the expression ratio changes of 60 duplicated pairs in natural allopolyploid and 20 pairs in synthetic allopolyploid in response to abiotic stresses (19). The use of standard microarray approaches excluding the cross-hybridization between two homeologous sequences is difficult when the number of SNPs per probe is limited. Recent studies have applied homeolog-specific microarray to cotton, but only a few hundreds of homeologs were identified (20,21).

Recent technical advances in next-generation sequencing (NGS) have allowed whole-genome transcriptomic analysis, coined RNA-seq, for a variety of species of model and nonmodel organisms. Even though NGS has been applied to the transcriptomic analysis of many diploid and polyploid species, only a few studies have undertaken the challenge of distinguishing the expression levels of homeologous genes. The most intensive analysis performed to date used cotton (22,23), in which EST data and SNPs between partial genomic sequences were used. However, the experimental verification of the quantification of the ratio of homeologs was not presented. Furthermore, the number of analyzed homeologous genes was less than half the total number of genes, which may have been due to the difficulty of computational analysis and limited parental genome information.

Similar to homeolog-specific expression, allele-specific or parent-of-origin specific gene expression analysis in diploid has been studied in medical and evolutionary research (24–33). These studies focused on model species with high-quality genomes. For example, in a study of parent-of-origin expression in F1 mice, reads were mapped on a reference that contained coding sequences of both parental strains (22,27,31,33). However, methods for allele-specific gene expression is not readily applicable to polyploid studies because high-quality reference genome of both of the parental species are usually not available. In addition, methods in allele-specific gene expression are usually designed to focus on cis-trans regulation or imprinting and thus ratio changes of homeologs in polyploid may not be analyzed.

When analyzing count data of RNA-seq reads, there is a recognized problem of overdispersion where the variance is greater than expected from a simple Poisson distribution (34–36). Overdispersion is attributed to uncontrollable differences between replicates such as in microenvironment or experimental conditions. When overdispersion is not taken into account, the number of differentially regulated genes would be overestimated. However, to our knowledge, little is known about overdipersion in the expression ratio of homologs in polyploid species.

Here we focus on the allopolyploid species *Arabidopsis kamchatica* (Fisch ex DC.) K. Shimizu & Kudoh, which is derived from Far East populations of *Arabidopsis lyrata* and *Arabidopsis halleri* (37–40). A biogeographic study of *Arabidopsis* showed that the allotetraploid *A. kamchatica* (previously known as *A. lyrata* subsp. *kamchatica*) grows in a broader climatic niche of temperature and precipitation compared with diploid *A. lyrata* and *A. halleri* (41). *Arabidopsis lyrata* tends to live in colder regions at higher latitudes, and *A. halleri* in warmer regions at lower latitudes, while the niche of *A. kamchatica* ranges from cold to warm habitats. This suggests that the allopolyploid has adapted to a broad range of environments by combining the two parental genomes. Moreover, a wealth of functional, genomic and evolutionary data are available on the model genus *Arabidopsis* including *A. thaliana*, *A. halleri* and *A. lyrata* (42–45).

In the present study, we analyzed genome-wide homeologous gene expression of allotetraploid plants using RNA-seq. We produced an artificial allotetraploid between *A. lyrata* and *A. halleri*. We developed a new bioinformatics method, termed Homeolog Ratio and quantification (HomeoRoq), to quantify the contribution of gene expression of each homeolog. In this method, we assigned RNA-seq reads to each parental genome based on the number of mismatches between the reads and the genome sequence of each parental species. Counting the number of the reads on each homeolog provided the expression level of the homeolog. The expression ratios between homeolog pairs, which were computed using this bioinformatics method, corresponded well to those measured using Pyrosequencing. We also developed a statistical method to identify genes with the change of homeolog expression ratio under different conditions. Although the ratio of homeologs before and after cold stress treatment were highly correlated, the test identified 226 homeologs with significant ratio changes, which includes many stress response genes.

## MATERIALS AND METHODS

### Artificial allotetraploid plant production

The individual of *A. halleri* subsp. *gemmifera* (Matsumura) O’Kane & Al-Shehbaz used in this article was collected from a population located in Tadaginzan, Inagawa, Osaka, Japan. *Arabidopsis halleri* is self-incompatible and harbors high levels of heterozygosity, which is not ideal for genome assembly. We therefore conducted five rounds of self-fertilization by bud pollination in the lab to reduce heterozygosity [clone of the individual halgem2 used in (39), and W302 with *S*-haplogroup A in (46)]. *Arabidopsis lyrata* subsp. *petraea* (L.) O’Kane & Al-Shehbaz was collected by Valentin Yakubov from a population at the banks of the Suharnaya river, alluvium of Kolyma, Yakutia (Sakha Republic), Far East Russia [corresponds to lyrpet4 used in (39)]. The taxon has been treated as a species or as a subspecies, including *Arabis umbrosa* Turcz. ex Steud. (1840) (47), *Arabis media* N. Busch (1922) (48) and *A. petraea* subsp. *umbrosa* Elven & D.E. Murray (2008) (49). O’Kane & Al-Shehbaz (1997) and Al-Shehbaz & O’Kane (2002) (50) considered them as a synonym of *A. lyrata* subsp. *petraea* (48,51). Shimizu-Inatsugi *et al.* (39) did not find heterozygosity in a few loci, which is consistent with high homozygosity due to auto-pollination. Following germination from a single seed, a single round of self-fertilization was conducted.

*Arabidopsis kamchatica* (Fisch. ex DC.) K. Shimizu & Kudoh is an allopolyploid that originated from *A. halleri* subsp. *gemmifera* and *A. lyrata* subsp. *petraea* in Far East Asia (37,39). Using the mutation rate given by Koch *et al.* (2006) (52), the speciation between *A. lyrata* and *A. halleri* is estimated to have occurred 2 533 980 years ago (95% CI: 1 307 952–5 166 833 years ago) (53) or 337 000 years ago (95% CI: 272 800–438 200 years ago) (54) depending on the analysis, while the origin of *A. kamchatica* by allopolyploidization is estimated to have occurred 20 417 years ago (95% CI: 0–75 460 years ago) (40). Thus, the allopolyploidization events occurred much more recently (one or two orders of magnitude more recently, in years) than the split of the parental species. It should be noted that the absolute value of the time estimation depends largely on the mutation rate, using the mutation rate given in (55), *A. kamchatica* is estimated to have originated 245 070 years ago (95% CI: 37 385–532 953 years ago) (40), although this will not affect the relative time estimate.

To obtain a synthetic hybrid, we generated an F1 hybrid by crossing *A. halleri* (female) and *A. lyrata* (male), which is in the same direction as in the four independent natural hybridization events estimated from chloroplast (plastid) sequences (39). The obtained F1 seeds were germinated and grown on sand until cotyledons developed fully, and the shoot apical meristem was treated with colchicine solution (0.5%) overnight (56). The seeds from one of these autopolyploidized F1 hybrid individuals were collected, germinated and grown for tissue sampling.

### Plant incubation

All plants were grown in a climate chamber (22°C, 60% humidity, 150 µE light intensity, 16 h light/8 h dark). For cold treatment, plants were moved into another climate chamber (4°C, 80 µE light intensity, continuous light) and incubated for 7 days.

### DNA extraction and Illumina sequencing

Genomic DNA from *A. halleri* and *A. lyrata* was extracted from leaf tissues using DNeasy plant kit (Qiagen) and the total DNA was sequenced using Illumina HiSeq 2000 with insert size of 200, 500 and 800 bp. The library synthesis was conducted at the Functional Genomics Center Zurich (http://www.fgcz.ch/) with TruSeq DNA Sample Prep Kit v2 (Illumina).

### RNA extraction and Illumina sequencing

Leaf tissues for RNA extraction were collected from the mature vegetative rosette of three individual allotetraploid plants. The RNA was extracted with TRIzol (Invitrogen) and purified further with an RNeasy Mini Kit (Qiagen). RNA concentration was measured using Qubit (Invitrogen). The RNA samples (1 µg each) were submitted to the library synthesis by TruSeq RNA Sample Prep Kit v2 (Illumina) at the Functional Genomics Center Zurich and the library was sequenced using Illumina HiSeq 2000.

### Genome assembly and gene annotation

*De novo* assemblies were performed on *A. lyrata* and *A. halleri* independently. For assembly, we used SOAPdenovo 1.05 (57) with the option ‘-d -D’. We varied K-mer’s K size in SOAPdenovo, and determined K = 73 for *A. halleri* and K = 83 for *A. lyrata* by checking N50 length, the number of predicted genes and the correspondence of the predicted genes to *A. thaliana* genes. Assembly and raw read statistics of the assembled genomes are shown in Supplementary Table S1. To predict gene regions from the genome sequences, we used AUGUSTUS 2.6 (58) with the *A. thaliana* model. Because many short genes were predicted, we selected genes whose predicted coding sequences were >200 bp.

We detected orthologous genes between the predicted genes and *A. thaliana* genes in TAIR 10 (59) using a reciprocal best hit (best-to-best) manner based on BLAST E-values of 10^−^^15^. The numbers of associated genes in H- and L-genome were 21 263 and 21 166, respectively. We adopted the annotations of *A. thaliana* orthologous genes for the gene annotation of the genes in *A. halleri* and *A. lyrata*.

### Mismatch ratio on coding regions among *A. halleri* and two subspecies of *A. lyrata*

To estimate the mismatch rate in coding sequences between our assemblies of East Asian *A. halleri*, that of East Asian *A. lyrata* subsp. *petraea* and the previously released *A. lyrata* subsp. *lyrata* sequenced by Joint Genome Institute (JGI) (45), we performed BLAST search between all gene pairs over the target species, and took the average value of the identity scores calculated. The value between *A. halleri* and our *A. lyrata* subsp. *petraea* is 2.44%, from which we expected that most of Illumina paired-end reads could be mapped to both genomes and have an average of 4.93 mutations per paired-end read (202 bp length) used to identify genomic origin of the reads. Additionally, a considerable mismatch rate (0.85%) between our *A. lyrata* subsp. *petraea* from East Asia and JGI’s *A. lyrata* subsp. *lyrata* was found, which suggests divergence at the subspecies or species level (see ‘Discussion’ section).

### Homeolog identification

To identify homeologs, we associated *A. halleri* genes with *A. lyrata* genes using BLAST with an E-value threshold of 10^−^^15^ and regarded the associations whose conserved regions are >200 bp as homeologs. We identified 31 749 homeologs. Results from this method were not completely symmetrical: when we selected *A. lyrata* genes as the query and *A. halleri* as the database to perform BLAST, the number of homeologs identified becomes 31 560. The reason is that single H-homeologs may be associated with multiple L-homeologs caused from the division of single L-homeolog gene into two or more scaffolds, and vice versa. As the scaffold N50 of the H-genome is longer than that of L-genome, and thus H-genome is more reliable than L-genome, we selected *A. halleri* as a standard in all analyses.

### Evaluation of HomeoRoq with simulated RNA-seq reads

To assess the measurement accuracy of our strategy, we generated simulated RNA-seq reads and estimated their genomic origin. Even though each gene should exhibit variable expression in actual tissues, we generated the simulated reads on the assumption that all genes had the same expression level. We also supposed that the total expression levels of H-homeologs were equal to those of L-homeologs. The average insert length of the simulated reads was 200 bp, and we introduced random mutation at 0.1% rate, mimicking the RNA-seq error rate by Illumina.

We computed the accuracy of mapped reads by checking the correctness of the gene where the reads are mapped. For the H-origin read, we checked whether the mapped position is in the gene from which the read was generated. For the L-origin read, we checked whether the L-homeolog from which the read is generated is a homeolog of the H-homeolog on which the read is mapped. The accuracies of H-origin and L-origin reads were 99.9 and 89.4%, and their average was 94.6% (Supplementary Table S2).

To count homeologs whose expression ratios could be quantified by HomeoRoq, we measured the percentage of simulated reads generated from H-homeolog identified as H-origin on each H-homeolog. We assume that when the percentage of the identifiable reads is >50% of the reads from a homeolog, it indicates that HomeoRoq can quantify the homeolog-specific expression from the gene.

### Mapping of RNA-seq reads

RNA-seq reads were obtained from synthetic *A. kamchatica* in two conditions (control and cold stress) with three replicates. Supplementary Table S3 shows the statistics from the RNA-seq experiments. The reads were mapped onto the L- and H-genome independently. Although the mapping software TopHat (60) is used frequently for RNA-seq, its upper limit of the number of mismatches is small and allows the mapping of only <50% of reads. We therefore used STAR-2.2.0 for mapping. We ran STAR without any special option and it allowed the maximum 10 mismatches for the alignment because the alignment accuracy was high even under these parameters (Supplementary Table S2). If a read was mapped on multiple positions, the aligned position with a ‘primary’ flag (position with the best alignment score) was adopted to quantify expression levels to avoid ambiguous result caused from multiple hits.

### Pyrosequencing

Homeologous gene expression ratios were verified using the Pyrosequencing of five genes. This technique has been used for allele frequency detection (18), DNA methylation analysis in combination with bisulfite conversion (61) and others during the past decade. Pyrosequencing can provide relative abundance of SNPs by comparing the intensity of light emission generated by the incorporation of allele-specific bases during the sequencing reaction by polymerase. We applied this technique to detect the frequency of homeologous cDNA that originated from the H- and L-genomes.

The set of gene-specific (PCR amplification) primers and sequencing primer(s) was designed by PyroMark Assay Design v2.0 software (Qiagen). The gene-specific amplification primers were designed at conserved regions between H- and L-homeologs, so that they include SNP positions inside the amplified region. The sequencing primer was designed at adjacent conserved region to a targeted SNP position(s) between H- and L-homeologs. Two sequencing primers were designed for each set of amplification primers, and one or more SNPs were designated as the target per sequencing primer (Supplementary Table S4).

The RNA samples that were used for Illumina RNA sequencing were converted into cDNA using the High Capacity RNA-to-cDNA kit (Invitrogen). DNA fragments were amplified with amplification primers by ExTaq polymerase (Takara). The amplified DNA fragments were sequenced using PyroMark ID system (Qiagen) with the sequencing primers at the Genetic Diversity Center, Zurich. The obtained peak was automatically analyzed using the PyroMark system using AQ (allele quantification) mode to determine the SNP ratio. The multiple SNP ratios obtained by multiple sequencing primers for each gene fragment were averaged to decide the H- and L-homeolog ratios.

### Differential expression between H- and L-homeologs

To identify H- and L-homeologs whose expressions changed statistically significantly under different conditions, the RNA-seq reads sorted either as H- or L-homeolog using HomeoRoq were subjected to DESeq (34). We set False Discovery Rate (FDR) threshold to 0.05.

### Statistical method for the detection of ratio changes incorporating overdispersion

To detect the changes in the ratio of homeolog expression in response to the stress condition, we developed a method to check whether the ratio of H-homeolog to L-homeolog expression was changed between the conditions. In this method, we calculated the probability that the homeolog expression ratios between the two conditions are identical from given RNA-seq data. Then, when the probability was less than significance level, the homeolog could be regarded as having different expression ratio under the conditions.

Let *R_i_* be a homeolog expression ratio in condition *i*. Let *h_ij_* and *l_ij_* be the numbers of H- and L-origin reads on sample *j* in condition *i*. The probability that the ratios between the two conditions C and O are equal can be described as P(*R*_C_ = *R*_O_ | *h_ij_*, *l_ij_*). As we could not compute this probability directly, we transformed it by using Bayes theorem:

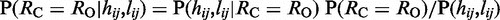



Suppose that the two conditions are observed independently, it can be described as follows:

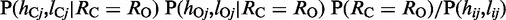



As we had only three samples from each condition, it was difficult to directly calculate P(*h_Cj_*, *l_Cj_* | *R*_C_ = *R*_O_) and P(*h_Oj_*, *l_Oj_* | *R*_C_ = *R*_O_). To estimate the probability, we used an expression-level model incorporated in the RNA-seq read analysis (34,35). In the RNA-seq model, expression level obeyed a normal distribution, whose mean was average expression level of each gene, and variance depended on the mean expression level of each gene and a shot noise, which was uniquely determined on each condition and each gene. In homeolog expression, in addition to the two variables, the variance depended on expression ratio of each homeolog pair. We therefore assumed that the variance of homeolog expression levels was described as *f*(*m_i_*, *R_i_*) + *d_i_*, where *m_i_* was the mean expression level of condition *i*, and *d_i_* was the shot noise. *f* was computed by using a local regression method (we used the locfit package in R). Among the three parameters, if *R_i_* was fixed, mean expression *m_i_* was calculated from the total expression *h_ij_* + *l_ij_* multiplied by *R_i_*. From the two values, regression value *f*(*m_i_*, *R_i_*) was calculated. The shot noise was calculated as the residue from the real variance to *f*(*m_i_*, *R_i_*).

In real calculation, *R_i_* was unknown. We therefore used a truncated normal distribution ranged from 0 to 1 as the prior distribution on P(*R_C_* = *R_O_*). Although beta distribution is frequently used as the distribution of ratios, this distribution was likely not to fit the distribution of ratios especially when the ratios between the two conditions were not close, resulting in false-negative detection of ratio changes. We therefore used the truncated normal distribution where the mean and variance of the distribution were computed from real data *h_ij_* and *l_ij_*_._.

Once we fixed *r*, the probability P(*r*)* = *P(*R_C_* = *R_O_*) was found from the distribution. Also P(*h_Cj_*, *l_Cj_* | r) was calculated from the normal distribution whose mean is *m_i_* and variance of *f*(*m_i_*, *r*) + *d_i_*. Similarly, P(*h_Oj_*, *l_Oj_* | r) was calculated, and hence P(*h*_C_*_j_*, *l*_C_*_j_* | *R*_C_ = *R*_O_ = r) P(*h*_O_*_j_*, *l*_O_*_j_* | *R*_C_ = *R*_O_ = r) was calculated. By selecting *r* from a truncated normal distribution whose mean and variance are calculated from *R_i_*, P(*h*_C_*_j_*, *l*_C_*_j_* | *r*) P(*h*_O_*_j_*, *l*_O_*_j_* | *r*) P(*r*) can be calculated. Because P(*h_ij_*, *l_ij_*) is independent from the sampling, calculation of P(*h*_C_*_j_*, *l*_C_*_j_* | *r*) P(*h*_O_*_j_*, *l*_O_*_j_* | *r*) P(*r*) one million times with random samplings and comparison of them with the value calculated from observed values gave the value of P(*R*_C_ = *R*_O_ | *h_ij_*, *l_ij_*) of a homeolog. We computed the *P*-value for each homeolog. In total, we observed 31 749 raw *P*-values, and then we performed Benjamini-Hochberg (BH) correction to them to compute corrected *P*-values for multiple tests.

### Gene ontology analysis

Gene Ontology (GO) analyses were performed with the GOTermFinder Web site (62) on 27 February 2013. We first associated homeologs with *A. thaliana* TAIR gene ID, and then used GO terms related to the TAIR gene ID. In all, 20 814 genes were associated with at least one GO term. Among 226 homeologs with significant ratio changes, 153 of these homeologs were associated with at least one GO term.

## RESULTS

### Generating synthetic allotetraploids

**Figure 1.**
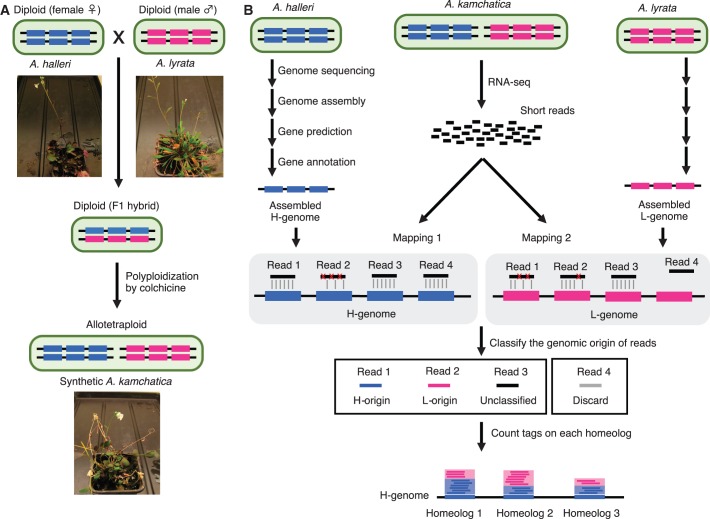
Overview of the quantification of the expression level ratio of homeologs. (**A**) The generation of synthetic allopolyploid mimicking *A. kamchatica*. HomeoRoq was applied to this species. (**B**) Homeolog discriminative RNA-Seq pipeline. Genome/gene sequences of parental species are individually assembled. RNA-seq reads observed from the target allopolyploid are mapped onto both H- and L-genomes independently. Based on the number of mismatches, genomic origin of the reads is classified.

In the present study, we generated synthetic allotetraploid plants from two parental taxa of *A. kamchatica*, i.e. *A. halleri* subsp. *gemmifera* and *A. lyrata* subsp. *petraea* (sensu O’Kane & Al-Shehbaz, 1997, which is also called *A. petraea* subsp. *umbrosa*) (48,49,51), both collected in Far East Asia (Figure 1A). The genome size of each taxa was ca. 220 Mb measured by flow cytometry (CyFlow® Space, Partec) with 4′,6-diamidino-2-phenylindole (DAPI) (CyStain UV precise P, Partec). The F1 hybrid individual of these two species was treated with the classic polyploid inducer colchicine, and allotetraploid seeds were obtained from the treated individual. The three individuals that germinated from these seeds were used for RNA-seq. The genome size of this synthetic allotetraploid was ca. 450 Mb, which corresponds to the sum of those of the two parents.

We denote the genomes of *A. halleri* and *A. lyrata* as H-genome and L-genome, respectively, the origin from *A. halleri* and from *A. lyrata* as H-origin and L-origin, respectively, and *halleri*-derived and *lyrata*-derived homeolog as H-homeolog and L-homeolog, respectively.

### Method for quantifying the ratio of the expression of homeologs

We developed a new algorithm HomeoRoq to detect changes in the ratios of the expression of homeologs under different conditions using RNA-seq data obtained from the synthetic allopolyploid species. Our analysis was composed of two parts: the first part aimed to quantify homeolog-specific expression level (Figure 1B), and the second part aimed to test statistically the ratio of change in the expression of each homeologous gene pair under different conditions.

During the first step of expression quantification, we assembled the genome of two parental taxa of the allopolyploid *A. kamchatica*, i.e. *A. halleri* subsp. *gemmifera* and *A. lyrata* subsp. *petraea* (Figure 1B, Table 1 and Supplementary Table S1). Because our previous study (39) had shown that *A. lyrata* subsp. *lyrata* from North America, whose genome has been sequenced and assembled by JGI (45), was not directly involved in the origin of *A. kamchatica*, we sequenced *A. lyrata* subsp. *petraea* from Far East Asia. Moreover, we aimed to demonstrate that HomeoRoq works without the assembled genome reported previously. We sequenced the genomic DNA of both parents of the synthetic polyploid using Illumina sequencing technology. The total sequence amounts were 36.5 Gb (166-fold coverage of the estimated genome size) for *A. halleri* and 67.8 Gb (308-fold coverage of the estimated genome size) for *A. lyrata*. The total genome lengths were 202.97 and 221.14 Mb, respectively, which were consistent with the genome sizes estimated using flow cytometer. The scaffold N50 sizes were 17 686 and 7848 bp, respectively.

**Table 1.** Statistics of parental species’ genes

Parental species	*A. halleri*	*A. lyrata*
Number of predicted genes	36 737	35 392
Number of genes related to *A. thaliana*	21 263 (57.9%)	21 166 (59.8%)
Number of homeologs	31 749 (86.4% based on *A. halleri*)	
Number of expressed homeologs	18 928 (59.6%)	19 186 (60.4%)

Between *A. halleri* and *A. lyrata*, similar number of genes was found and expressed. The relationships between predicted genes and genes in *A. thaliana* were computed using reciprocal best-hit strategy based on BLAST alignment score. The expressed homeologs are counted from the six RNA samples, and we regarded a gene/homeolog expressed when its RPKM is >0.1.

Next, we predicted the gene regions of each species with *A. thaliana* model parameters and identified 36 737 genes for *A. halleri* and 35 392 genes for *A. lyrata* (Table 1). Homeologous gene pairs between them were identified using a BLAST search (63) from all *A. halleri* genes to all *A. lyrata* genes. Based on the BLAST results with an E-value threshold of 10^−^^15^, a one-way hit strategy associated *A. halleri* genes with *A. lyrata* genes. This procedure yielded 31 749 homeologous gene pairs (Table 1).

Last, we identified the origin of RNA-seq reads obtained from synthetic *A. kamchatica*. Here, we define the genomic origin as whether the read was derived from the H- or L-genome. Each read was mapped separately onto each of the H- and L-genomes and the number of mismatches between the read and each genome was counted. The genomic origin was decided according to the lower number of mismatches (read 1 and read 2, in Figure 1B). In the case of a perfect match of the read to both genomes, or the presence of an identical number of mismatches, the reads were considered ‘unclassified’ (read 3, in Figure 1B) because these conditions did not allow the genomic origin of the read to be determined. If the read was mapped to only one of the parental genomes (read 4, in Figure 1B), we did not use it, as using this type of read to quantify homeolog expression levels may cause a bias in the estimation of expression levels attributed to the difference in quality of the assembled genomes of two parental species, even though part of them would truly represent species-specific fragments. We counted the numbers of the H- and L-origin reads on each gene on H-genome and took the ratio of the reads on each gene, which is regarded as the expression level ratio of the homeologs.

To assess whether HomeoRoq can be used to estimate the genomic origin of the RNA-seq reads observed for the allopolyploid accurately, we calculated the classification accuracies from simulated RNA-seq reads generated from the L- and H-genomes. We generated 10 million artificial reads from the genomes, added the random sequence error and estimated the genomic origin of reads using HomeoRoq. When the sequencing error rate was set to 0.1%, 72.2% of the reads were classified as having either an H- or L-origin; in addition, 94.6% of the classified reads were used to quantify correct homeologs (see ‘Materials and Methods’ section). Even if the error rate was varied, it had little effect on the accuracies of read classification (Supplementary Table S2). Moreover, our method quantified homeolog-specific expression from 27 874 homeologs (75.9% of the total genes, 87.8% of the homeologs) as far as they are expressed (see ‘Materials and Methods’ section for detail). These simulations validated our algorithm as an effective method for identifying the genomic origin of RNA-seq reads.

### RNA sequencing and homeolog-specific expression

RNA samples were extracted from leaf tissues of three synthetic allotetraploid individuals grown at 22°C (referred to as the control condition). These individuals were further incubated at 4°C for 7 days, and leaf tissues were sampled again (referred to as the stress condition) to extract RNA. In total, six RNA samples (three individuals in two conditions) were sequenced using Illumina (Table 2).

**Table 2.** Statistics of mapped results (total of three samples)

	Control (three samples)		Stress (three samples)	
Sample condition	Number of reads	Rate in all reads	Number of reads	Rate in all reads
Number of all reads	119 123 927		102 602 463	
Mapped on H-genome	110 262 849	92.60%	95 775 209	93.40%
Mapped on L-genome	110 369 840	92.70%	95 026 179	92.60%
Mapped on both genomes	104 910 672	88.10%	90 702 938	88.40%
Discarded reads (Read 4 in Figure 1)	14 213 255	11.90%	11 899 525	11.60%

	Rate among reads “Mapped on both genomes’		Rate among reads “Mapped on both genomes’	

H-origin reads (Read 1)	48 278 507	46.00%	40 828 935	45.00%
L-origin reads (Read 2)	45 059 363	43.00%	39 073 036	43.10%
Unclassified reads (Read 3)	11 572 802	11.00%	10 800 967	11.90%

**Figure 2.**
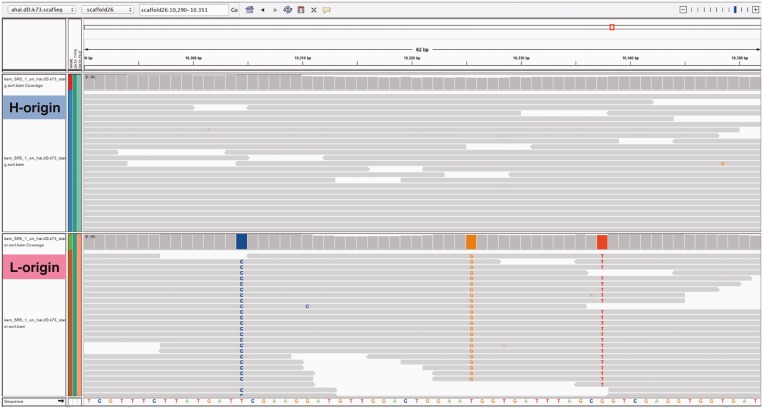
A visualization example of mapped reads on H-genome. H-origin reads are perfectly matched to the H-genome, while L-origin reads have a few mismatches to the H-genome. This confirms that the proposed method estimate genomic origin successfully.

We estimated the genomic origin of each RNA-seq read by mapping them onto H- and L-genomes as describe above. In three control samples, 92.6 and 92.7% of the reads were mapped onto the H- and L-genome, respectively, and 88.1% of them were mapped onto both genomes, which was used to measure homeolog expression. Of the reads that were mapped onto both genomes, 46.0, 43.0 and 11.0% were categorized as H-origin, L-origin and unclassified, respectively (Table 2). The visualization of the mapping results confirmed that our method was able to distinguish the genomic origin of the RNA-seq reads (Figure 2). Similar results were obtained from the samples with cold stress treatment (Table 2).

The mapping results showed that 18 928 (59.6%) and 19 186 (60.4%) genes were expressed [Reads Per Kilobase per Million mapped reads (RPKM) > 0.1] from H-homeologs and L-homeologs, respectively, and 17 745 genes were expressed from both homeologs (Supplementary Table S3). These statistics confirmed that both homeologs of the majority of genes were expressed under these conditions.

### Experimental validation using Pyrosequencing

**Figure 3.**
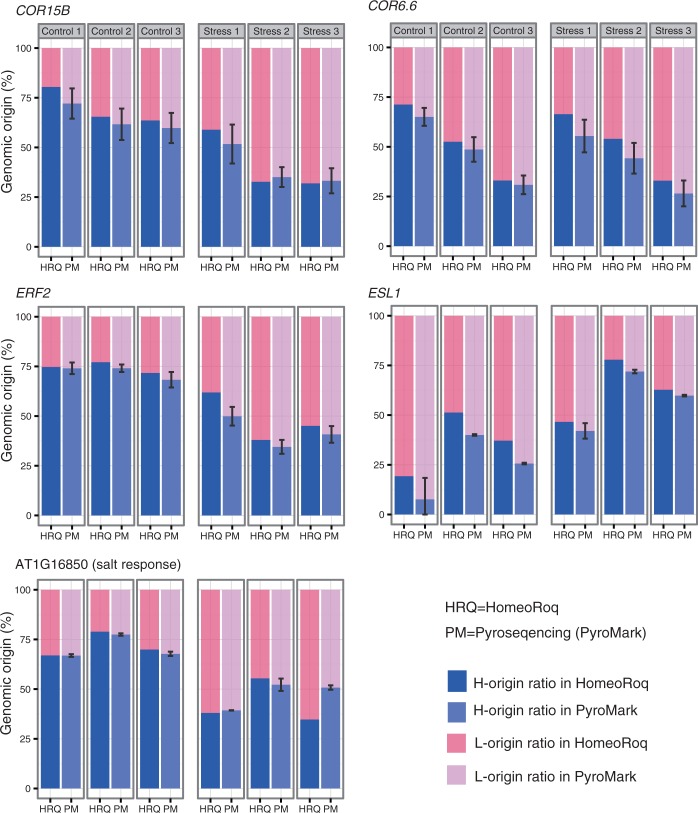
Comparison of homeolog expression ratios between the bioinformatics analysis and Pyrosequencing. The homeolog expression ratios of five genes estimated by HomeoRoq and Pyrosequencing (PyroMark) are compared. The data are shown for three individuals at control (ctrl) and stress conditions.

To verify homeolog expression quantified using RNA-seq reads, we conducted a Pyrosequencing analysis using PyroMark for five genes known to be involved in stress response [*COLD REGULATED 6.6 (COR6.6), COR15B*, *ETHYLENE RESPONSIVE FACTOR-2 (ERF2)*, *EARLY RESPONSE TO DEHYDRATION SIX-LIKE 1 (ESL1)* and AT1G16850] (Supplementary Figure S1). We amplified by PCR the target-gene fragments from the cDNAs that had been reverse-transcribed from the same RNA samples used for the Illumina RNA-seq and subsequent HomeoRoq analysis. We measured the homeolog ratio at multiple SNP positions (see details in Supplementary Table S4) and averaged the ratio of H-homeolog (Figure 3). In most of the samples, the results of Pyrosequencing were consistent with those of the HomeoRoq result. In particular, the RNA-seq results obtained for the *COR15B* gene were mostly inside the error bar of the Pyrosequencing results. Even when the RNA-seq quantifications of a few individuals lay outside the error-bar range of the Pyrosequencing results, as was the case for *COR6.6* or *ESL1*, the overall tendency of homeolog expression ratio for whole individuals was consistent with that obtained using RNA-seq quantification. A known technical limitation of Pyrosequencing is that PCR amplification may be biased to a particular homeolog. Considering the potential technical bias of the Pyrosequencing method (18), our estimation of homeolog ratios using HomeoRoq with Illumina RNA-seq data works reasonably well.

### Expression ratio change of homeologous pairs

Cold treatment is known to affect the expression level of a large number of plant genes (64,65). To find differentially expressed H- and L-homeologs, the sequence reads sorted by HomeoRoq were subjected to standard RNA-seq analysis software DESeq (FDR < 0.05). We found that the expression of 2897 and 4364 (9.12 and 13.7%) of H- and L-homeologs, respectively, were changed significantly by cold stress (Supplementary Figure S2A and B). These results encouraged us to proceed to examine the changes in homeolog expression ratio.

**Figure 4.**
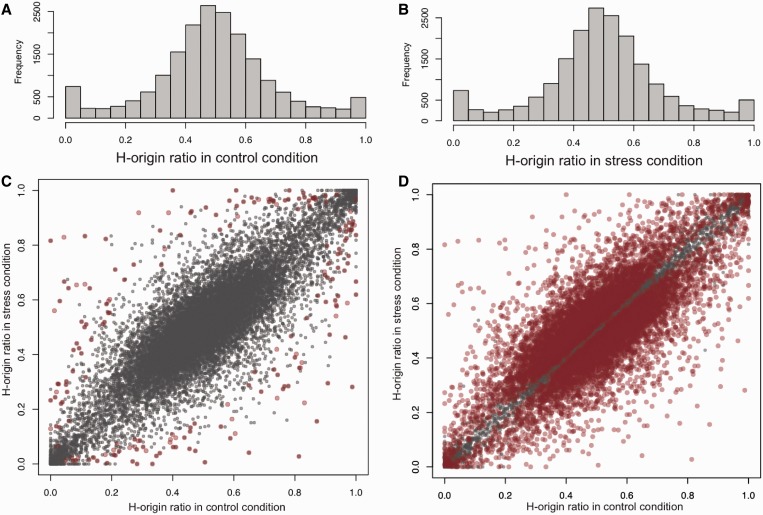
The ratio of homeolog expression level. (**A**) Histogram of expression ratio in control condition. (**B**) Histogram of expression ratio in stress condition. (**C**) Scatterplot of the ratio of homeolog expression level between two conditions. Each dot corresponds to one homeologous pair. Red dots indicate homeologs whose expression ratio was significantly changed over the conditions. (**D**) Scatterplot of the ratio of homeolog expression levels. Red dots indicate homeolog whose expression ratio was regarded as significantly changed over conditions with Fisher’s exact test.

Using the homeolog-specific RNA-seq data sorted by HomeoRoq at control and stress conditions, we checked the distribution of the homeolog expression ratio for each condition (Figure 4A and B). The overall histogram shapes of the homeolog expression ratio in both control- and stress-condition results were fairly symmetric, which confirmed that both homeologs were expressed in the two conditions. The distributions created the impression that the ratio may be determined randomly, as both distributions were similar to a normal distribution.

The ratios of homeologs, however, were highly correlated between the two conditions (Figure 4C. Pearson’s *r* = 0.870), suggesting that most homeologous pairs maintain their expression ratio even after cold stress treatment. Moreover, Figure 4C showed the existence of specific homeologs with expression ratios that were greatly changed by cold treatment.

Fisher’s exact test, the chi-squared test and the binomial test are typically used to detect expression ratio differences (22). However, by checking the variance of homeolog expression levels, we confirmed that the distribution was overdispersed when compared with a binomial test (Supplementary Figure S3). In the presence of overdispersion, highly expressed homeologs tend to be deemed significant even if the actual change is small. The use of Fisher’s exact test to identify homeolog expression changes led to the identification of 11 442 homeologs (36.0%) that were deemed significant (adjusted *P* < 0.05 with BH multiple test correction). Figure 4D (red points) indicates that homeologs with a ratio that was not greatly changed were regarded as significant in Fisher’s exact test. This value appears too large compared with the numbers of differentially expressed H- and L-homeologs extracted by DESeq analysis (9.12 and 13.7%), and thus Fisher’s exact test does not seem to be an appropriate method.

To overcome this problem, we developed a statistical test that can handle overdispersion. This method computes the appearance rate of the observed counts under the assumption that the two conditions have identical ratios (see ‘Materials and Methods’ section for detail). This test led to the estimation of 226 homeologs (1.11% of 20 369 expressed homeologs) as being significant (adjusted *P* < 0.05 with BH correction) (Figure 4C, red points). Supplementary Table S5 includes the list of the homeologs.

These 226 genes with significant change in homeolog ratio were subjected to Gene Ontology analysis. Twenty-three categories showed enrichment (adjusted *P* < 0.01 with Bonferroni correction), most of which include similar sets of genes related to stress response (see ‘Discussion’ section; Supplementary Table S6). In Supplementary Figure S4, the expression changes of 49 genes in the category of response to stress were depicted with an arrow.

## DISCUSSION

### A novel method to quantify the expression level ratio of homeologs

The large-scale approaches available currently to measure homeolog-specific expression require deep knowledge about the genome/gene sequences of both parental species because they use known SNP positions to measure homeolog expression. Yoo *et al.* (22) used RNA-seq and counted the number of reads on SNPs. They could measure less than half of the genes and used only 17.1% of RNA-seq reads to measure the ratio of homeolog expression, suggesting that as low as 0.085% of nucleotides (0.171 of 200 bp) could be used for the estimation of genomic origin of a read. The very low rate would come from the difficulty to identify high-quality SNPs from allopolyploid species even if the parental species have similar genome sequences. By contrast, our newly developed method dramatically increased the number of reads with an estimated genomic origin, and used 78.1% of the reads. Computer simulation showed that the ratio of 87.8% of the homeologs can be measured when they are expressed. This increase in usable reads is key for a highly quantitative genome-wide result. While the method introduced by Page *et al.* (23) could classify the genomic origins of half the reads, the method requires high-quality and high-density SNP calling in advance of the classification. It is too restrictive to analyze allopolyploids because the genomes of most have not been sequenced yet or, even if the genome was sequenced, the quality is generally not high because of the high similarity between homeologous genomes. Our method does not require the SNP detection procedure, and the genomic origins of the reads are estimated by the comparison of the mapping status between the parental species. We also verified that our quantified ratio corresponded well to Pyrosequencing results, which was not the case in the analyses performed by other methods (22,23).

We would like to note that mapping of RNA-seq reads independently to both genomes is critically important. A simpler workflow would be to construct hypothetical polyploid genome by concatenating the two parental genomes, and then to map RNA-seq reads to the concatenated reference. This strategy is frequently used by the methods to detect allele-specific or parent-of-origin expression, and would work for model species with high-quality genome (25,27). However, the estimation of the expression level ratio between homeologs as well as expression level itself would be biased, due to the reads that could be mapped to only one of the parental genome (corresponding to the read 4 in Figure 1B) unless high-quality genome of both parents would be available.

One of the advantages of HomeoRoq is that it works with draft genomes or gene sequences assembled using a RNA-seq assembler (66). Plant genomes tend to have many repetitive elements and to be large size, which would prevent the generation of high-quality genome sequences. Even if the genome assemble quality is not high, HomeoRoq only uses highly homologous regions between the parental species’ genomes. In addition, HomeoRoq does not need prior SNP analysis between parental species, which were used in the existing methods to measure homeolog-specific expression as well as allele-specific expression.

### Draft genome assembly of *A. halleri* and *A. lyrata* from Far East Asia

*Arabidopsis halleri* is one of the species closest to the model plant *A. thaliana,* and stable transgenic technique using *Agrobacterium* is established (67). Because of its heavy metal tolerance, the species has been a model for phytoremediation including transcriptomic studies (67–69). Moreover, a wide range of evolutionary and ecological studies has been conducted, including self-incompatibility (40,46,70,71), predictive model of flowering gene expression *in natura* (72,73), speciation and population structure (74–76), and defense against herbivores (77,78). Phylogeographic and population studies showed that European and Asian accessions share nuclear haplotypes (39), suggesting an individual of either region would be valuable for genome sequencing of *A. halleri*. The genome assembly has not been available so far, possibly because of high level of heterozygosity due to outcrossing, and to its relatively higher genome size among diploid *Arabidopsis* species. Here we conducted four rounds of self-fertilization of *A. halleri* subsp. *gemmifera*, and generated a draft genome assembly (N50: 18 kb). Although it was beyond the scope of this work to produce a high-quality genome assembly, the presented assembly contains at least 90% of genes estimated from the statistics of RNA-seq (Table 2) and may provide useful genetic sequences for future functional, transcriptomic and evolutionary studies.

*Arabisopsis lyrata* is also a model for evolutionary and ecological studies (79), including mating systems (80,81) and soil adaptation (82). While the genome sequence of American strain MN47 of *A. lyrata* subsp. *lyrata* was reported (45), our genome sequence of *A. lyrata* subsp. *petraea* from Far East Russia turned out to be diverged from the reported genome (45) with at least 0.85% divergence in coding regions. This fact highlights the high diversity of the *A. lyrata* species complex. Plants from Far East Russia were recognized as a distinct taxon named *Arabis umbrosa* (47), *Arabis media* (48) or *Arabidopsis petraea* subsp. *umbrosa* (49), and has been treated as a species or as a subspecies. O’Kane & Al-Shehbaz (1997, 2002) considered it as a synonym of *A. lyrata* subsp. *petraea* (50,51). The genome sequence, however, suggests that a new taxonomic combination *A. lyrata* subsp. *umbrosa* would be appropriate, which should be verified in future studies.

### Expression level ratio after stress treatment: stable in most genes and changes in 1.11% when overdispersion is taken into account

Expression studies of polyploid have focused on stochasticity of gene regulation, based on the data that gene expression levels are highly variable between individuals (12,13). Our genome-wide data showed for the first time that homeolog expression levels were overdispersed. Supplementary Figure S3 shows that the distribution of total amount of expression levels of homeolog pairs was overdispersed, and that overdispersion was observed even if we focused on the expression of homeologs from a single origin.

Our study showed that majority of the ratio of homeolog expression was stable in response to the treatment (*r* = 0.870) (Figure 4). If expressed homeologs were randomly selected, the ratio would obey a 2D normal distribution with no correlation between two conditions. The high correlation of the ratios indicates that biological elements such as binding site sequences of transcription factors or epigenetic status could be shared between homeologs. This highlights nonstochastic aspects of the gene expression pattern in polyploids. This suggests that the majority of homeologous pairs are regulated similarly in stress response, and the cellular network system in the allopolyploid has robustness against the stress.

We also observed the genes with drastic changes in the ratio in response to cold stress (Figure 4). To identify such genes, Fisher’s exact test has been applied previously in both homeolog and allele-specific expression analysis (22,27,31,33). However, the overdipersion of gene expression levels would cause overestimation of the number of genes with ratio changes. Figure 4D illustrates the genes with significant change in homeolog ratio if Fisher’s exact test is used, and implies that Fisher’s exact test would be too liberal to identify the ratio changes. The Fisher’s exact test regarded as high as 36.0% of homeolog pairs as significant ratio changes. This is odd because only 9.12 and 13.7% of H- and L-homeologs, respectively, were differentially regulated according to DESeq (34) (FDR < 0.05; Supplementary Figure S2), and the number of genes with ratio change should be even less owing to the high correlation of the ratio of homeologs between the two conditions (Figure 4C). We speculate that the difference of the numbers should be caused from the no consideration of the overdispersion to detect significant changes in Fisher’s exact test, while DESeq can handle the overdispersion.

We therefore developed a new statistical test to find genes with the ratio change in homeolog expression level in two conditions. So far, the main interest of allele-specificity studies has been the balance between allele-specific expression ratio at a certain condition. On the other hand, our purpose is to detect expression regulation changes under different conditions. With this method, we identified 226 genes (1.11%) with significant changes in the ratio of homeolog expression level. Those outlier genes might play an important role in the environmental adaptation of polyploid species, and thus the analysis of such environment-specific homeolog preference might lead to discover new functional modules under the specific environment.

**Figure 5.**
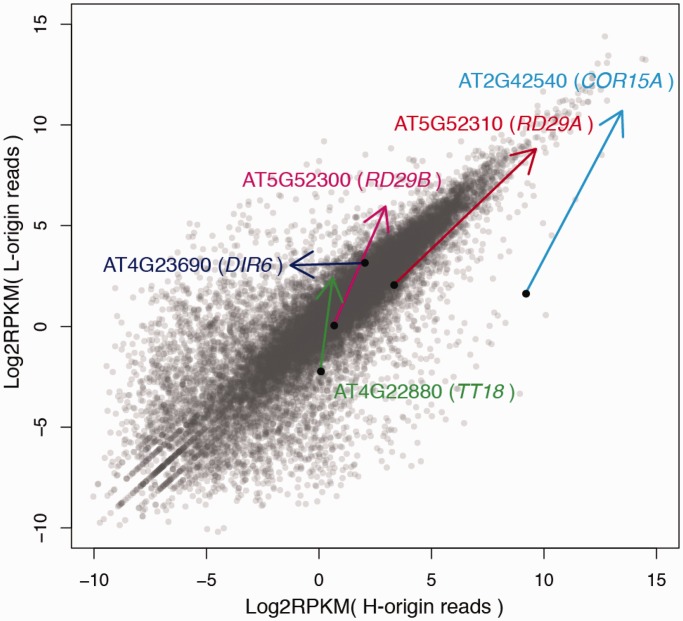
Changes of homeolog-specific expression levels. X-axis and Y-axis are RPKM of H- and L-origin expressions, respectively. Gray dots represent expression levels of the entire homeologs in control environment. Each arrow from a black point means the expression change by the cold stress. The tip of the arrow indicates the expression level of the gene after the cold stress.

Gene Ontology analysis of the 226 genes revealed 23 GO terms (*P* < 0.01 after multiple test correction). Most of these 23 categories appear to be related to abiotic stress, including jasmonic acid and hormone-related processes, reactive oxygen-related processes and secondary metabolism. Cold stress is known to induce stress hormones such as abscisic acid and jasmonate (83,84), which further activate the phenylpropanoid pathway to produce anthocyanin and other flavonoids with antioxidative function (85). Not surprisingly, most of the listed GO categories are related to the homeostasis of plants under stress conditions. Supplementary Figure S5 shows the expression changes of 49 genes in the category of responses to stress (GO: 0006950). The category includes *TT18*/*ANS (TRANSPARENT TESTA 18*/*ANTHOCYANICIN SYNTHASE)* (83) and *DIR6 (DIRIGENT PROTEIN 6)* (86) encoding enzymes in flavonoid and lignan pathways of polyphenol synthesis, respectively. L-homeolog of *TT18* was highly upregulated by the cold stress treatment, but little changes were observed in H-homeolog. In contrast, H-homeolog of *DIR6* was downregulated, suggesting balance change in phenylpropanoid biosynthetic process (Figure 5).

Well-studied markers of cold acclimation include the tandemly duplicated genes *RD29A/LTI78 (RESPONSIVE TO DESSICATION 29A/LOW TEMPERATURE INDUCED 78)* and *RD29B/LTI65,* encoding hydrophilic soluble proteins with possible cryoprotective function (87,88). Interestingly, the L-homeolog was upregulated more than H-homeologs in *RD29B* (Figure 5), while both homeologs of *RD29A* were similarly upregulated. It suggests a different control of these two tandemly duplicated genes. Another well-known marker gene of cold response is *COR15A* (*COLD-REGULATED 15A*), encoding a protein localized in soluble fraction of chloroplast stomata with possible cryoprotective function (88,89). The L-homeolog showed elevated upregulation relative to the H-homeolog.

Differential regulation of these pairs of homeologs strongly suggests that the gene networks in stress responses diverged between *A. lyrata* and *A. halleri* during a few million years since their speciation (53). Considering that *A. lyrata* tends to live in a colder environment than *A. halleri* (41), it is possible that the higher induction of cold acclimation genes like *COR15A* or *RD29B* may contribute to its ability to cope with greater cold stress. Alternatively, the change in expression in the two diploid species may be similar, and allopolyploidization might have resulted in the creation of a novel property in the gene network or in stochastic expression patterns. These alternative hypotheses can be tested by studying the expression changes of natural polyploid individuals as well as of the diploid parents.

### Future application and development of the new method

The next challenge would be to distinguish the homeologs in the natural allotetraploid *A. kamchatica*. The genome sequence of natural polyploids of this species shares high similarity with, but is not identical to, its diploid ancestors because sequence evolution occurred since polyploidization. Moreover, we cannot obtain the actual diploid parental plant individuals, but only their closest living relatives. Recent advances in NGS technologies now produce longer reads more easily, and mapping software is able to handle an increased number of polymorphisms efficiently. By integrating these techniques, the present method can be modified to analyze natural allopolyploids. Moreover, our methods will be applicable not only to RNA-seq but also to genome resequencing. Because at least four independent polyploidization events have been estimated for *A. kamchatica* (39), this species would be a suitable model to address the question of repeatability and stochasticity in the expression changes induced by allopolyploidization. Furthermore, our new bioinformatics methods should be applicable to any allopolyploid species. The introduced statistical technique is also applicable to the analysis of allele-specific expression changes. All scripts are available from http://seselab.org/homeoroq/.

## ACCESSION NUMBERS

The raw data have been submitted to DDBJ Short Read Archive, and are available through accession numbers DRP001138 (*Arabidopsis lyrata* subsp*. gemmifera* DNA-seq), DRP001139 (*Arabidopsis halleri* subsp*. petraea* genome) and DRP001140 (*Arabidopsis kamchatica* RNA-seqs). The genome sequences have been submitted to DDBJ, and are available through accession numbers PRJDB1392 (*Arabidpsis halleri* subsp*. gemmifera*) and PRJDB1393 (*Arabidopsis lyrata* subsp*. petraea*).

## SUPPLEMENTARY DATA

Supplementary Data are available at NAR Online.

## FUNDING

Young Investigator Award of Human Frontier Science Program (to K.K.S. and J.S.); Marie Heim-Vögtlin grant of the Swiss National Science Foundation (SNF) and Forschungskredit of University of Zurich (to R.S.I.); Swiss National Science Foundation (SNF) (to K.K.S.); the University Research Priority Programs of Evolution in Action of the University of Zurich and of Systems Biology/Functional Genomics (to R.S.I. and K.K.S.); JSPS KAKENHI [23128504, 24651227 and 24680032 to J.S.] Grant-in-Aid for Scientific Research on Innovative Area "Genome Science", Ministry of Education, Culture, Sports, Science and Technology in Japan (to J.S.). Funding for open access charge: Human Frontier Science Program.

*Conflict of interest statement*. None declared.

## Supplementary Material

Supplementary Data
