# Supplementary material for: Genome-wide quantification of homeolog expression ratio revealed nonstochastic gene regulation in synthetic allopolyploid Arabidopsis
Source: Nucleic Acids Res. 2014 Jan 13;42(6):e46. doi: 10.1093/nar/gkt1376 (PMC3973336; doi:10.1093/nar/gkt1376)
Supplement: Supplementary Data [file supp_42_6_e46__index.html]

Genome-wide quantification of homeolog expression ratio revealed nonstochastic gene regulation in synthetic allopolyploid Arabidopsis — Genome-wide quantification of homeolog expression ratio revealed nonstochastic gene regulation in synthetic allopolyploid Arabidopsis — Supplementary Data 

# Genome-wide quantification of homeolog expression ratio revealed nonstochastic gene regulation in synthetic allopolyploid *Arabidopsis*

## Supplementary Data

files

**Files in this Data Supplement:**

- Supplementary Data - pdf file
- Supplementary Data - doc file
- Supplementary Data - xlsx file
- Supplementary Data - doc file
- Supplementary Data - xlsx file
- Supplementary Data - xlsx file
- Supplementary Data - xlsx file
- Supplementary Data - docx file
