# Supplementary material for: Genome-wide quantification of homeolog expression ratio revealed nonstochastic gene regulation in synthetic allopolyploid Arabidopsis
Source: Nucleic Acids Res. 2014 Jan 13;42(6):e46. doi: 10.1093/nar/gkt1376 (PMC3973336; doi:10.1093/nar/gkt1376)
Supplement: Supplementary Data [file supp_gkt1376_nar-01585-met-k-2013-File014.pdf]

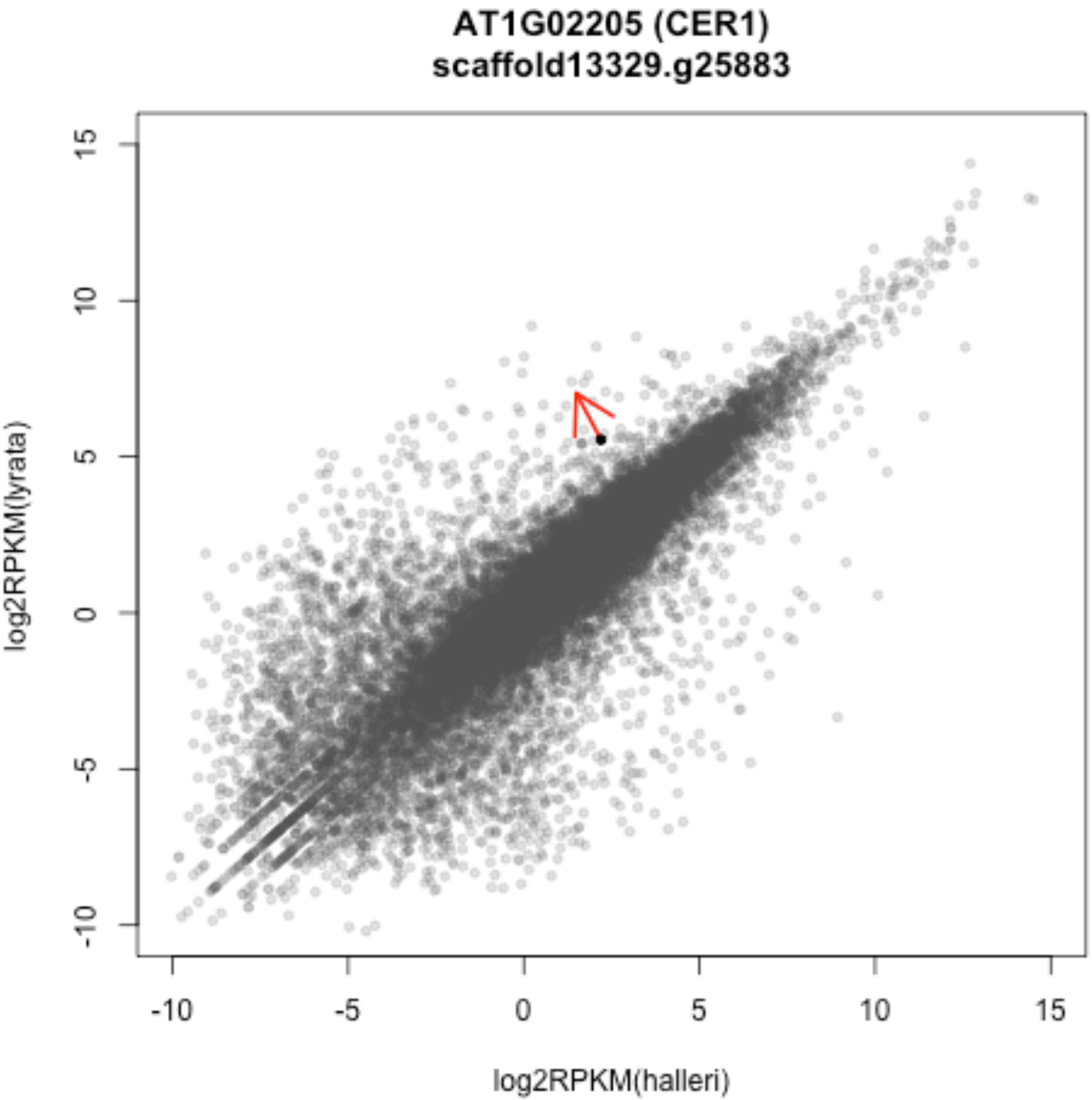
AT1G02205 (CER1)
scaffold13329.g25883
log2RPKM(lyrata)
log2RPKM(halleri)
15
10
5
0
-5
-10
-10
-5
0
5
10
15

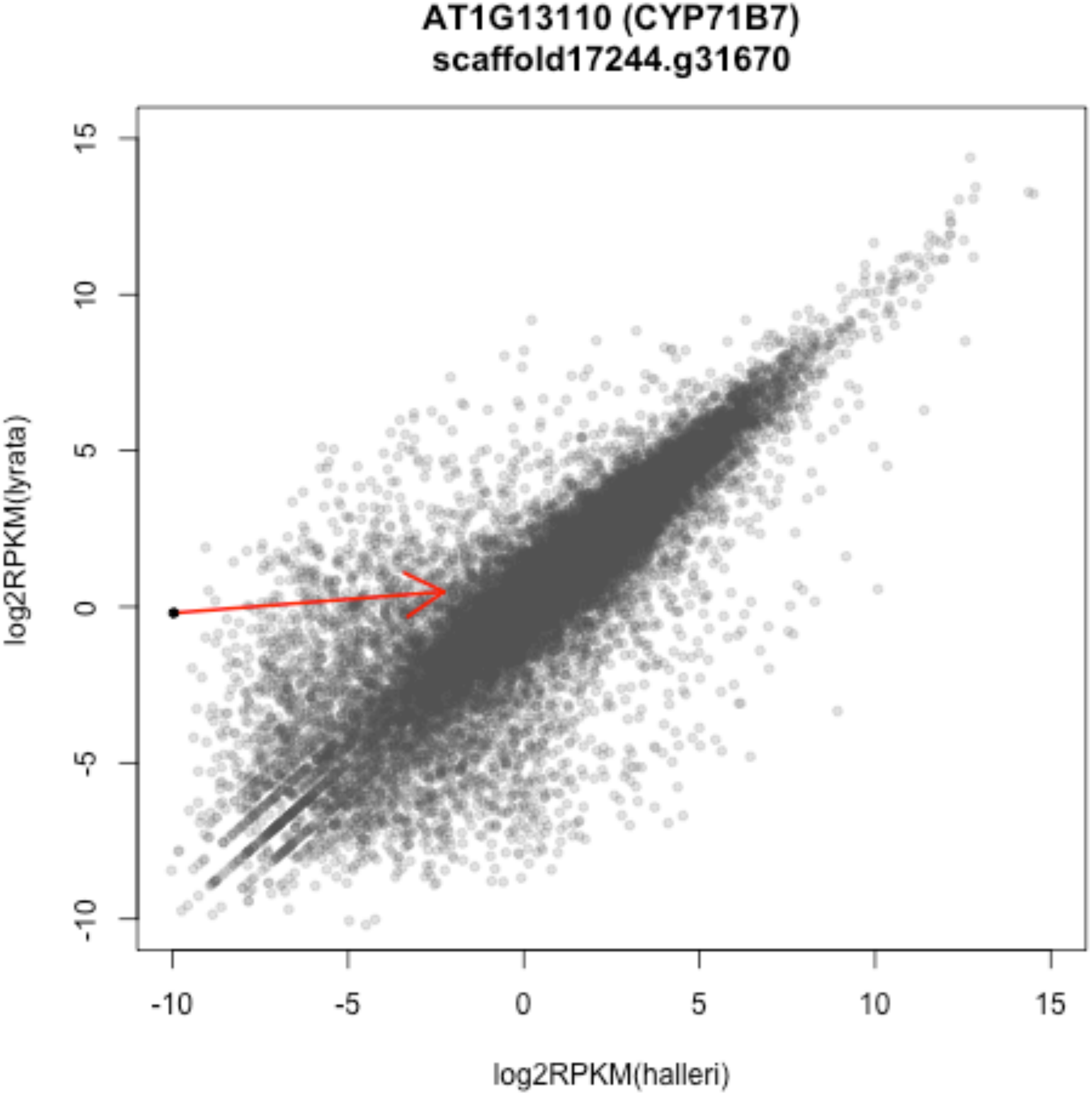

AT1G13110 (CYP71B7)
scaffold17244.g31670
log2RPKM(lyrata)
log2RPKM(halleri)
15
10
5
0
-5
-10
-10
-5
0
5
10
15

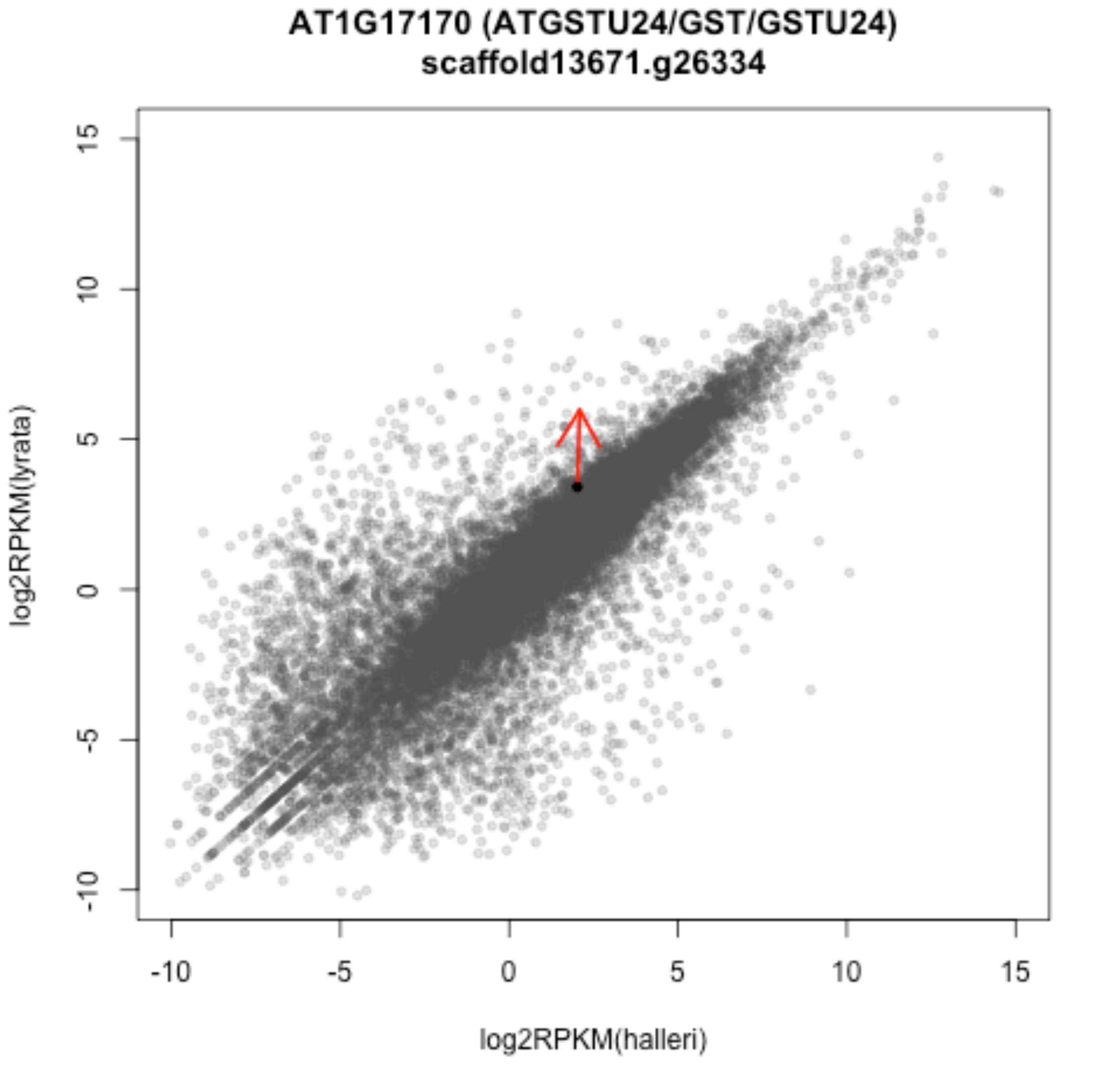
AT1G17170 (ATGSTU24/GST/GSTU24)
scaffold13671.g26334
log2RPKM(lyrata)
log2RPKM(halleri)
-10
-5
0
5
10
15

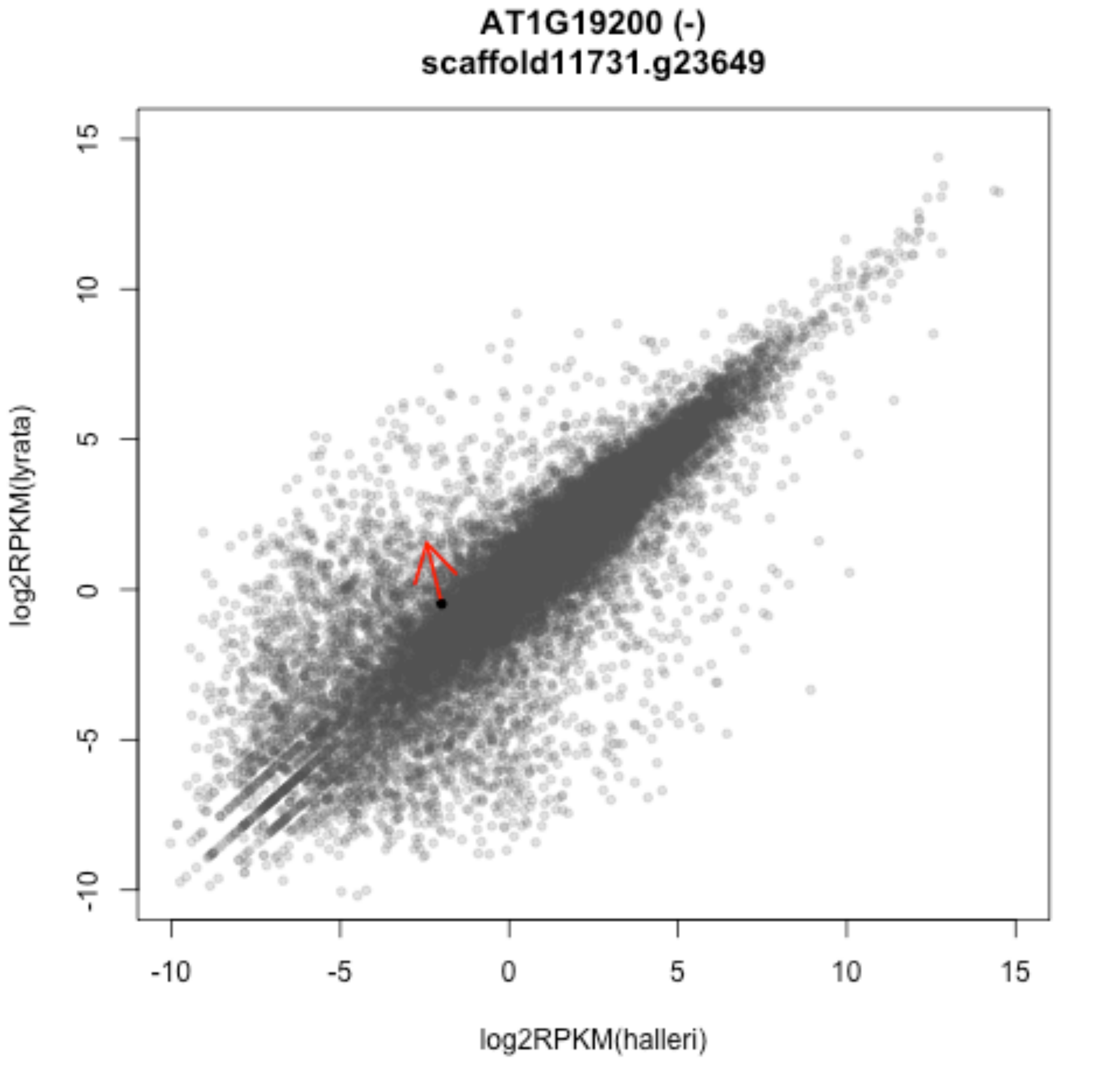
AT1G19200 (-)
scaffold11731.g23649
log2RPKM(lyrata)
log2RPKM(halleri)
-10
-5
0
5
10
15

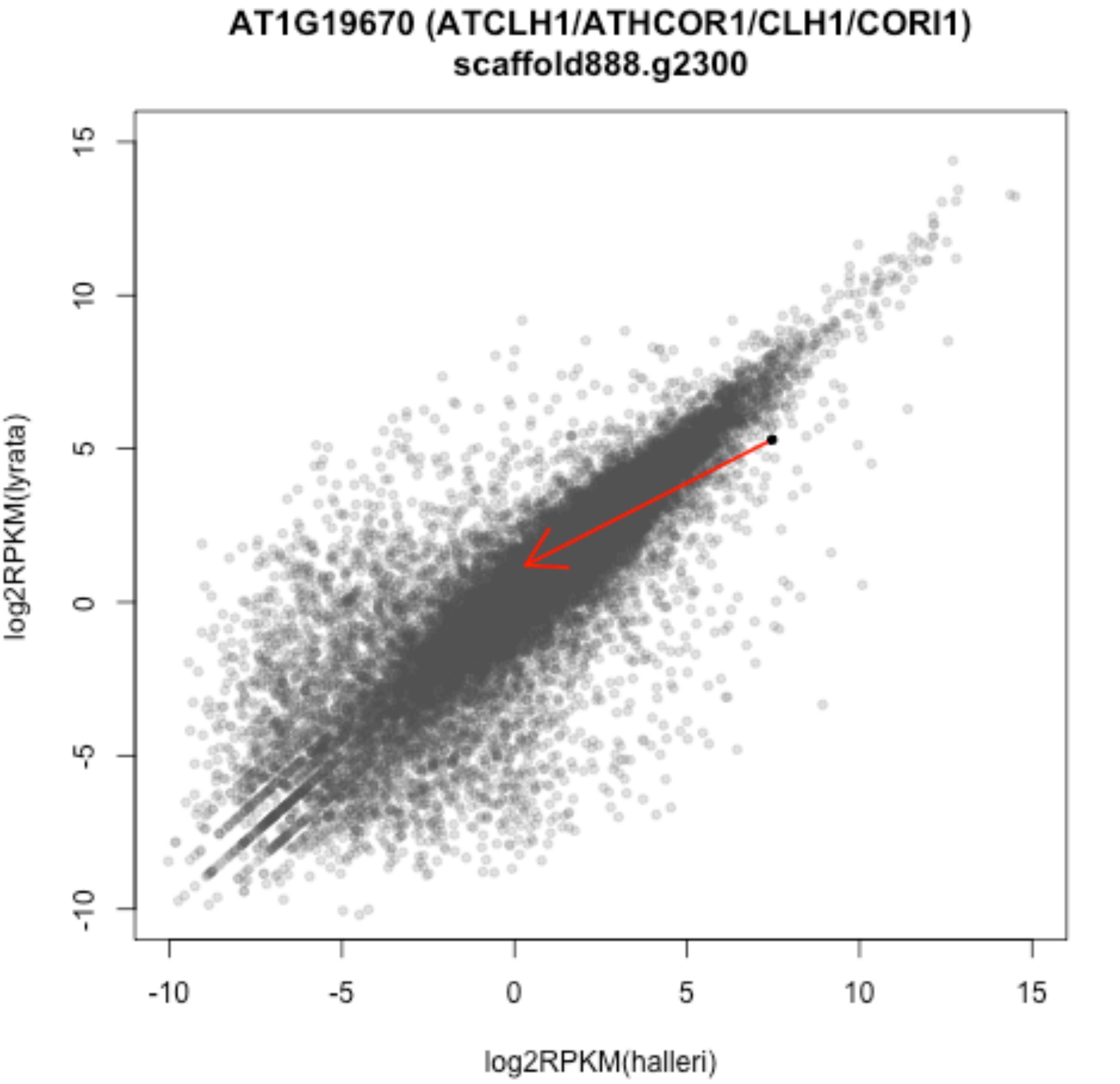
AT1G19670 (ATCLH1/ATHCOR1/CLH1/CORI1)
scaffold888.g2300
log2RPKM(lyrata)
log2RPKM(halleri)
-10
-5
0
5
10
15

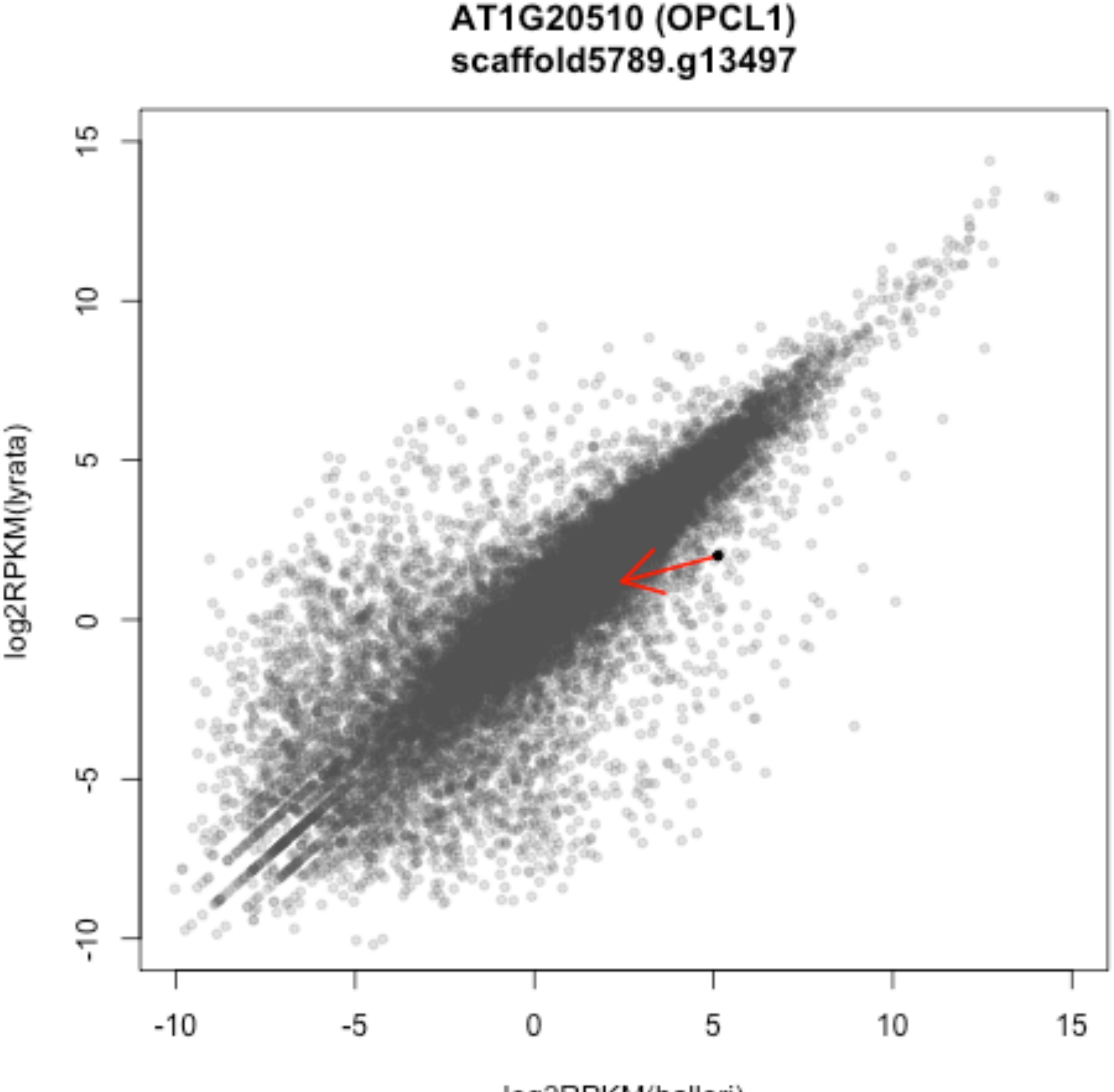
AT1G20510 (OPCL1)
scaffold5789.g13497
log2RPKM(lyrata)
log2RPKM(halleri)
15
10
5
0
-5
-10
-10
-5
0
5
10
15

AT1G20870 (-)
scaffold6629.g15118

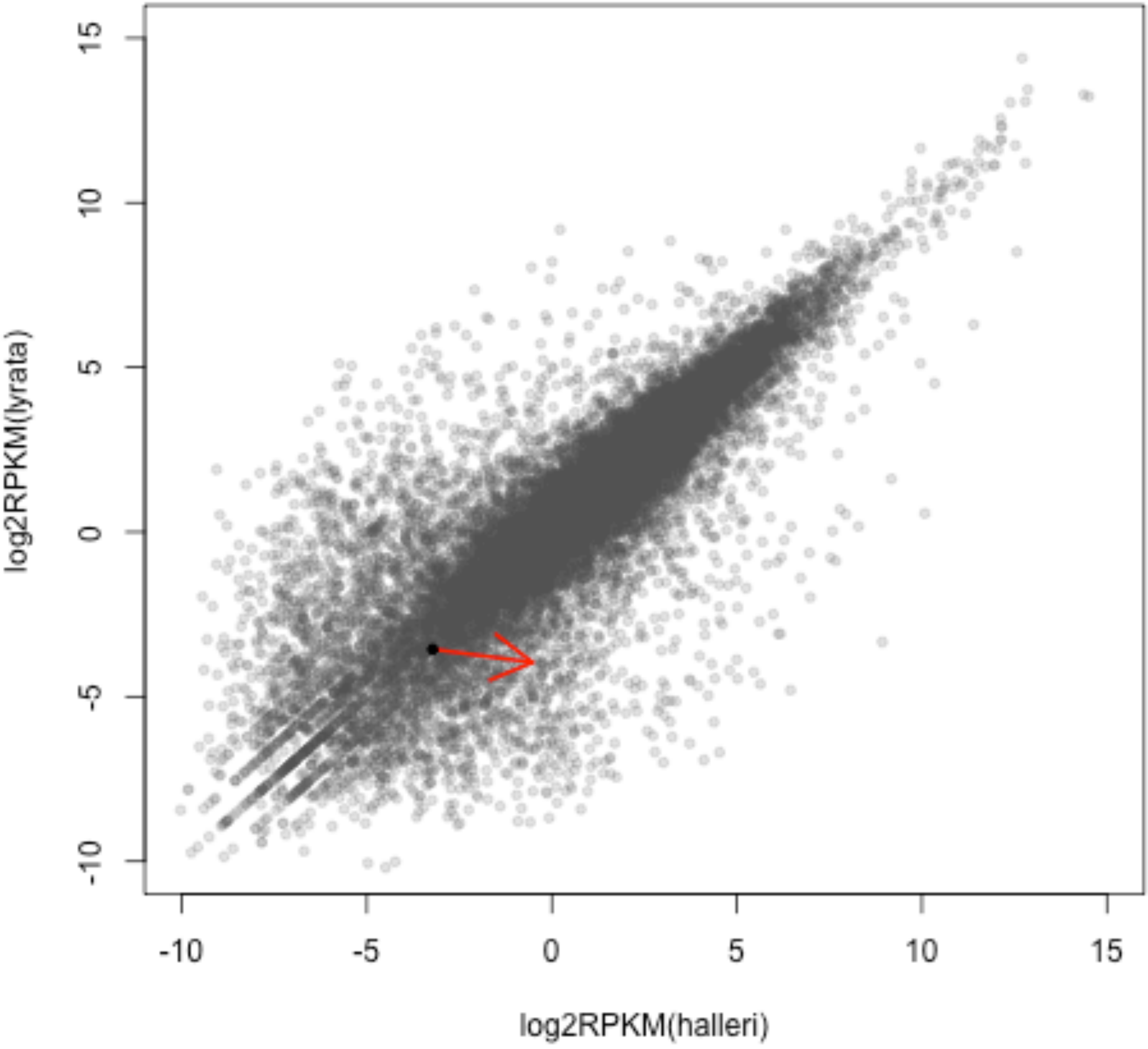

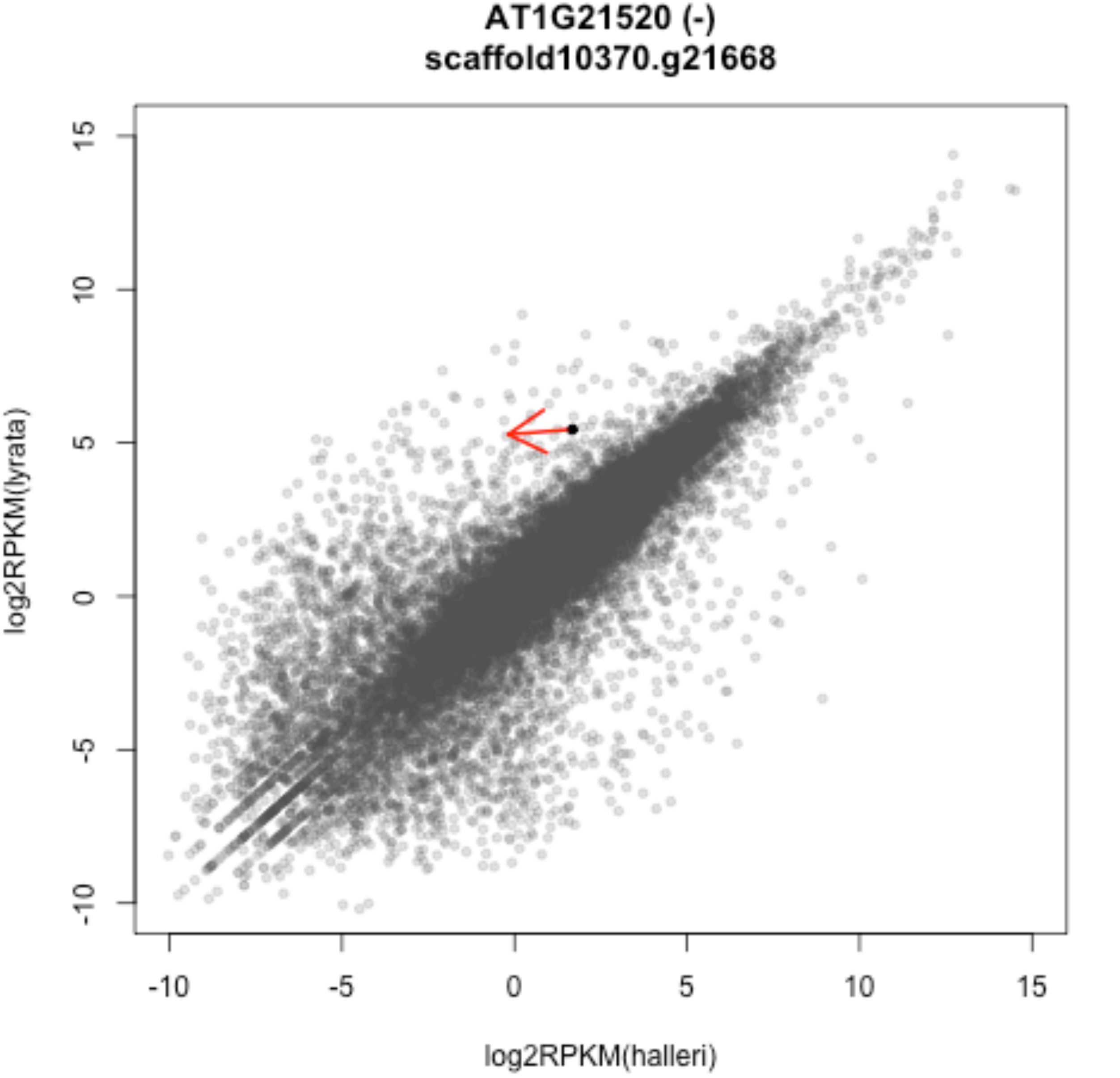
AT1G21520 (-)
scaffold10370.g21668
log2RPKM(lyrata)
log2RPKM(halleri)
15
10
5
0
-5
-10
-10
-5
0
5
10
15

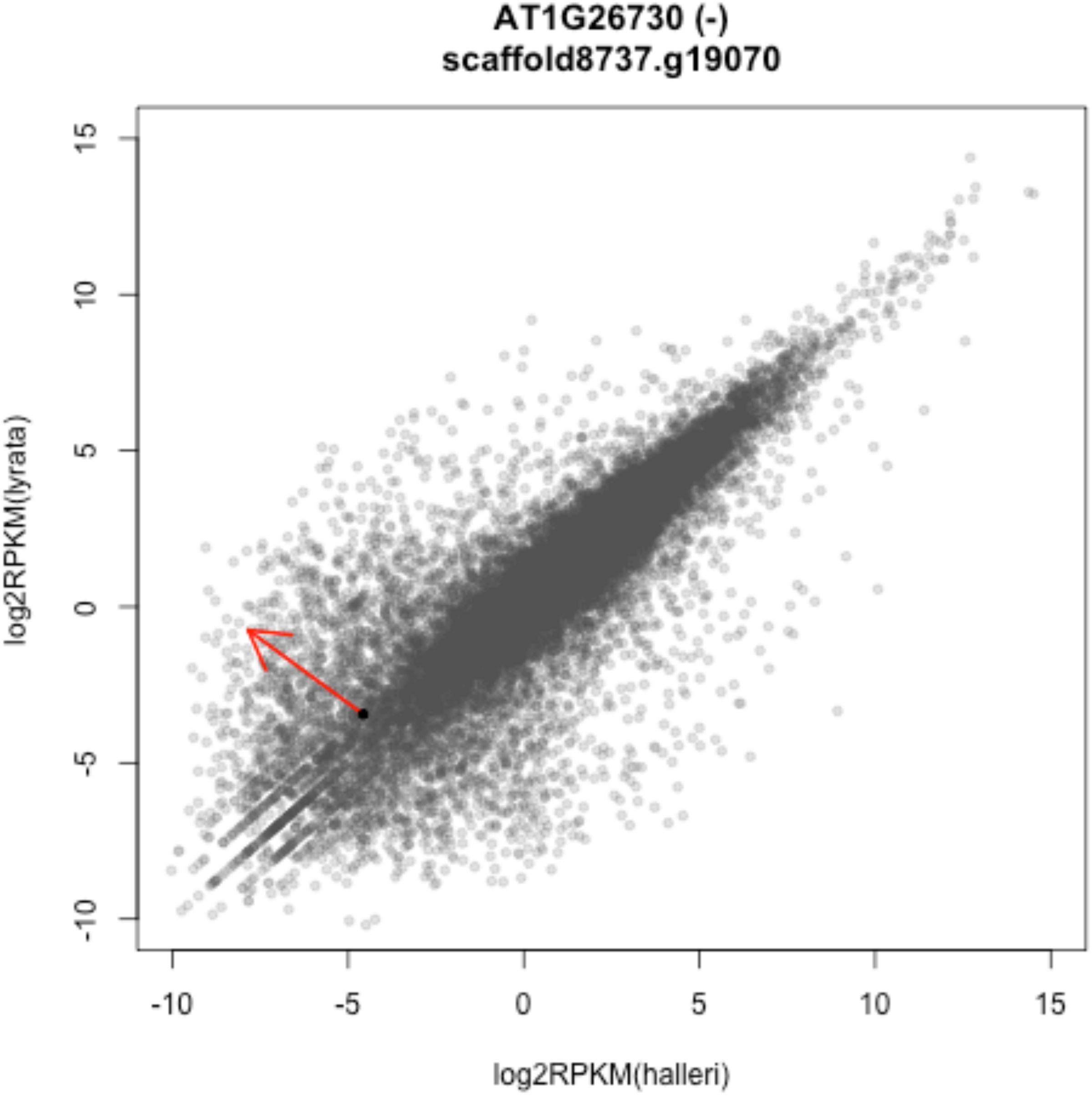
AT1G26730 (-)
scaffold8737.g19070
log2RPKM(lyrata)
log2RPKM(halleri)
-10
-5
0
5
10
15

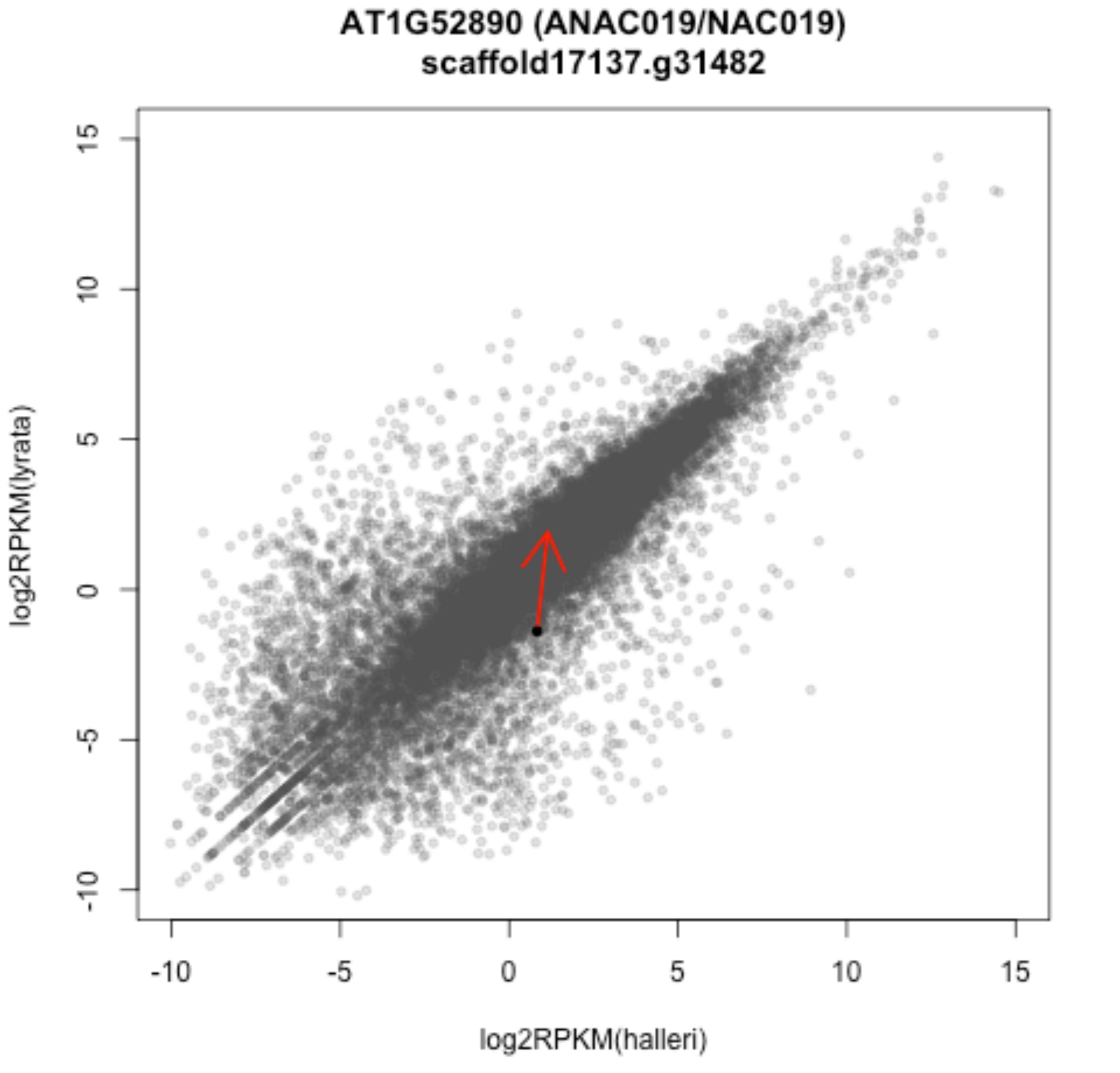
AT1G52890 (ANAC019/NAC019)
scaffold17137.g31482
log2RPKM(lyrata)
log2RPKM(halleri)
15
10
5
0
-5
-10
-10
-5
0
5
10
15

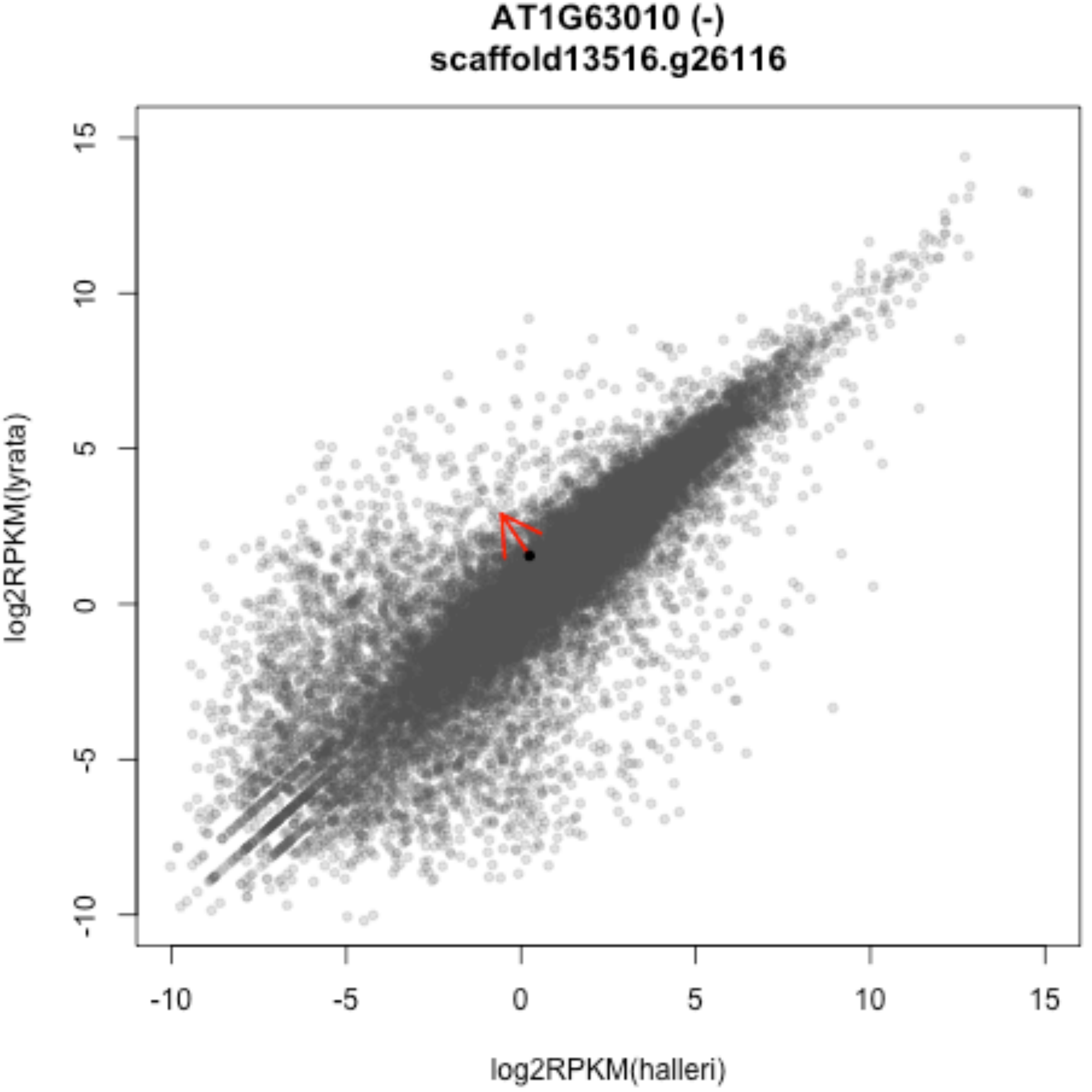
AT1G63010 (-)
scaffold13516.g26116
log2RPKM(lyrata)
log2RPKM(halleri)
-10
-5
0
5
10
15

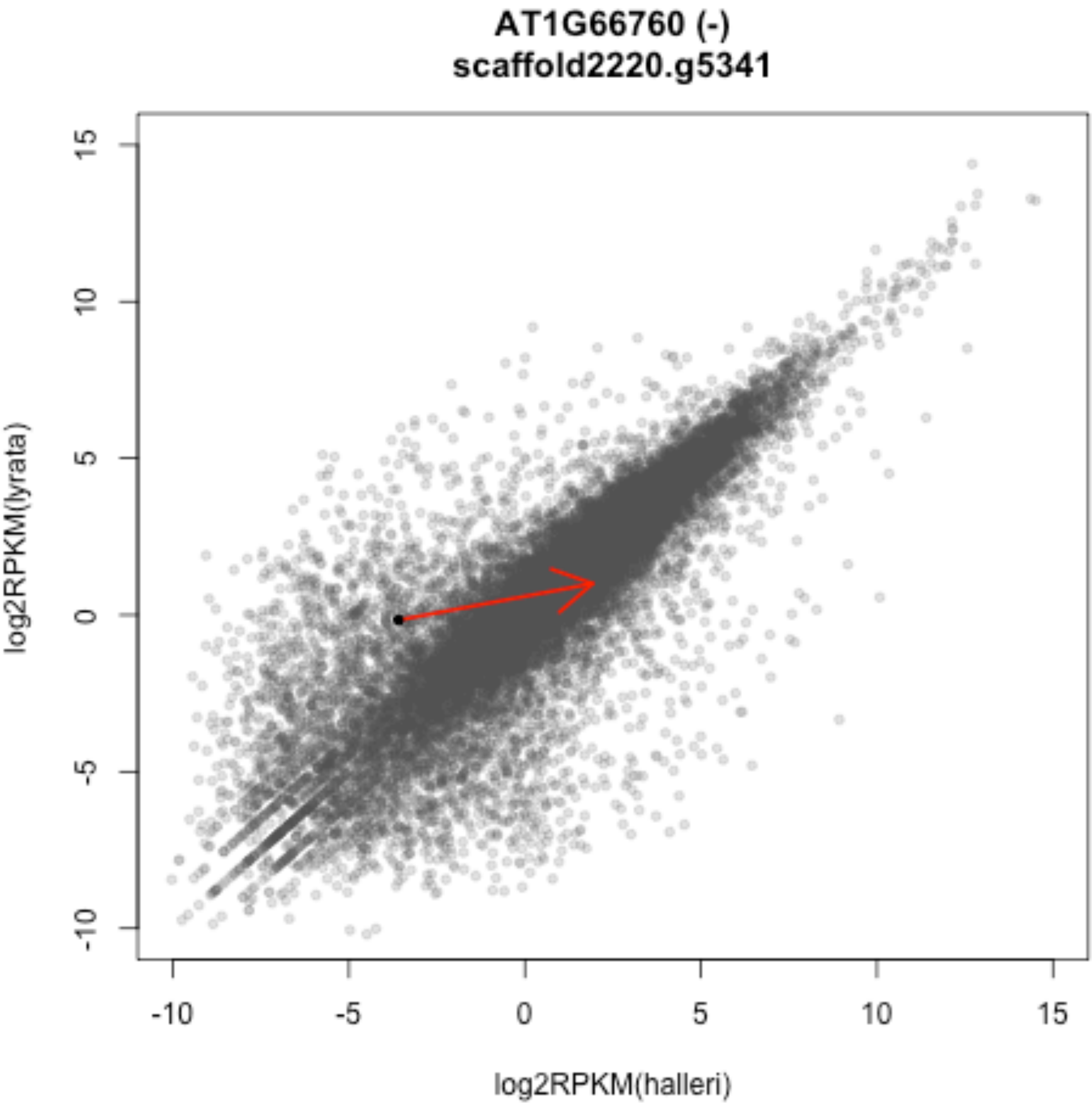

AT1G66760 (-)
scaffold2220.g5341
log2RPKM(lyrata)
log2RPKM(halleri)
-10
-5
0
5
10
15

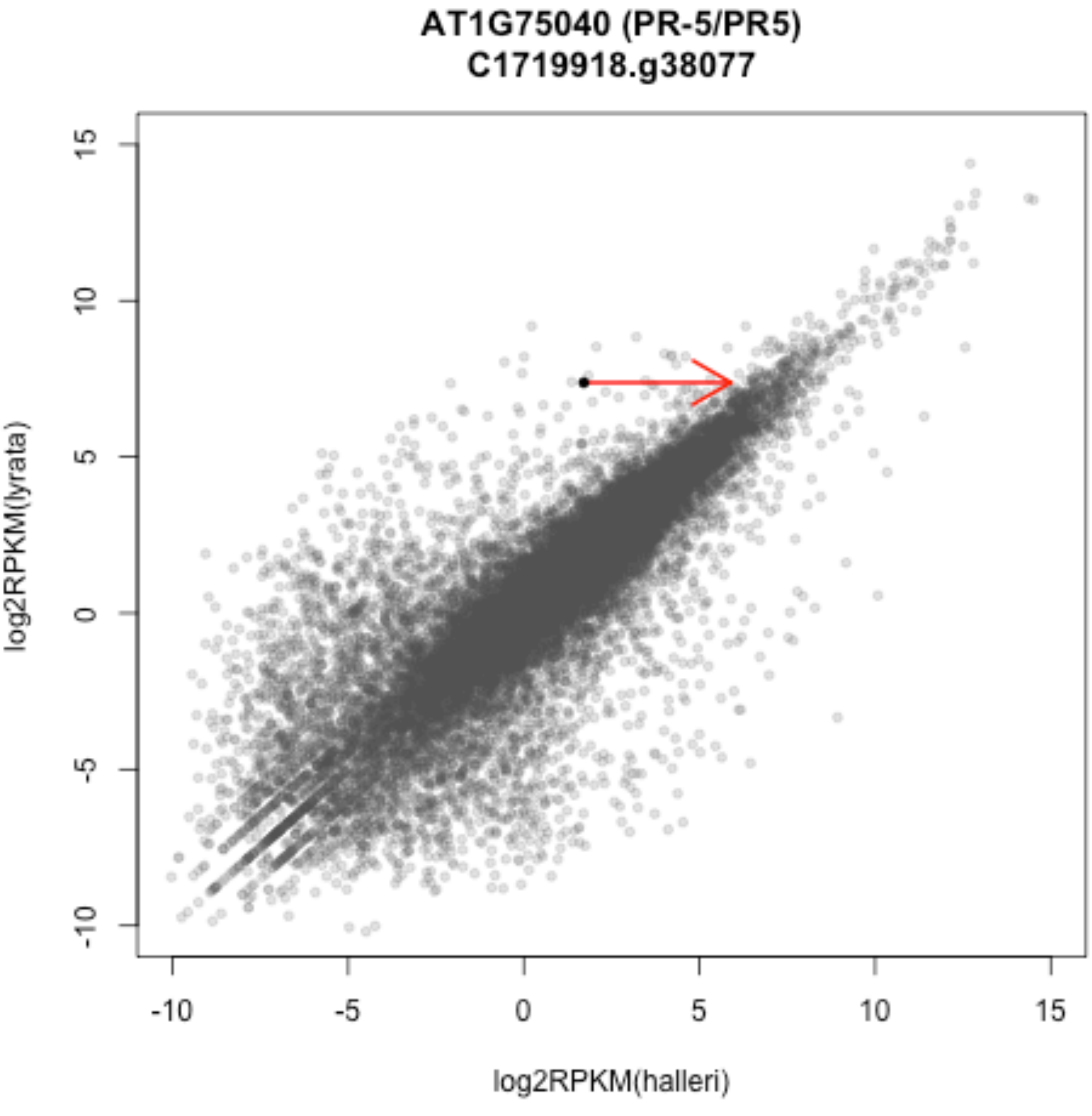

AT1G75040 (PR-5/PR5)
C1719918.g38077
log2RPKM(lyrata)
log2RPKM(halleri)
-10
-5
0
5
10
15

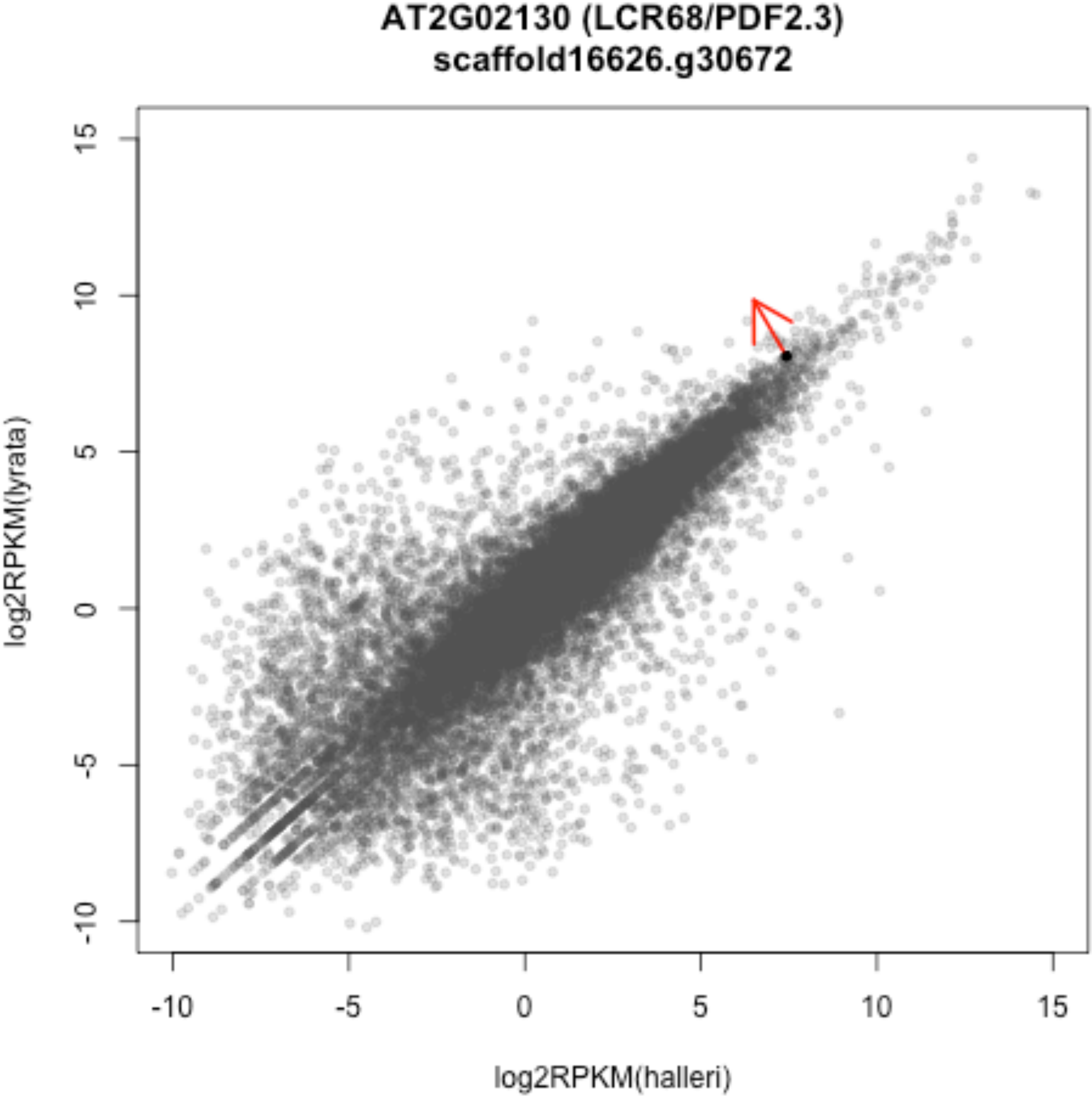
AT2G02130 (LCR68/PDF2.3)
scaffold16626.g30672
log2RPKM(lyrata)
log2RPKM(halleri)
15
10
5
0
-5
-10
-10
-5
0
5
10
15

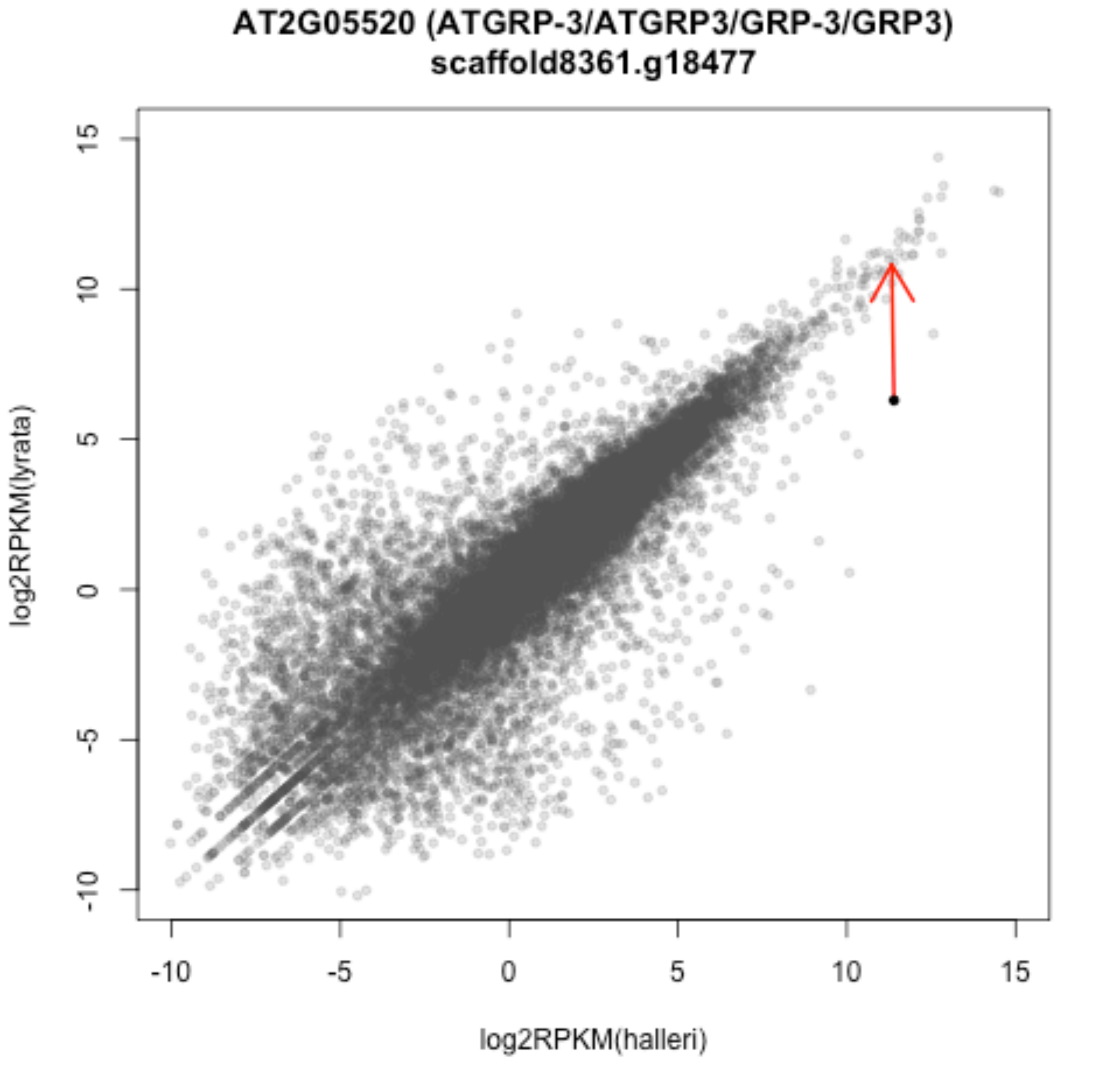
AT2G05520 (ATGRP-3/ATGRP3/GRP-3/GRP3)
scaffold8361.g18477
log2RPKM(lyrata)
log2RPKM(halleri)
-10
-5
0
5
10
15

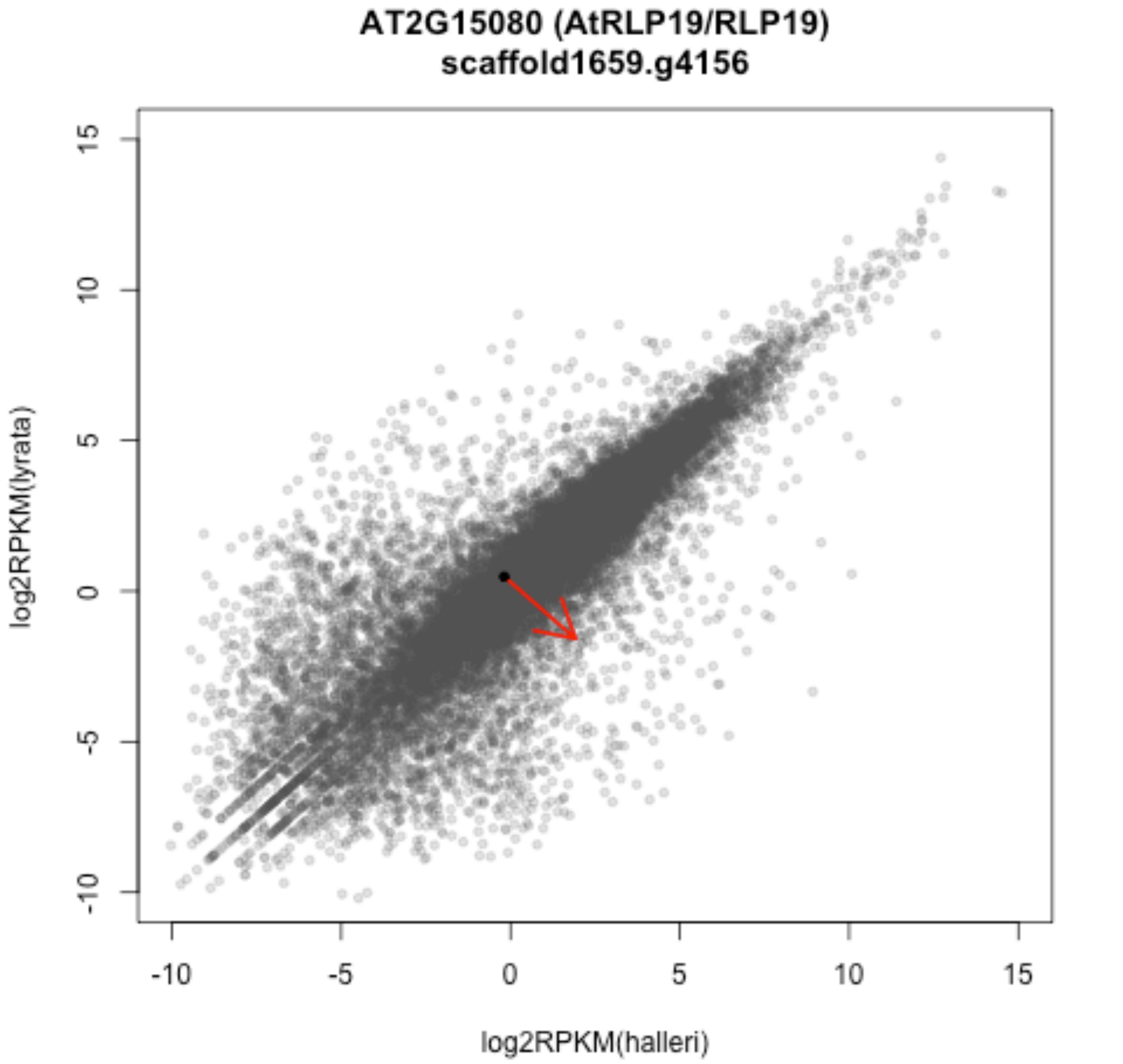
AT2G15080 (AtRLP19/RLP19)
scaffold1659.g4156
log2RPKM(lyrata)
log2RPKM(halleri)
-10
-5
0
5
10
15

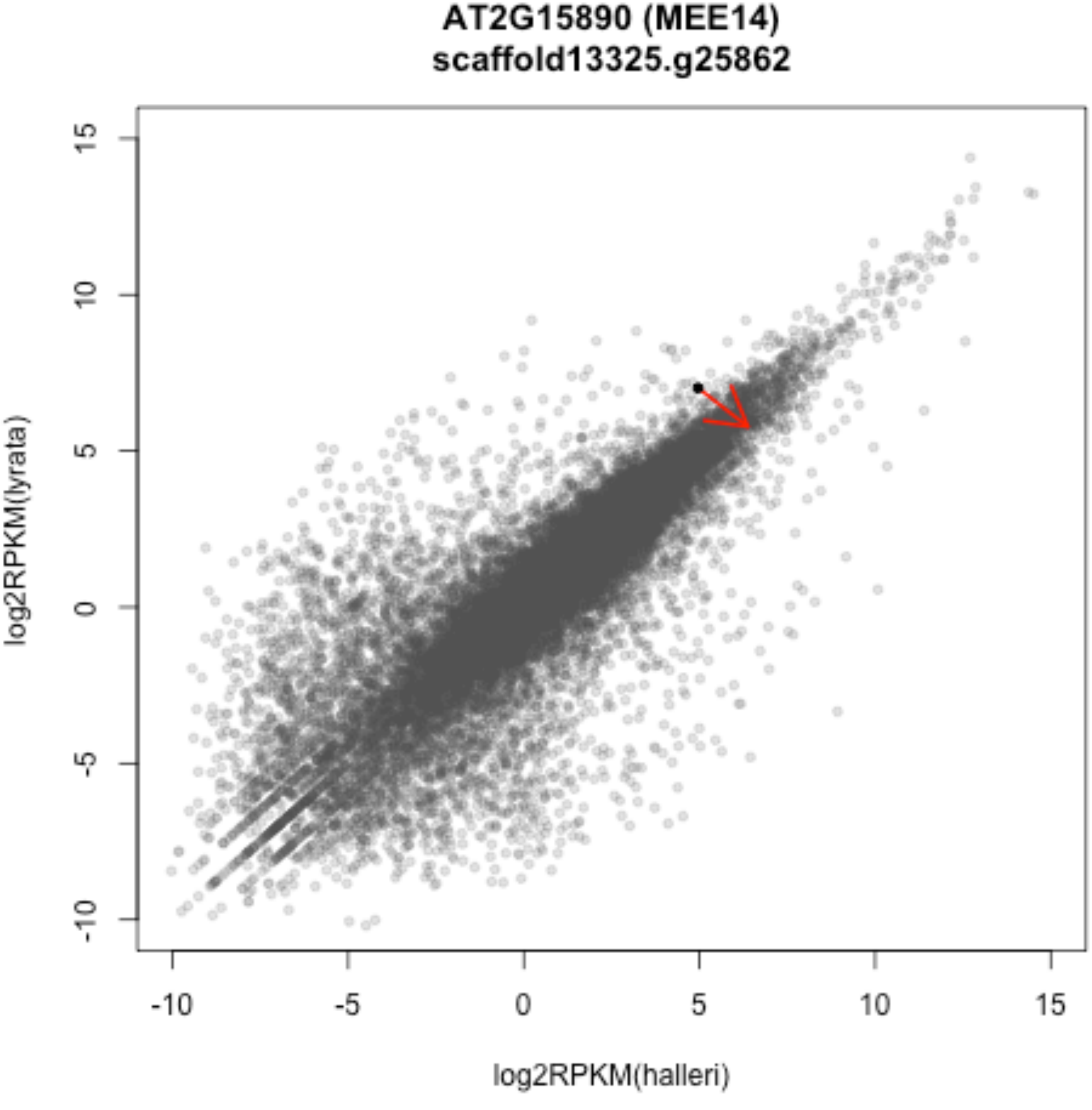
AT2G15890 (MEE14)
scaffold13325.g25862
log2RPKM(lyrata)
log2RPKM(halleri)
15
10
5
0
-5
-10
-10
-5
0
5
10
15

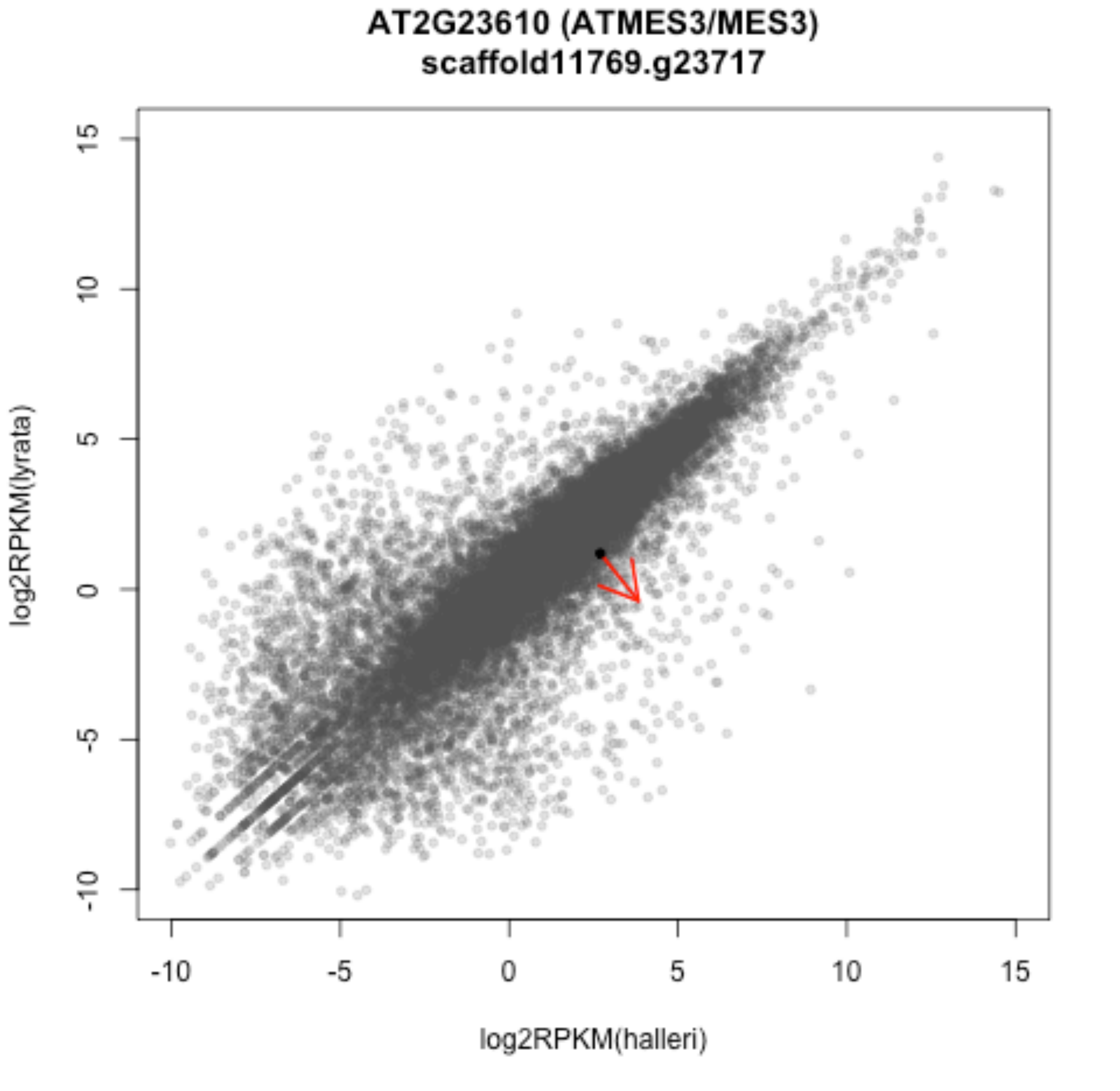

AT2G23610 (ATMES3/MES3)
scaffold11769.g23717
log2RPKM(lyrata)
log2RPKM(halleri)
-10
-5
0
5
10
15

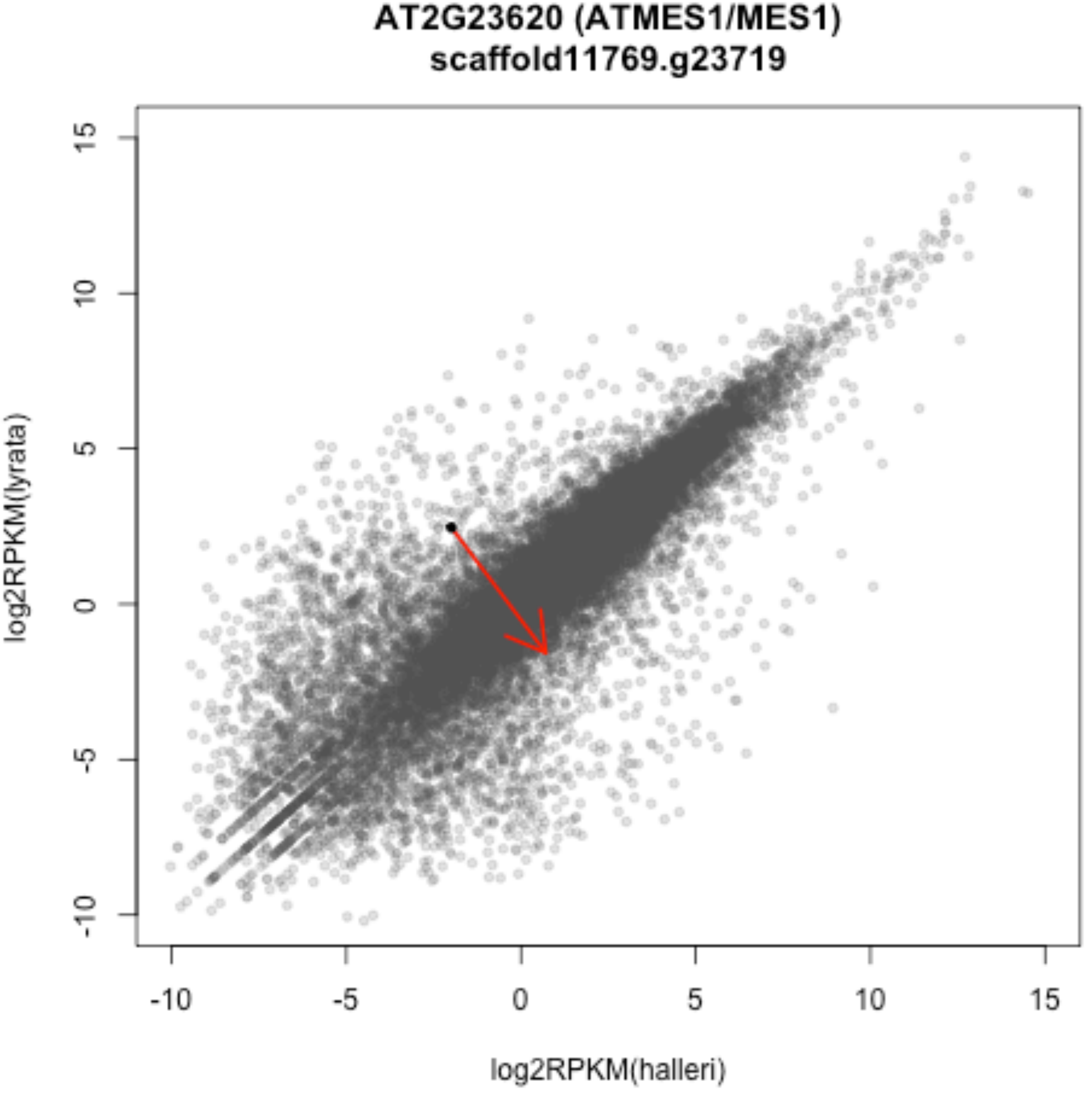
AT2G23620 (ATMES1/MES1)
scaffold11769.g23719
log2RPKM(lyrata)
log2RPKM(halleri)
15
10
5
0
-5
-10
-10
-5
0
5
10
15

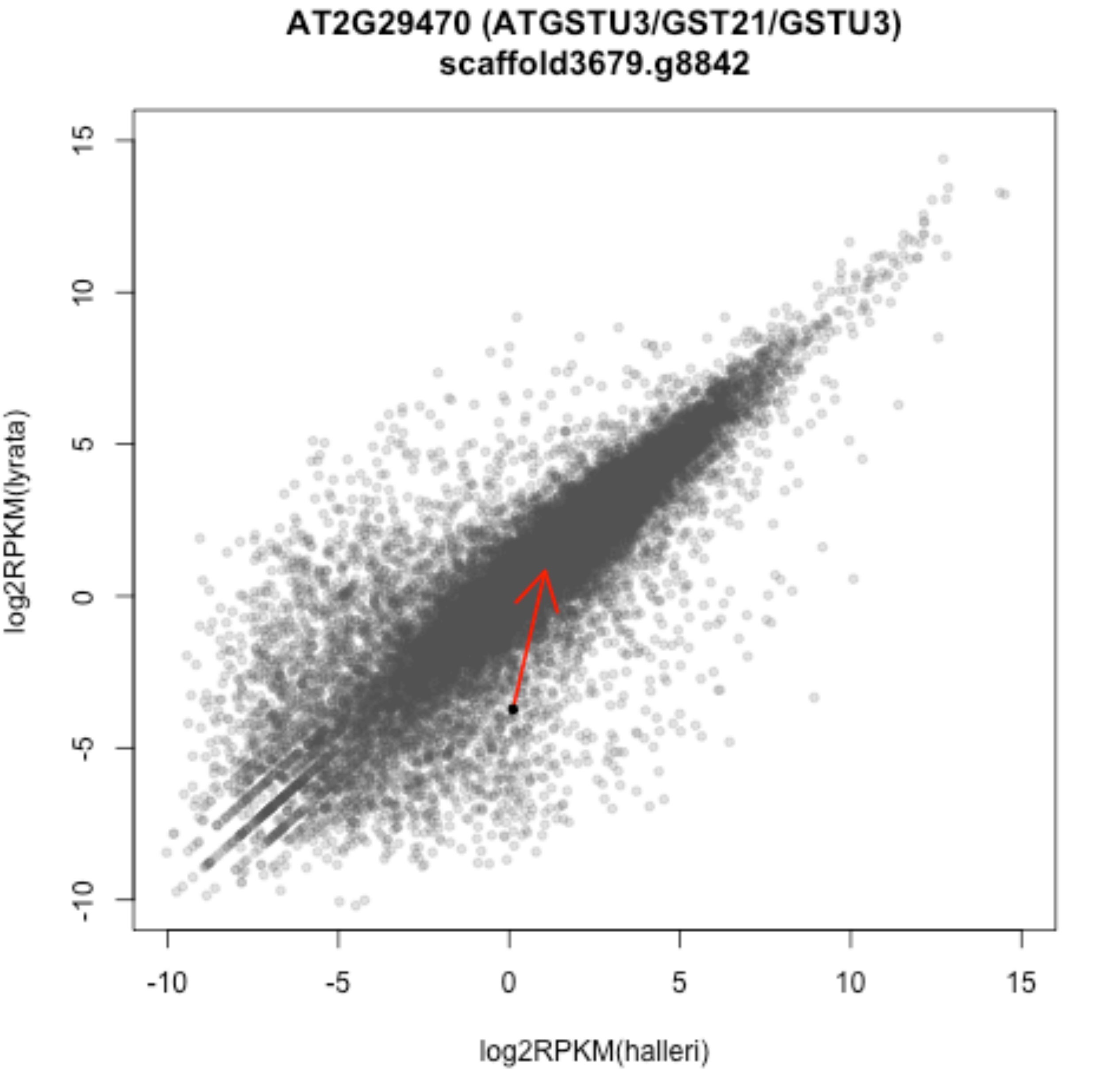
AT2G29470 (ATGSTU3/GST21/GSTU3)
scaffold3679.g8842
log2RPKM(lyrata)
log2RPKM(halleri)
15
10
5
0
-5
-10
-10
-5
0
5
10
15

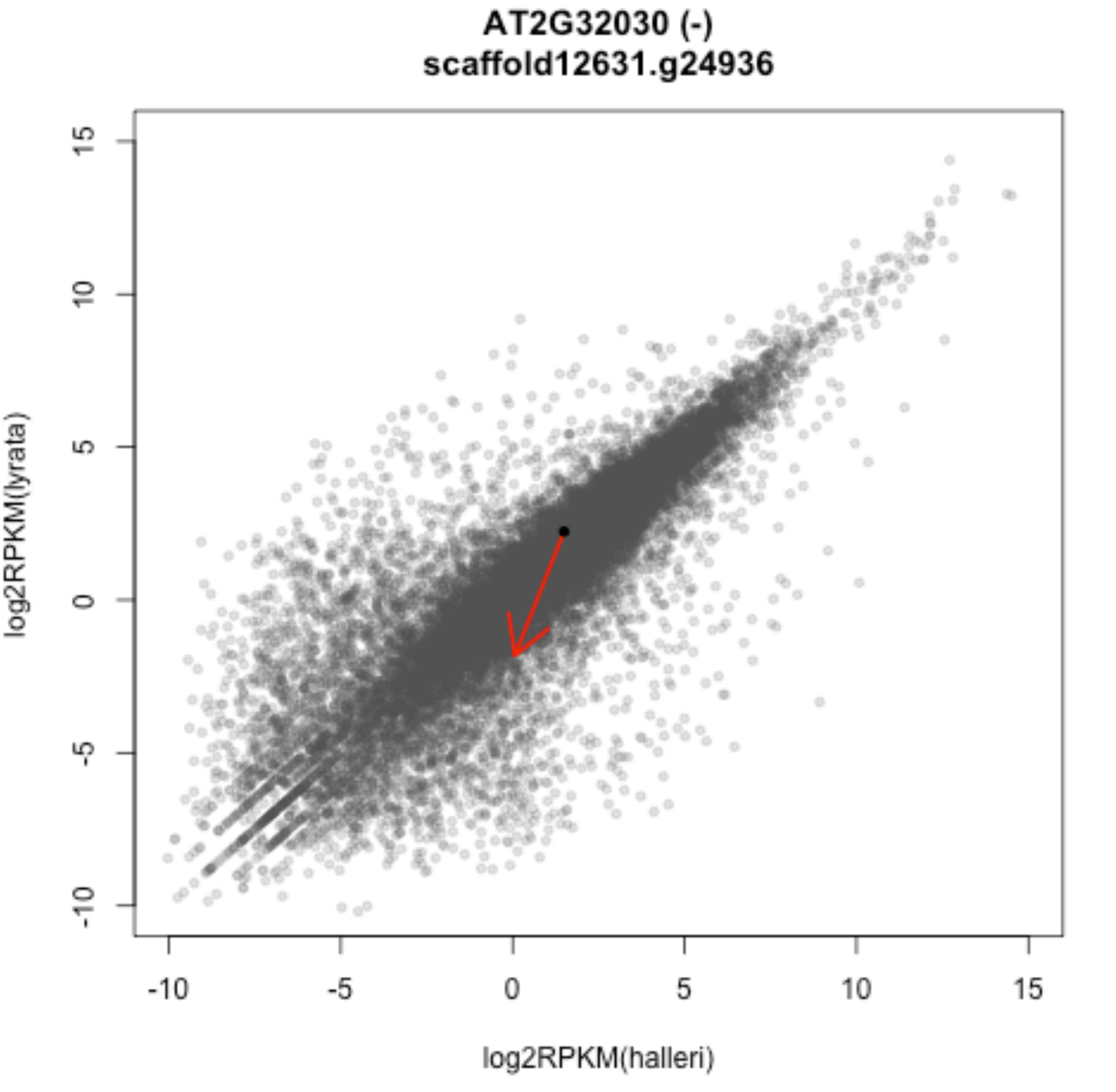
AT2G32030 (-)
scaffold12631.g24936
log2RPKM(lyrata)
log2RPKM(halleri)
-10
-5
0
5
10
15

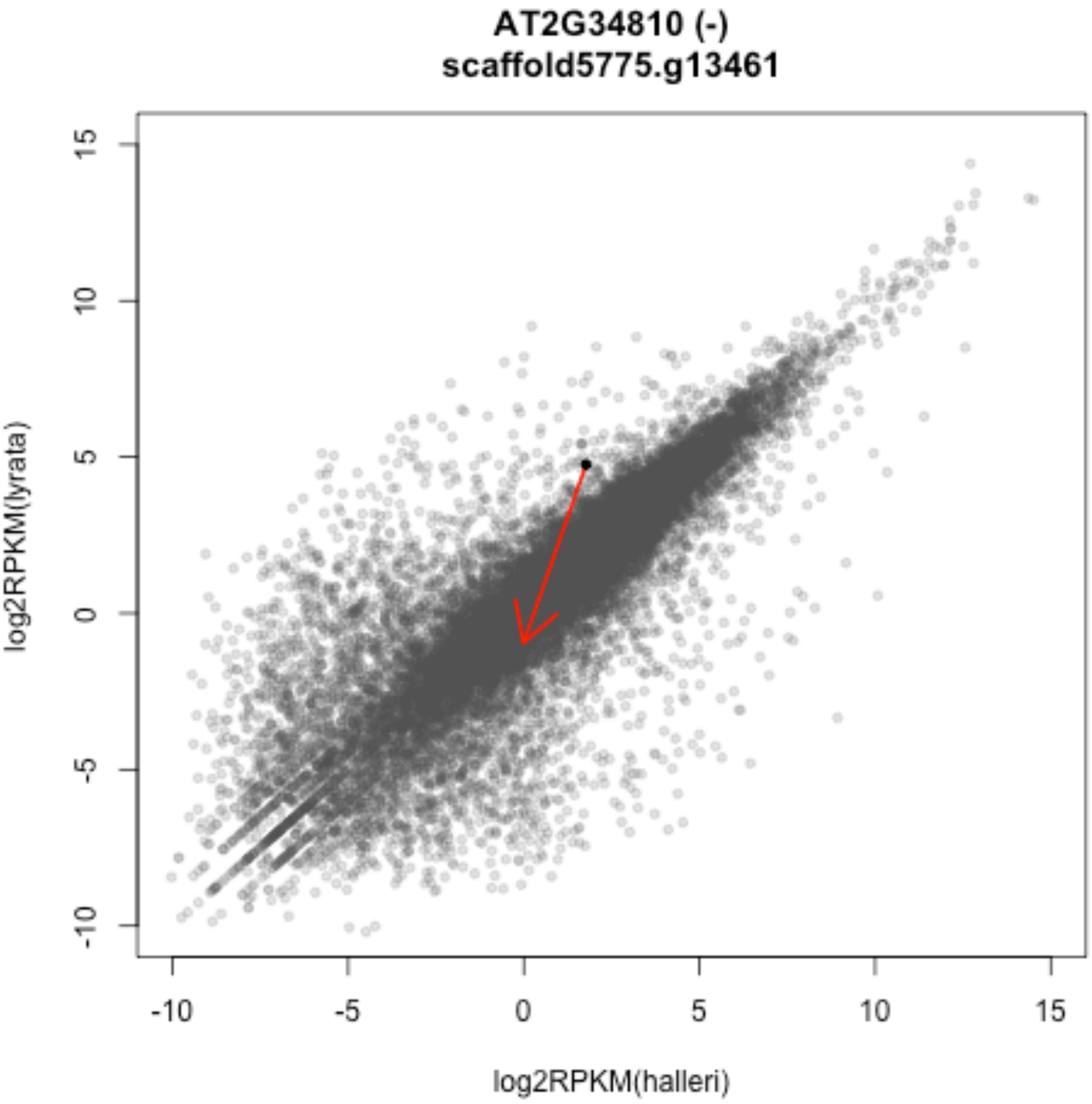

AT2G34810 (-)
scaffold5775.g13461
log2RPKM(lyrata)
log2RPKM(halleri)
15
10
5
0
-5
-10
-10
-5
0
5
10
15

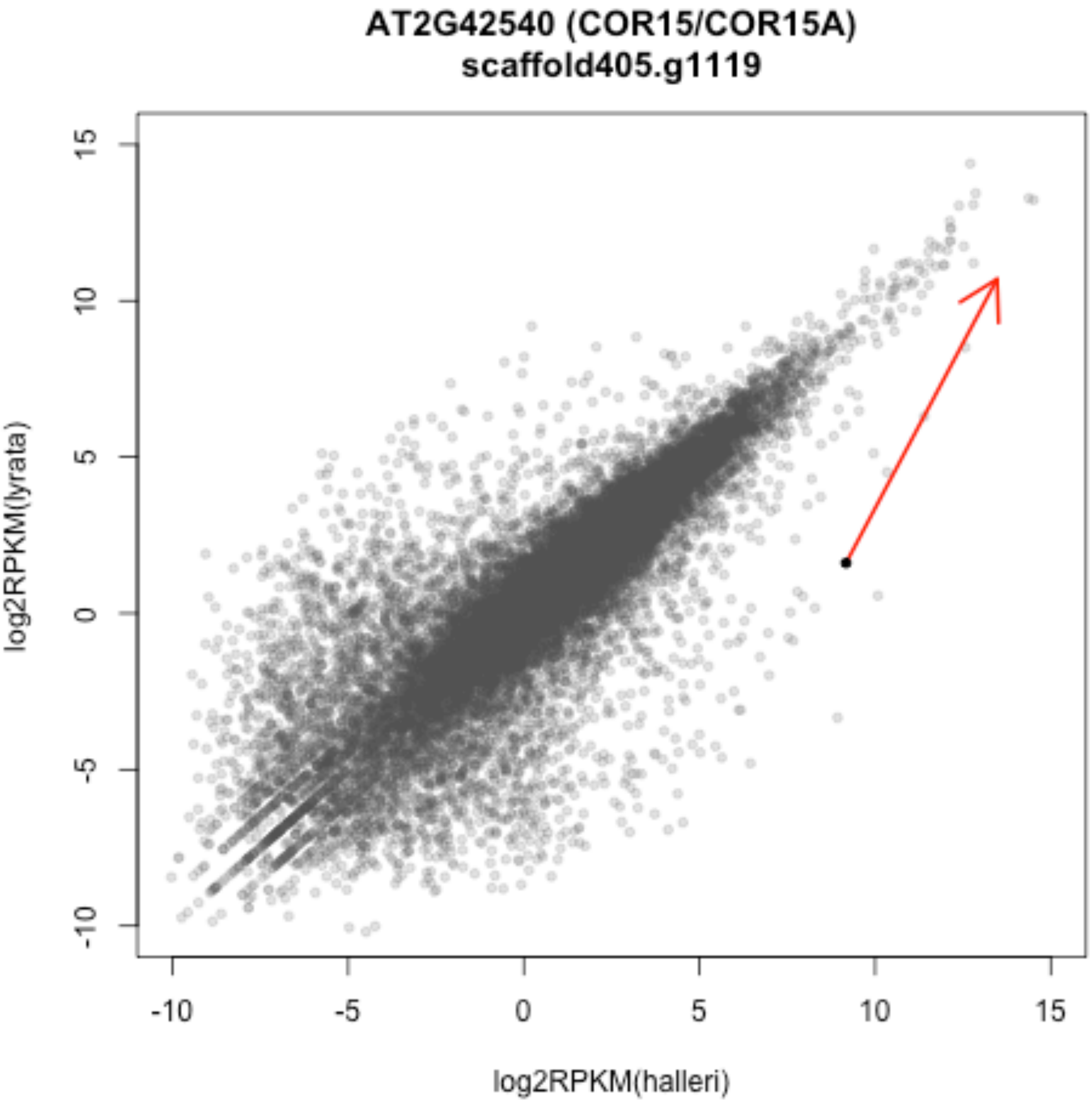
AT2G42540 (COR15/COR15A)
scaffold405.g1119
log2RPKM(lyrata)
log2RPKM(halleri)
-10
-5
0
5
10
15

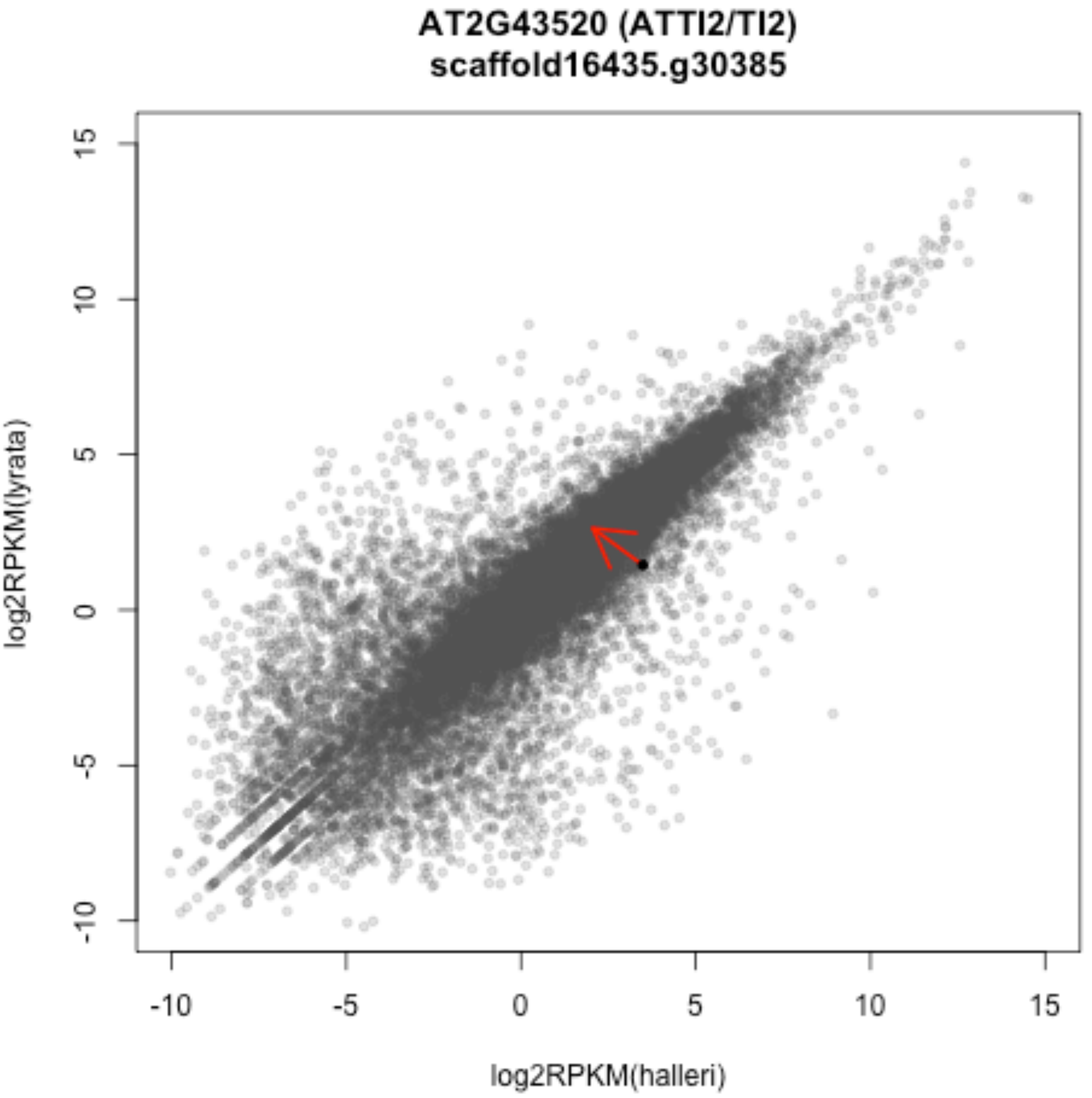
AT2G43520 (ATTI2/TI2)
scaffold16435.g30385
log2RPKM(lyrata)
log2RPKM(halleri)
-10
-5
0
5
10
15

AT2G47770 (ATTSPO/TSPO)
scaffold11473.g23231

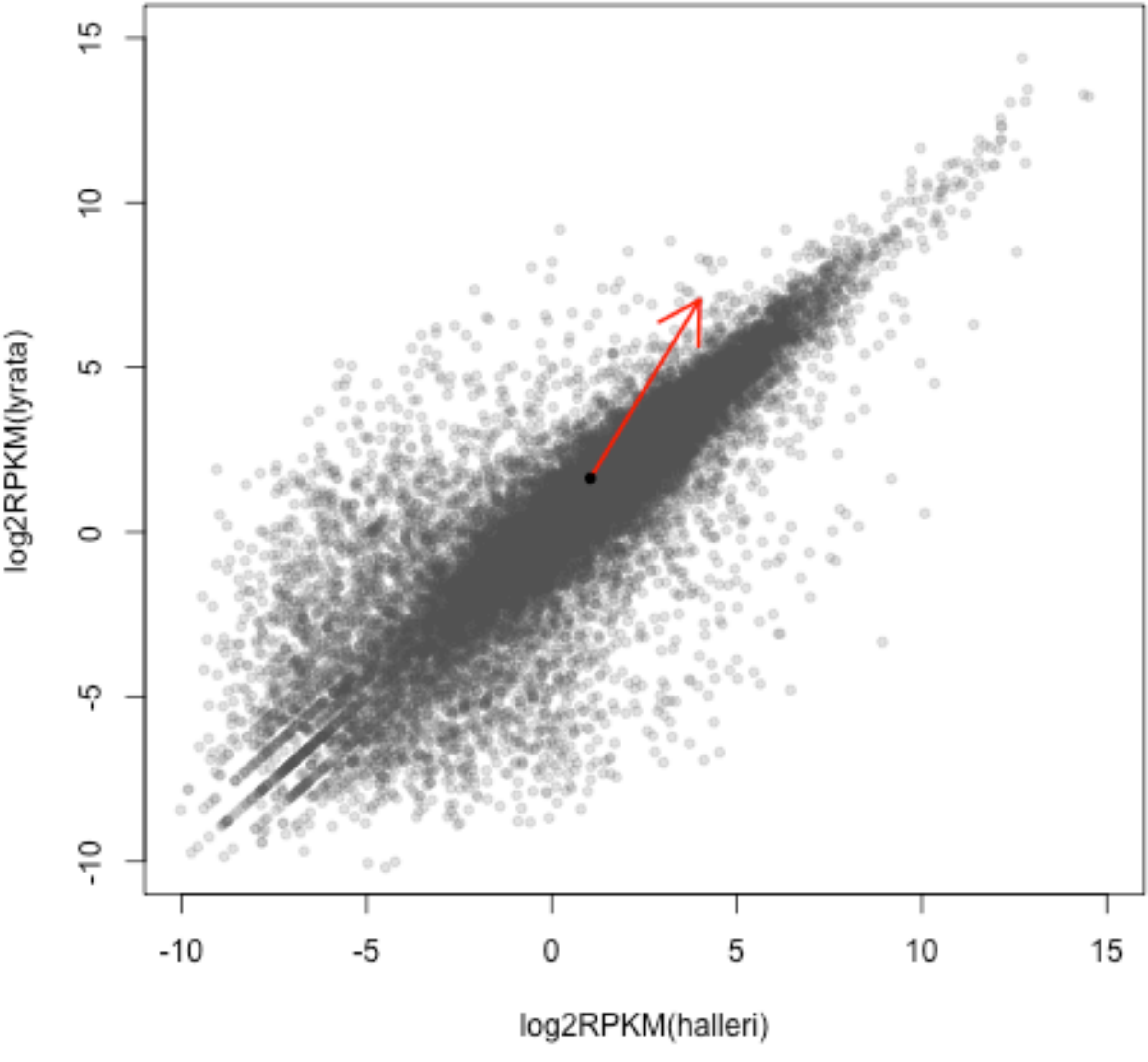

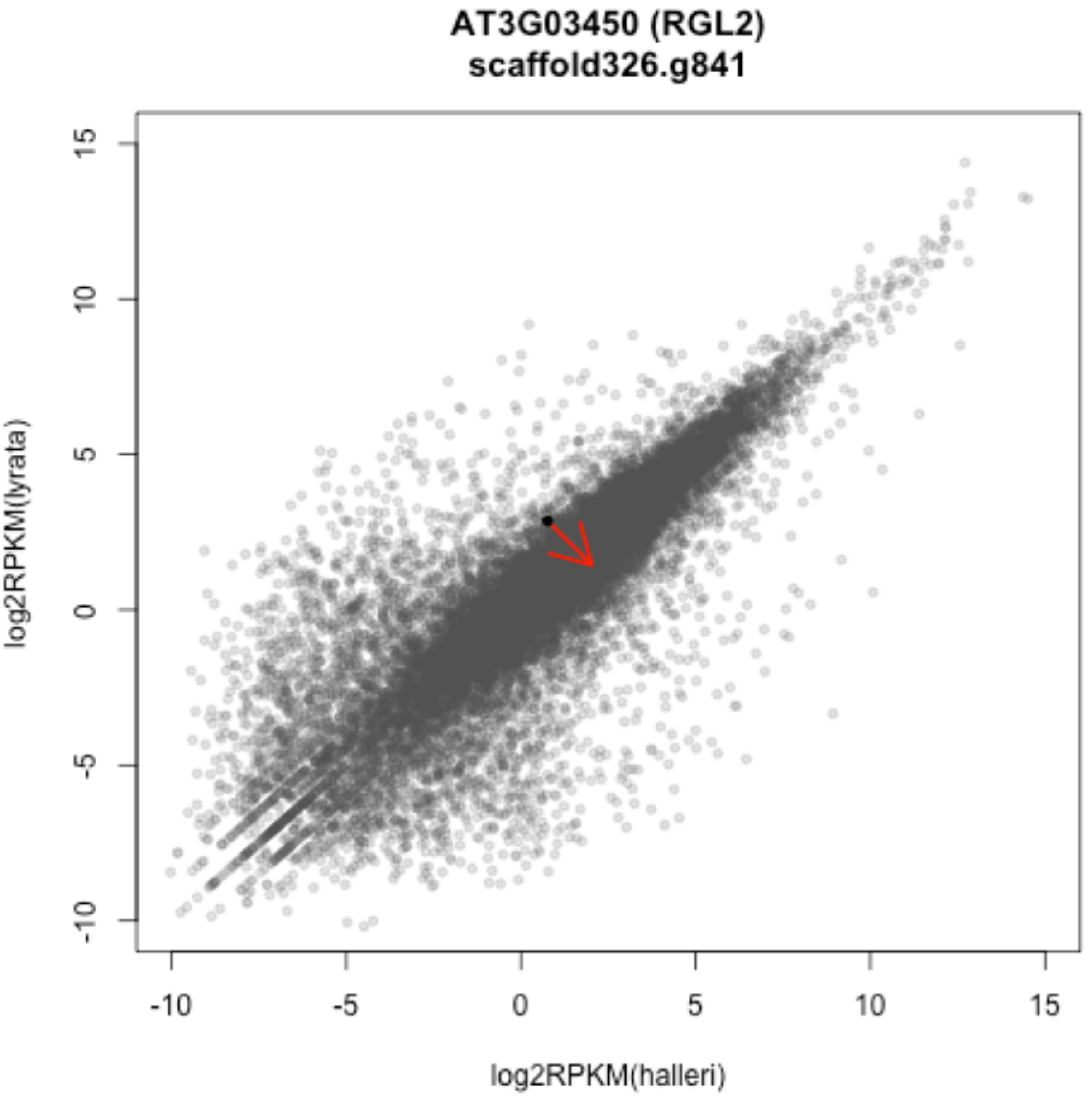
AT3G03450 (RGL2)
scaffold326.g841
log2RPKM(lyrata)
log2RPKM(halleri)
15
10
5
0
-5
-10
-10
-5
0
5
10
15

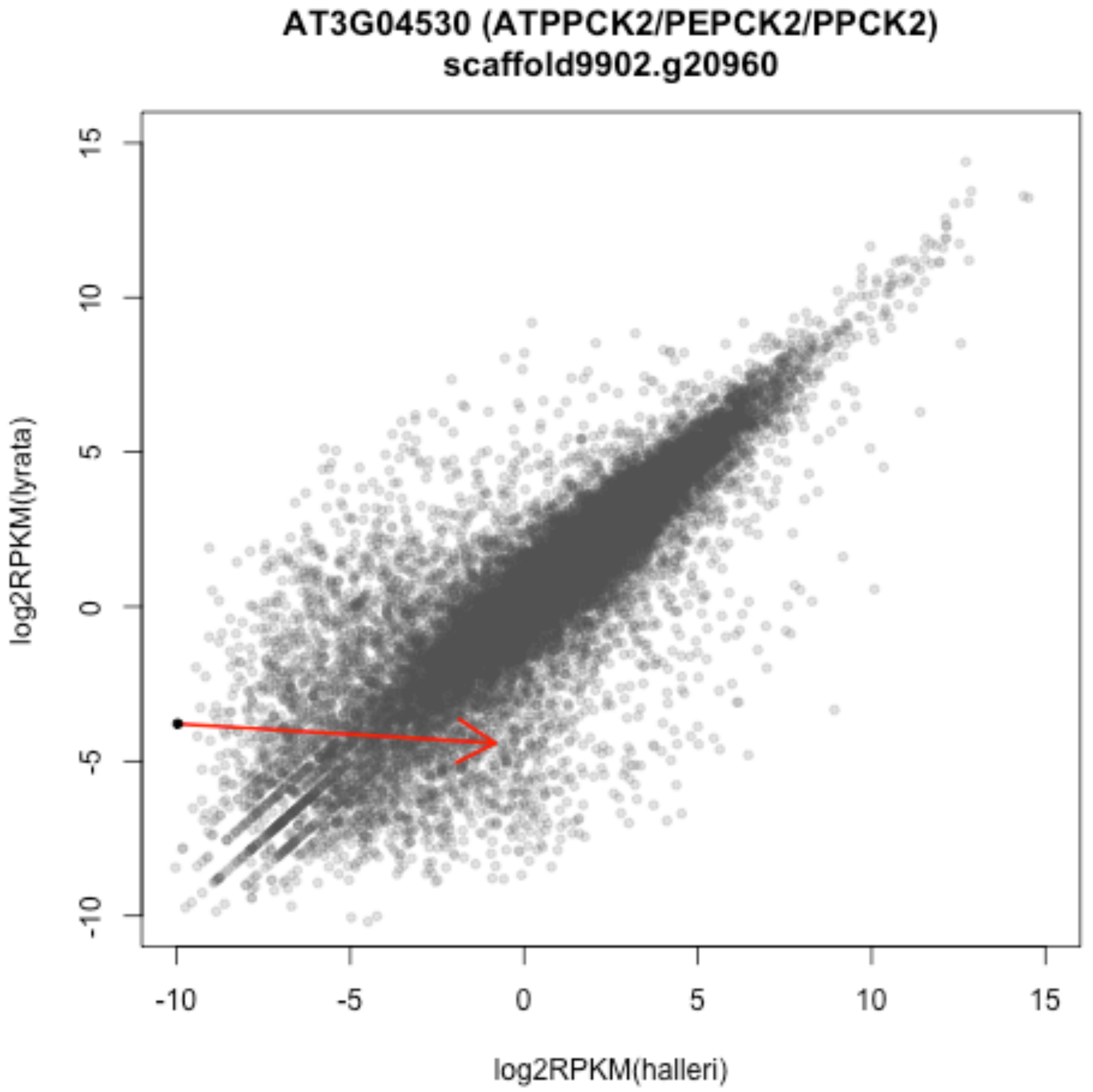
AT3G04530 (ATPPCK2/PEPCK2/PPCK2)
scaffold9902.g20960
log2RPKM(lyrata)
log2RPKM(halleri)
15
10
5
0
-5
-10
-10
-5
0
5
10
15

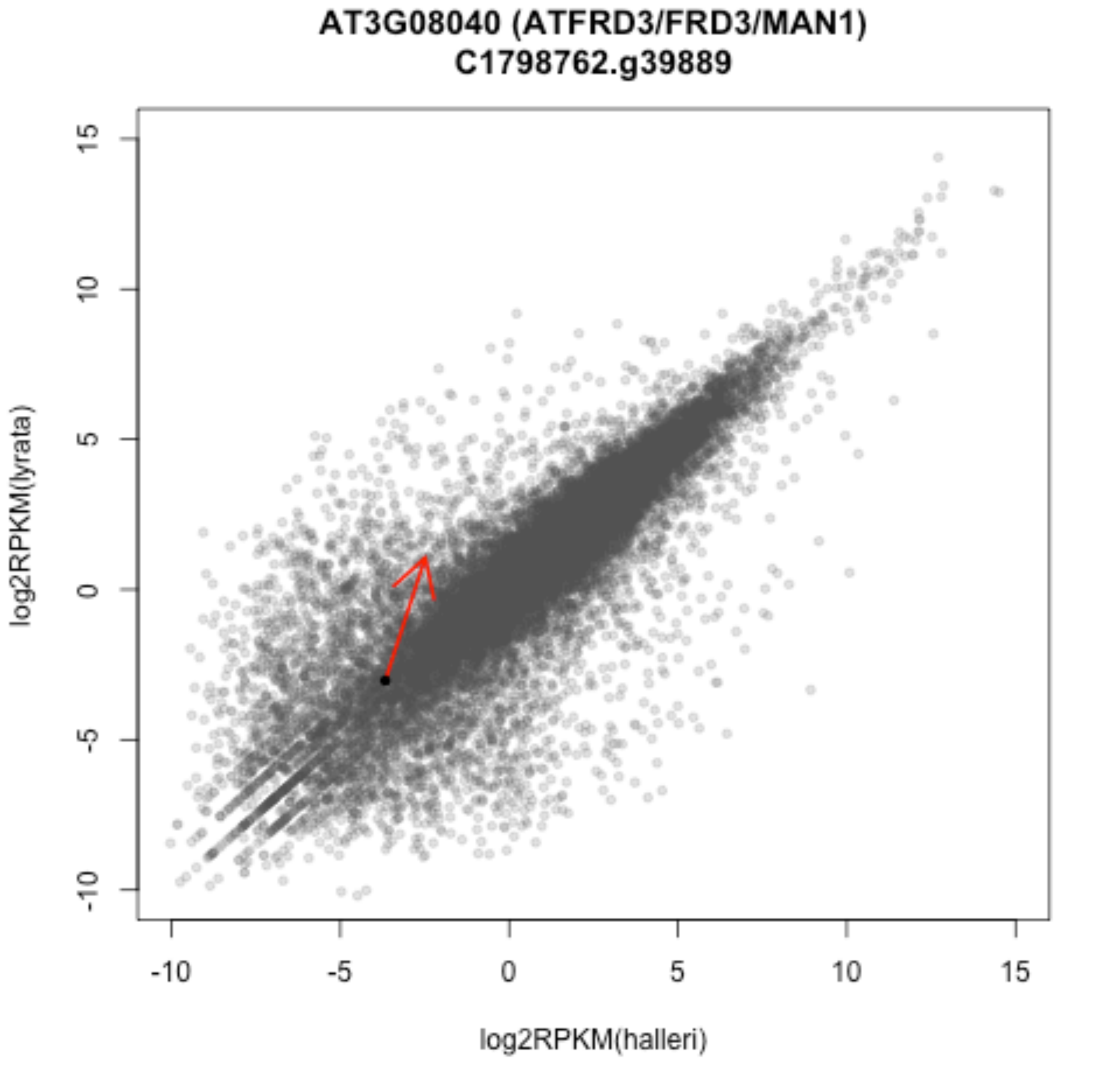
AT3G08040 (ATFRD3/FRD3/MAN1)
C1798762.g39889
log2RPKM(lyrata)
log2RPKM(halleri)
15
10
5
0
-5
-10
-10
-5
0
5
10
15

AT3G22370 (AOX1A/ATAOX1A/AtHSR3/HSR3)
scaffold1631.g4077

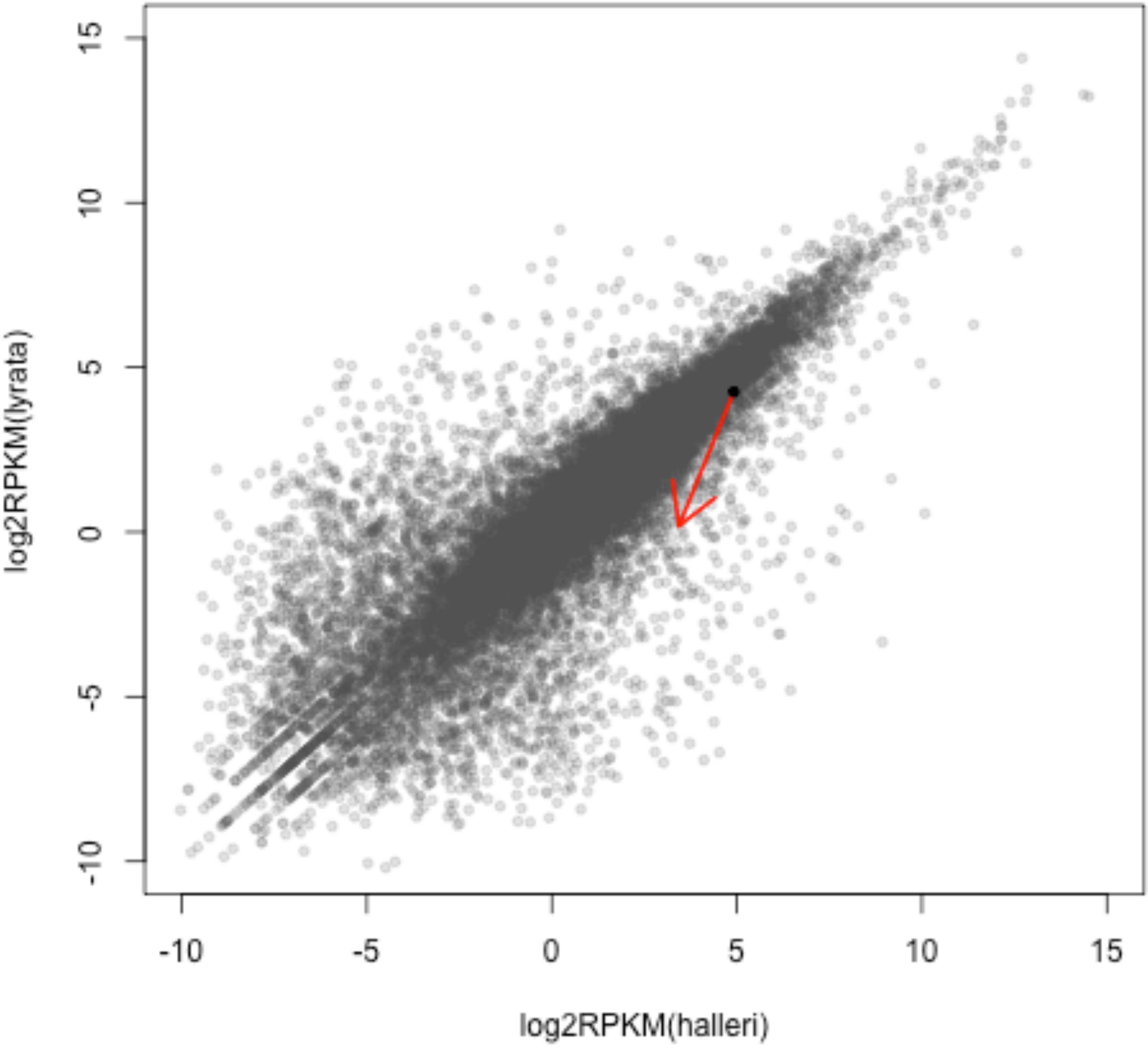

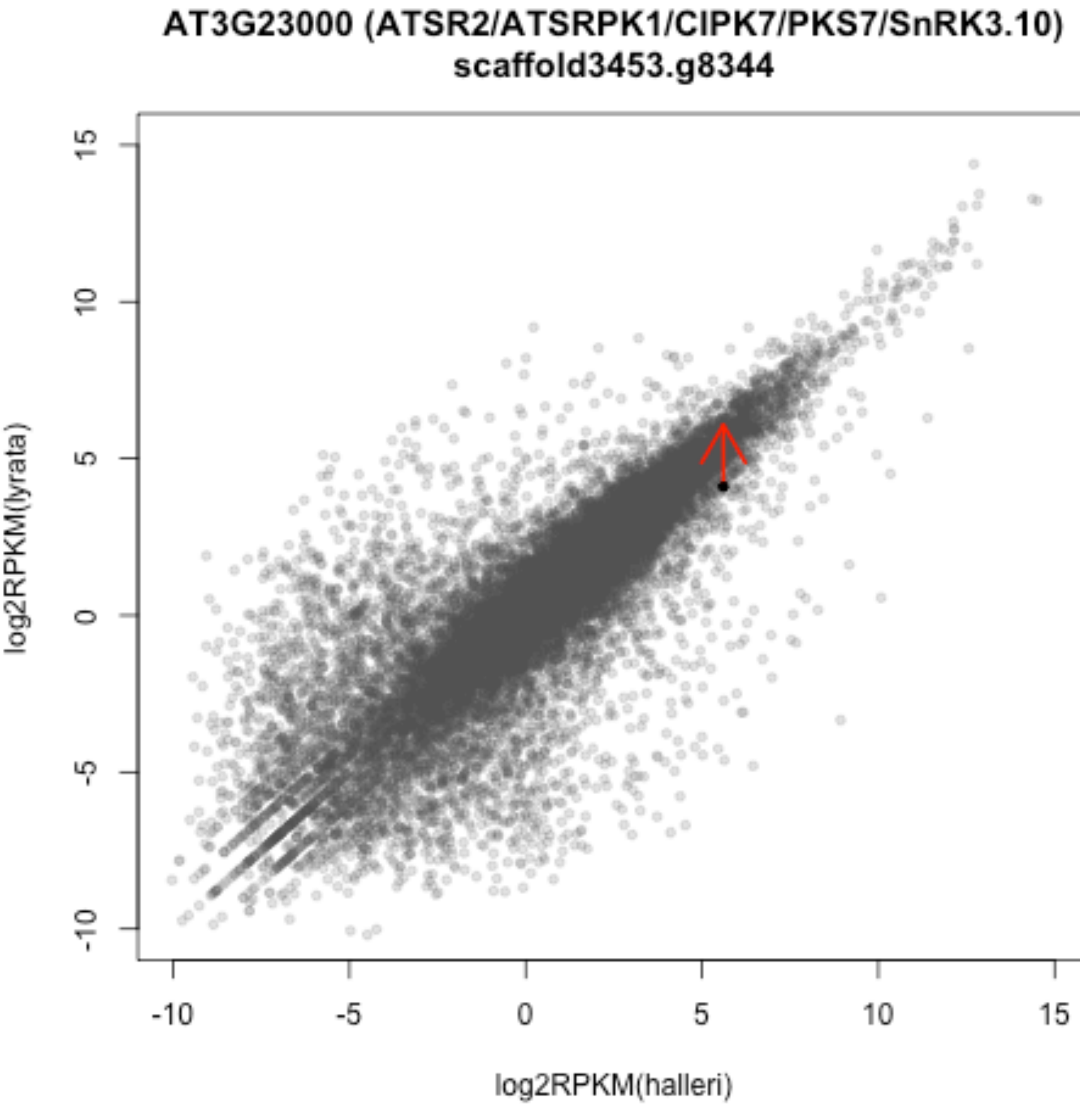

AT3G23000 (ATSR2/ATSRPK1/CIPK7/PKS7/SnRK3.10)
scaffold3453.g8344
log2RPKM(lyrata)
log2RPKM(halleri)
15
10
5
0
-5
-10
-10
-5
0
5
10
15

AT3G26520 (GAMMA-TIP2/SITIP/TIP1;2/TIP2)
scaffold2498.g5985

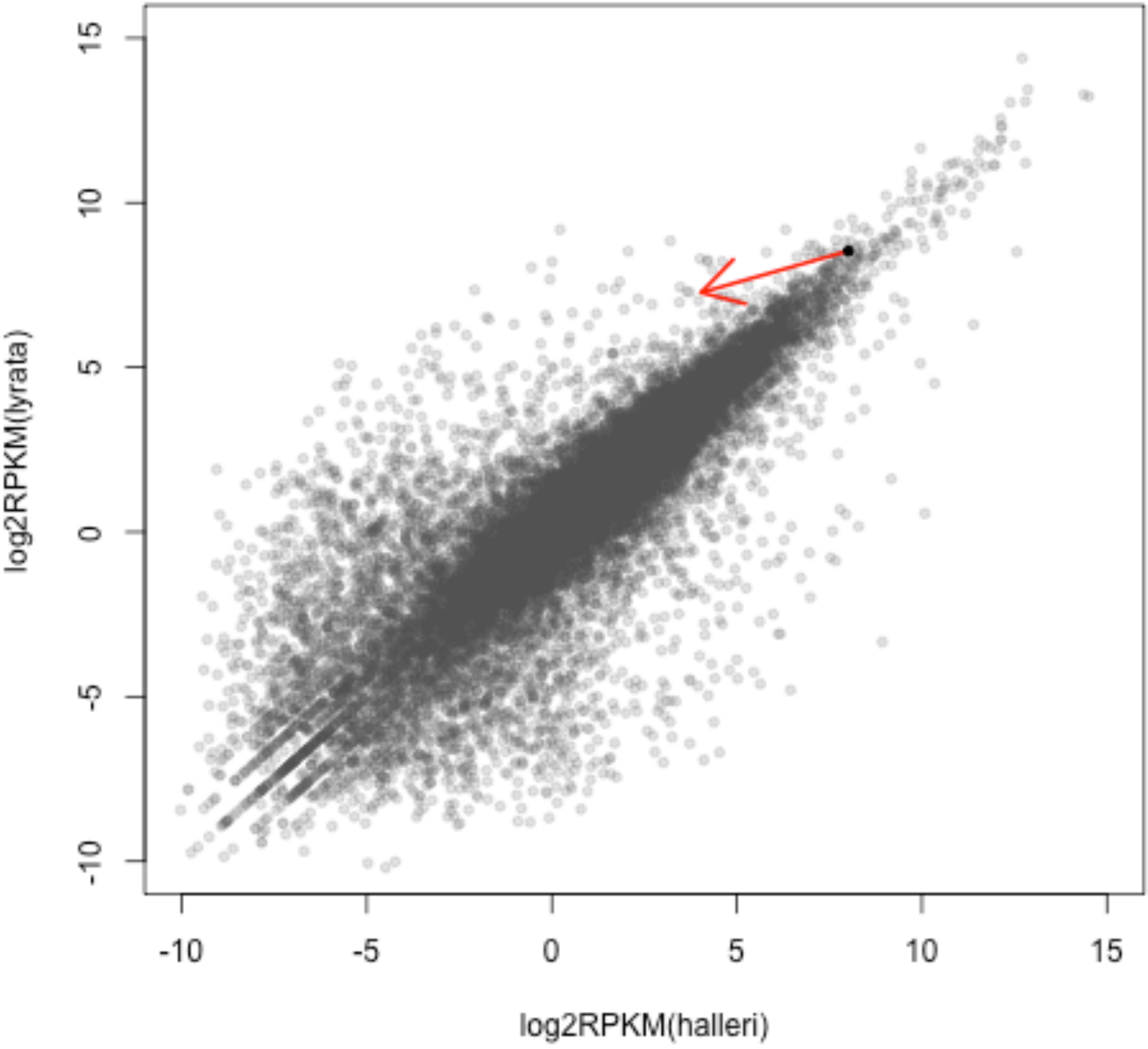

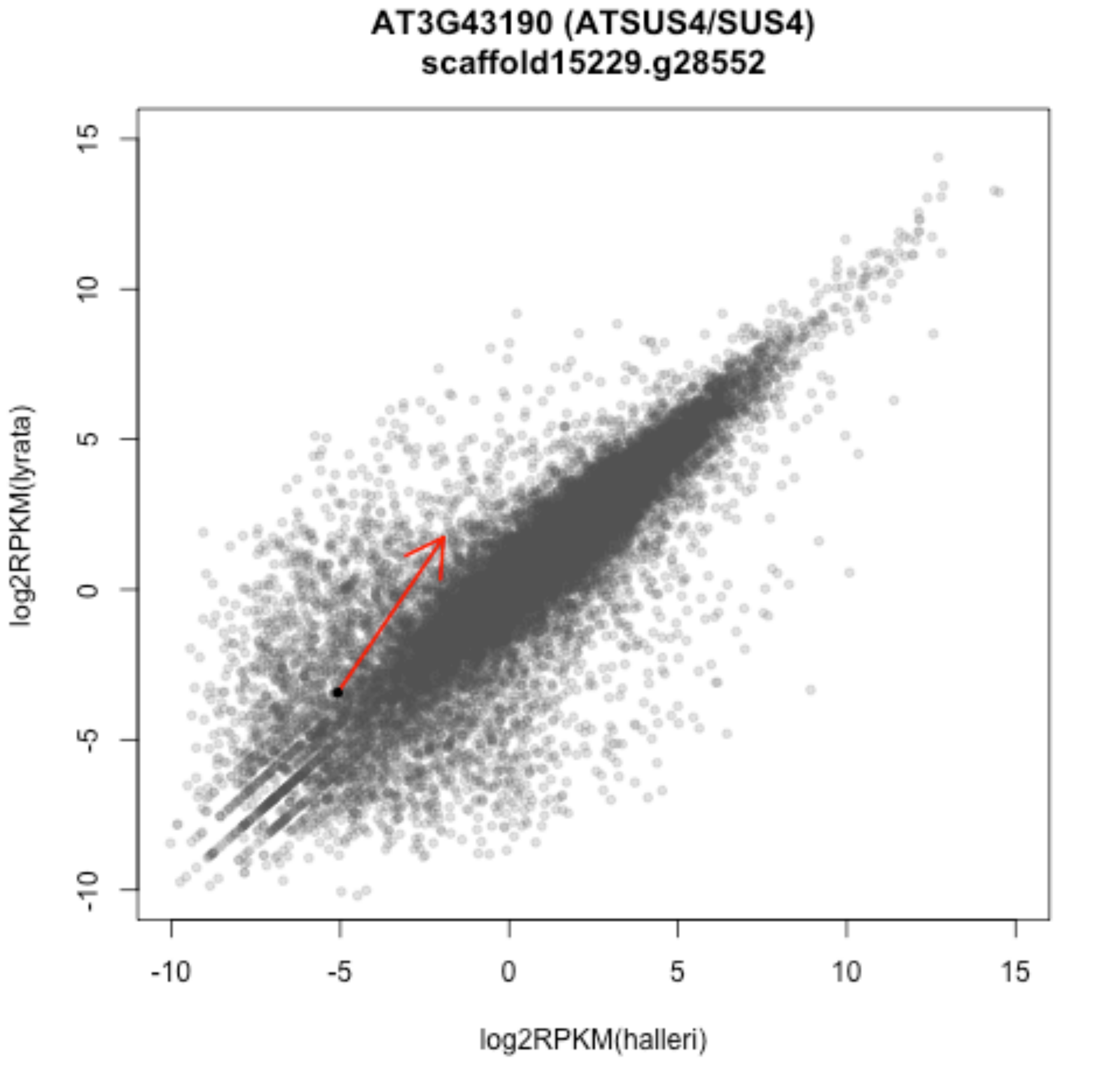

AT3G43190 (ATSUS4/SUS4)
scaffold15229.g28552
log2RPKM(lyrata)
log2RPKM(halleri)
-10
-5
0
5
10
15

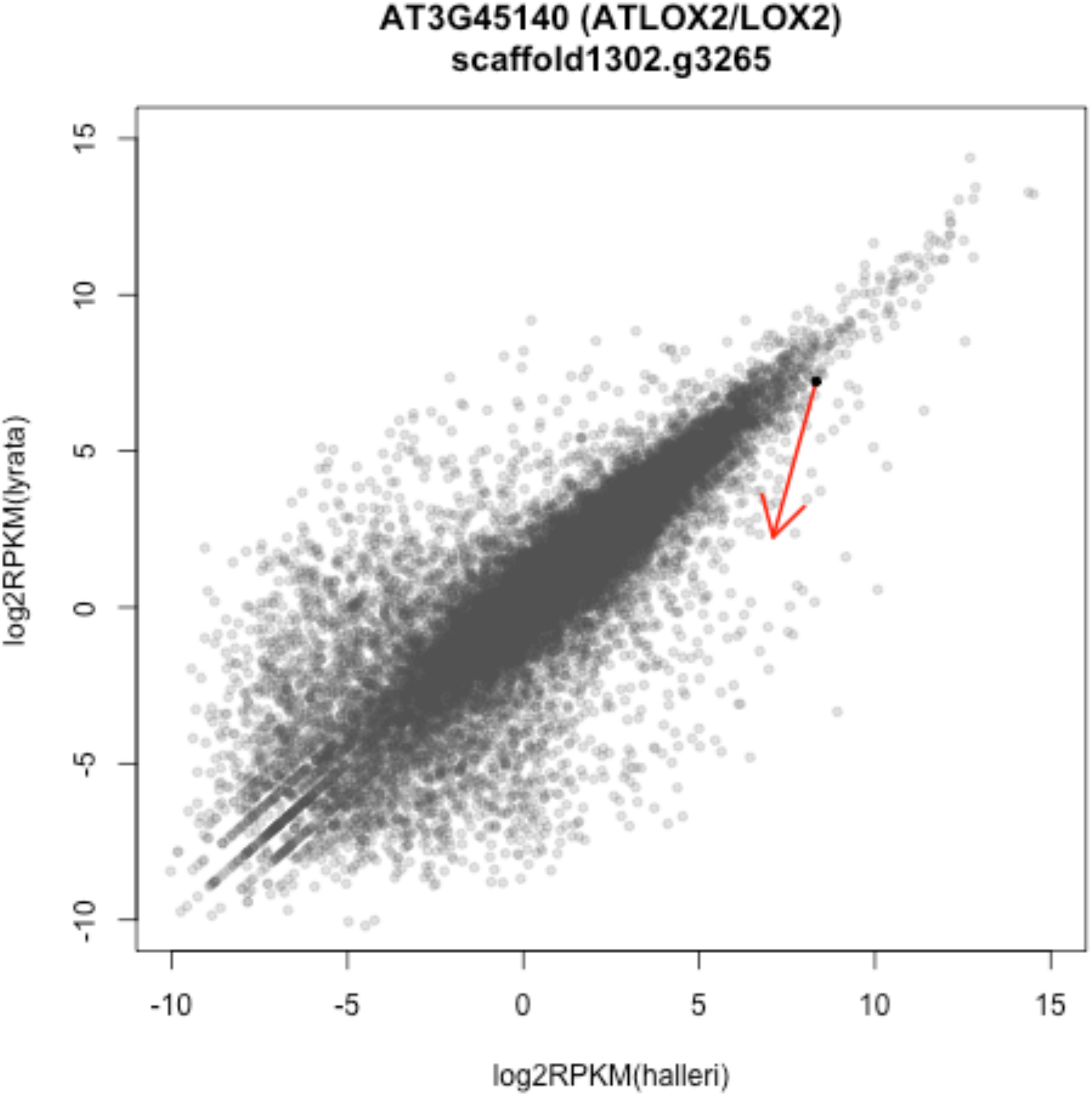
AT3G45140 (ATLOX2/LOX2)
scaffold1302.g3265
log2RPKM(lyrata)
log2RPKM(halleri)
15
10
5
0
-5
-10
-10
-5
0
5
10
15

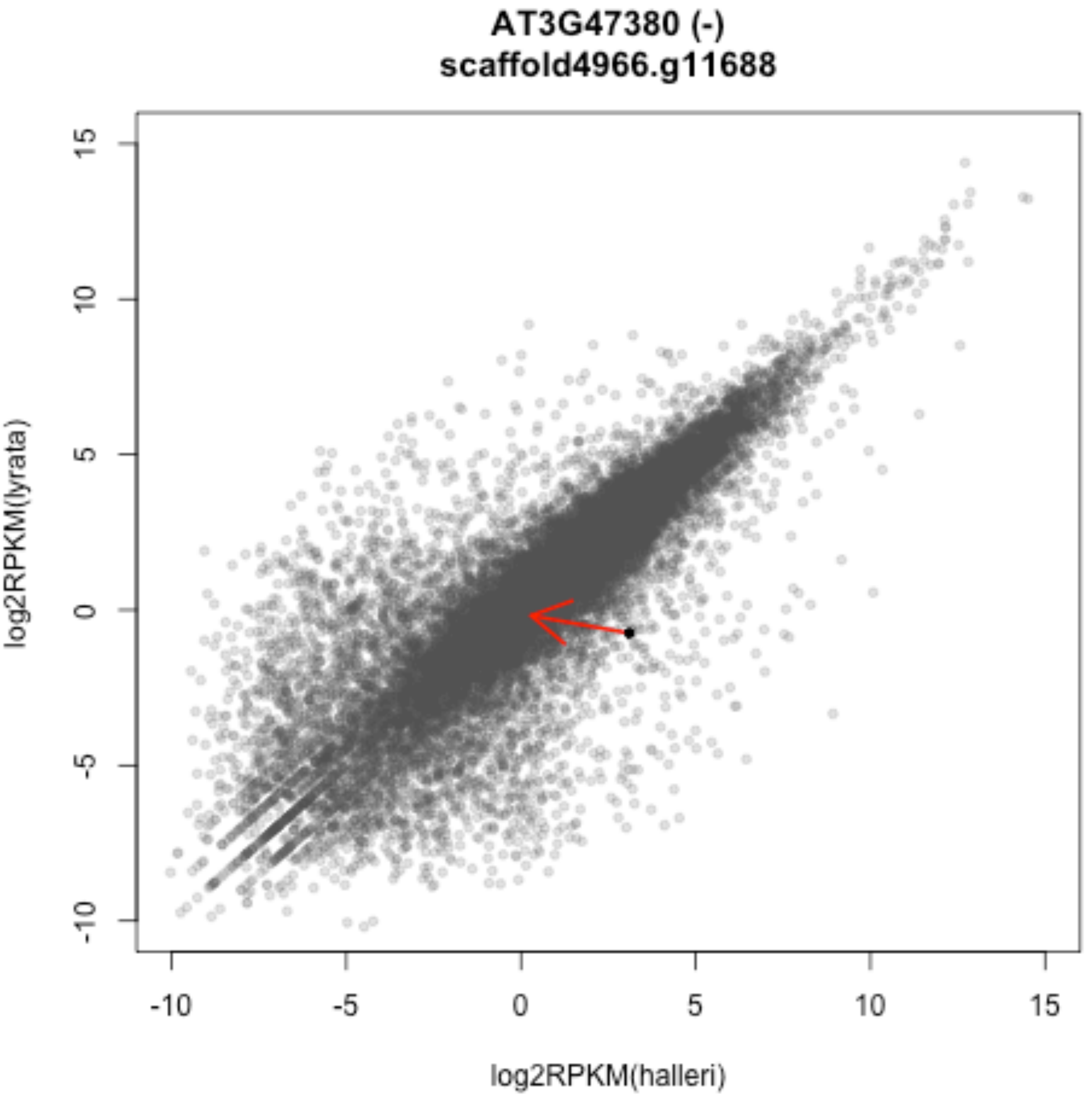
AT3G47380 (-)
scaffold4966.g11688
log2RPKM(lyrata)
log2RPKM(halleri)
-10
-5
0
5
10
15

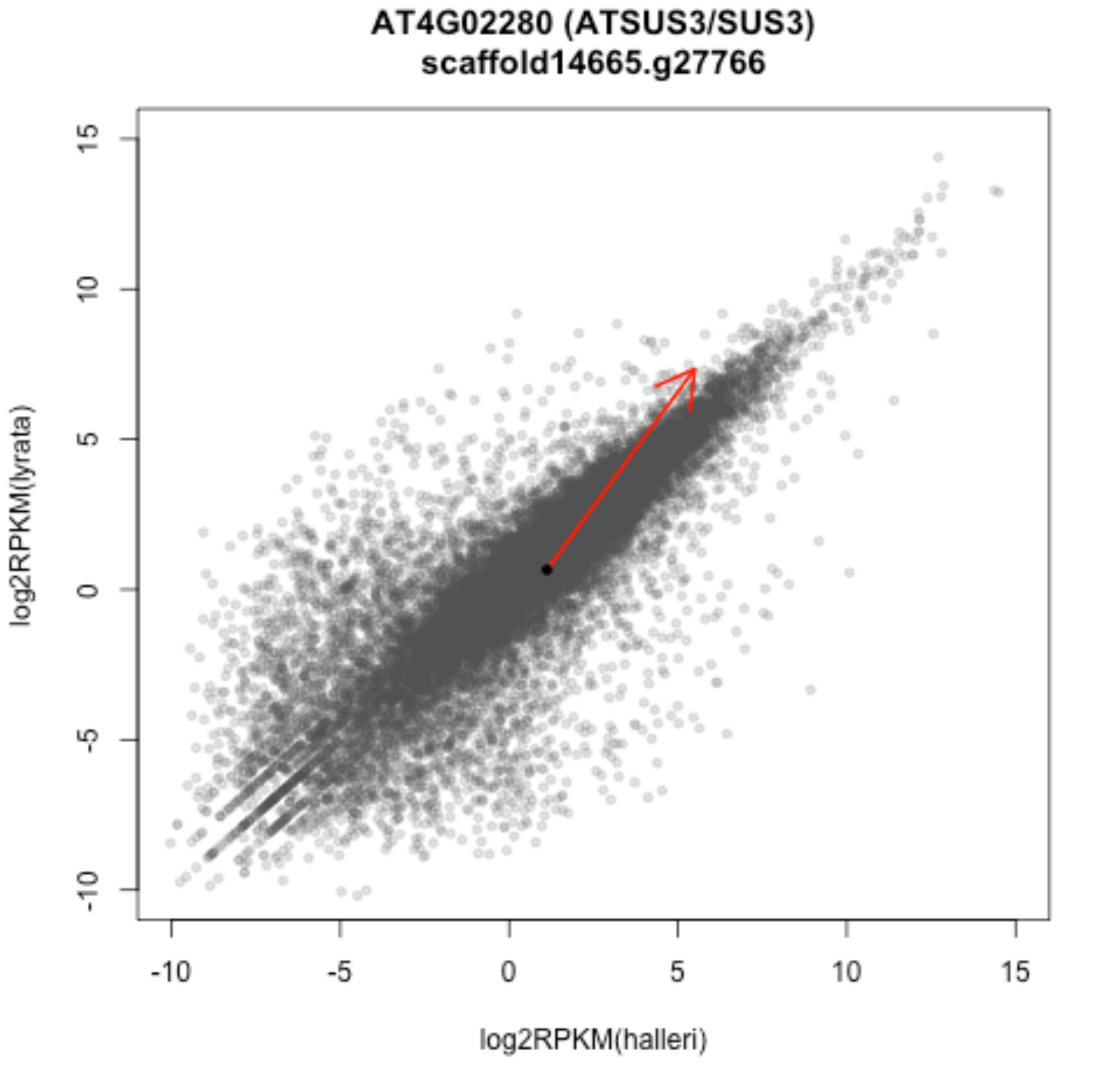

AT4G02280 (ATSUS3/SUS3)
scaffold14665.g27766
log2RPKM(lyrata)
log2RPKM(halleri)
-10
-5
0
5
10
15

AT4G12480 (EARLI1/pEARLI 1)
scaffold13293.g25813

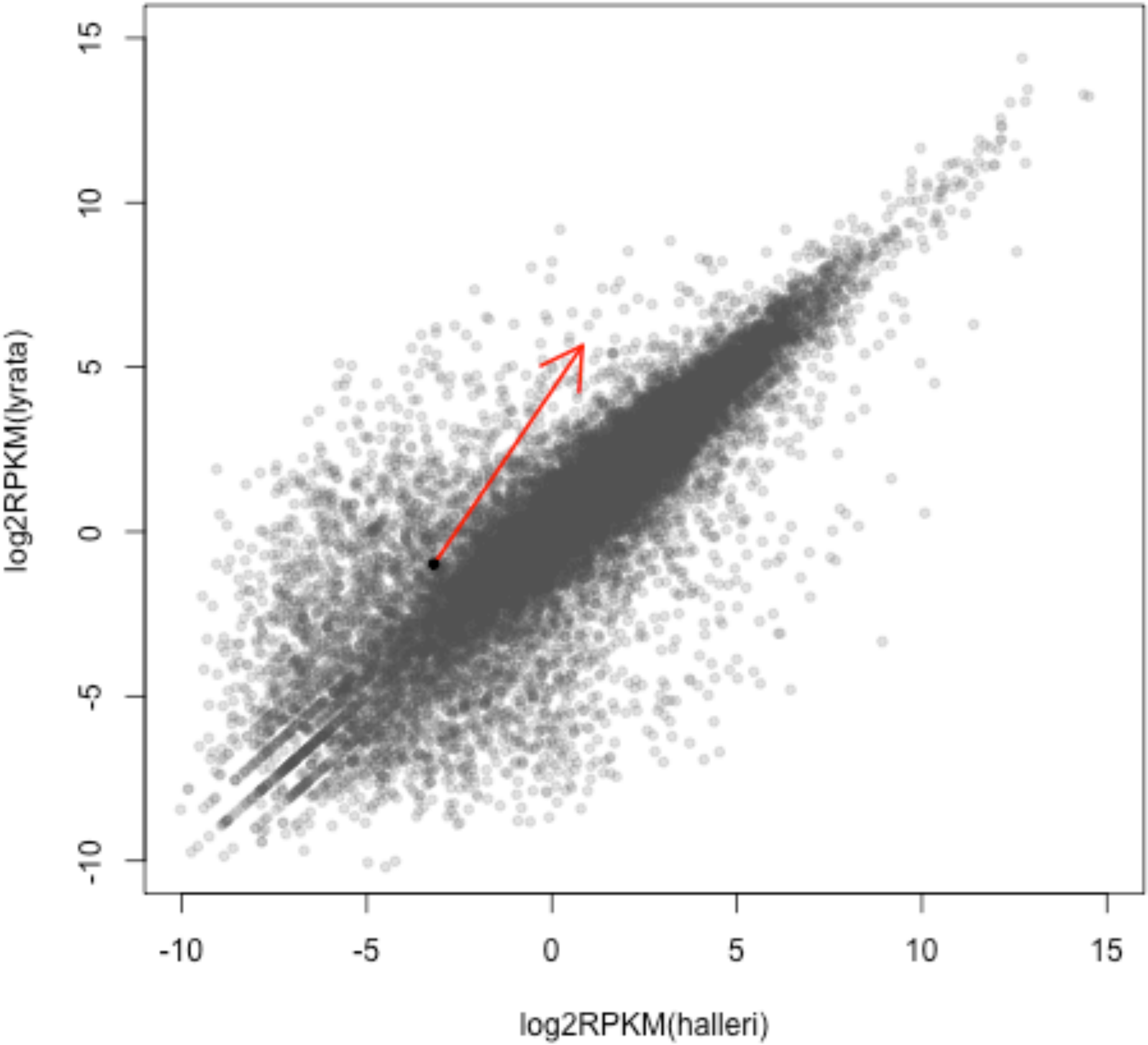

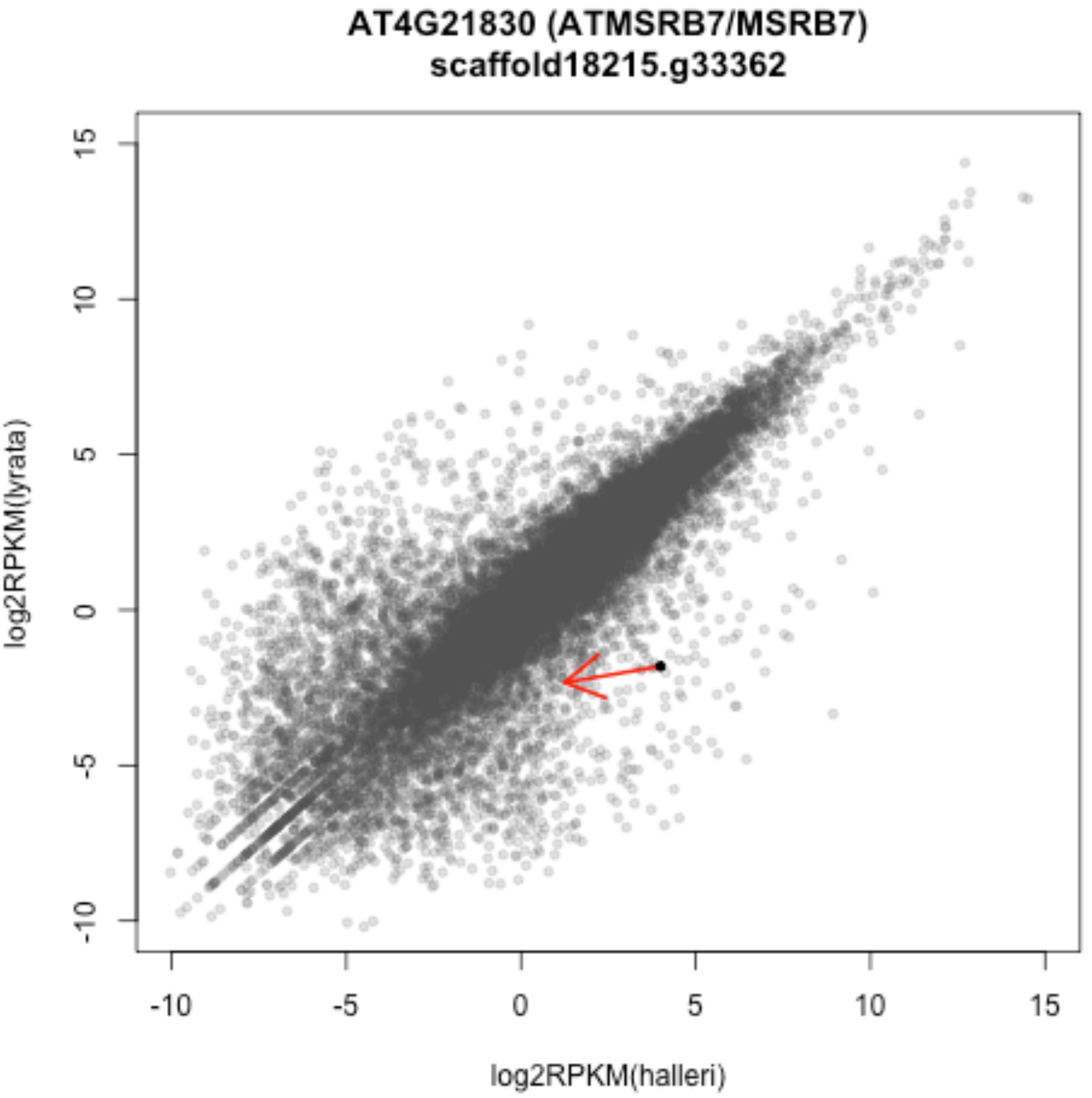
AT4G21830 (ATMSRB7/MSRB7)
scaffold18215.g33362
log2RPKM(lyrata)
log2RPKM(halleri)
-10
-5
0
5
10
15

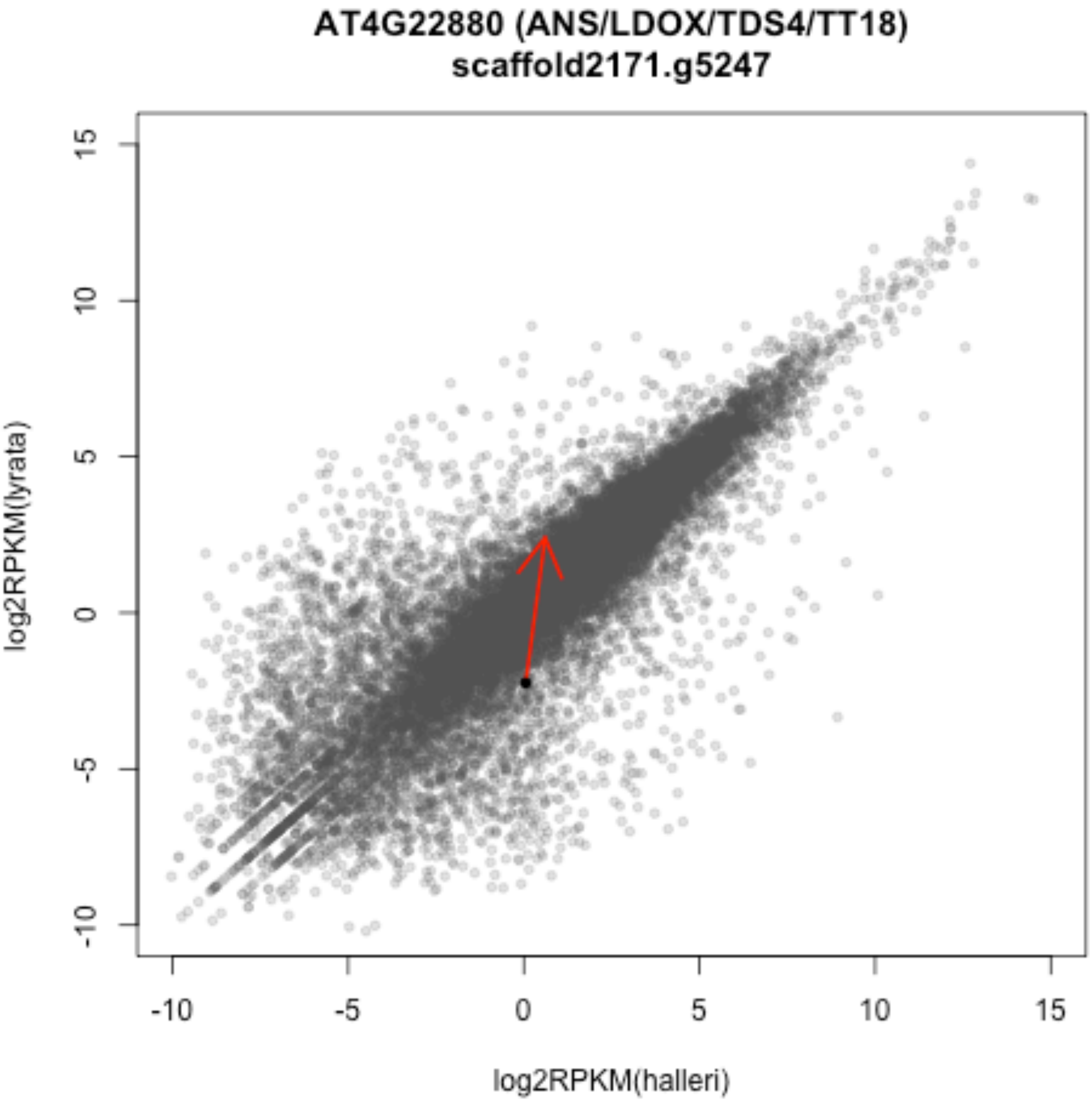
AT4G22880 (ANS/LDOX/TDS4/TT18)
scaffold2171.g5247
log2RPKM(lyrata)
log2RPKM(halleri)
-10
-5
0
5
10
15

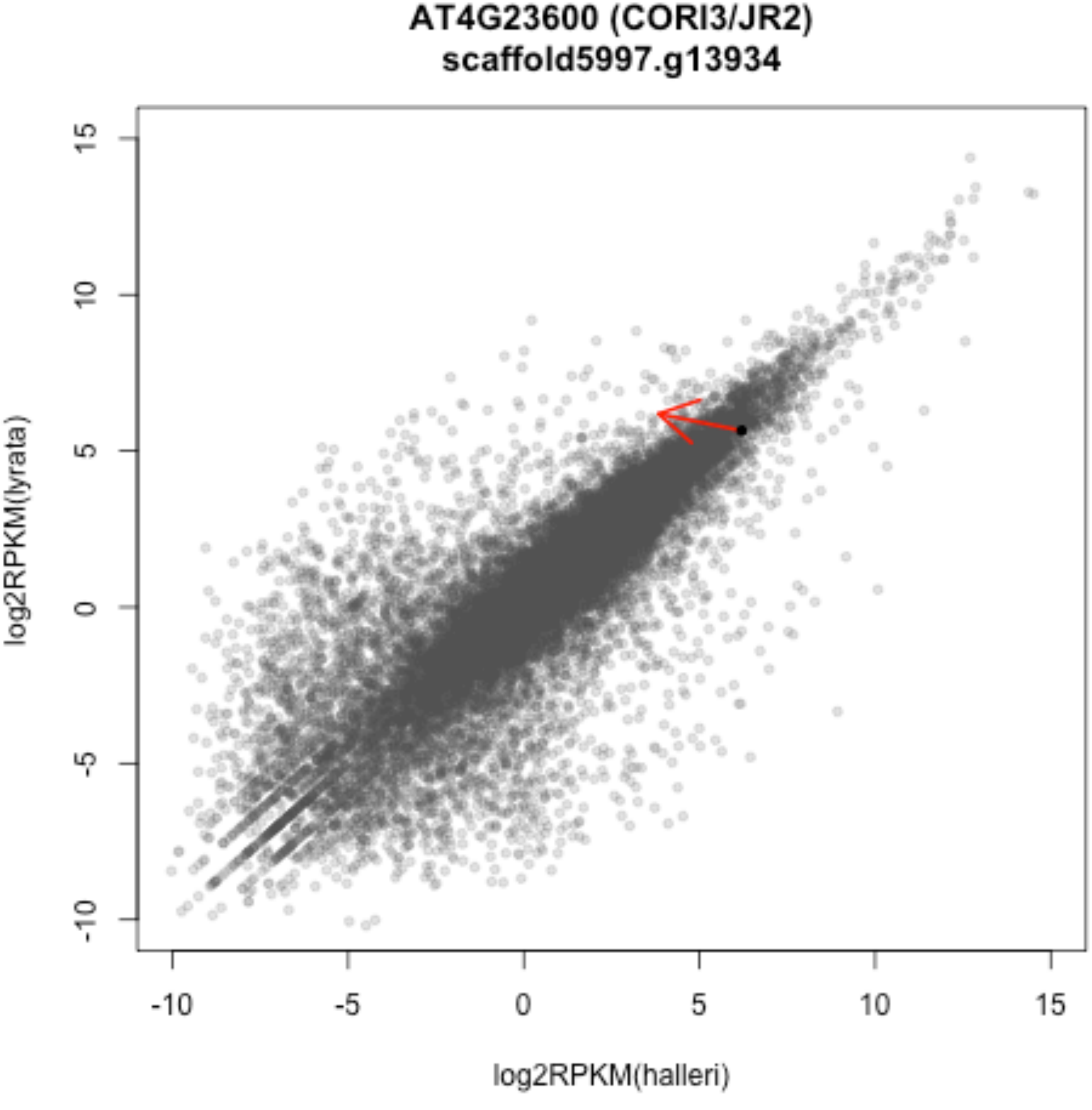
AT4G23600 (CORI3/JR2)
scaffold5997.g13934
log2RPKM(lyrata)
log2RPKM(halleri)
15
10
5
0
-5
-10
-10
-5
0
5
10
15

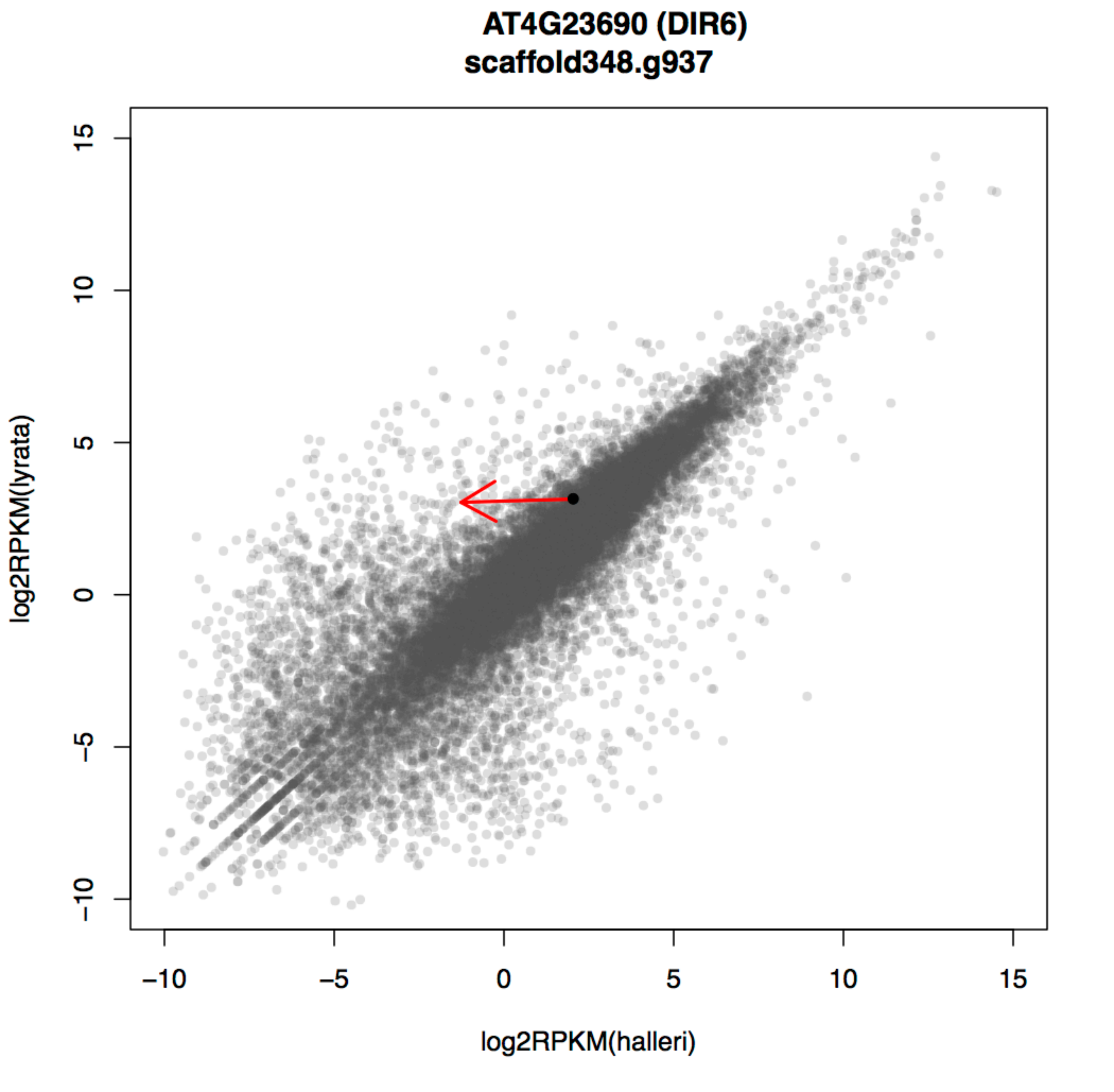
AT4G23690 (DIR6)
scaffold348.g937
log2RPKM(lyrata)
log2RPKM(halleri)
−10
−5
0
5
10
15

AT4G25200 (ATHSP23.6-MITO/HSP23.6-MITO)
scaffold14513.g27506

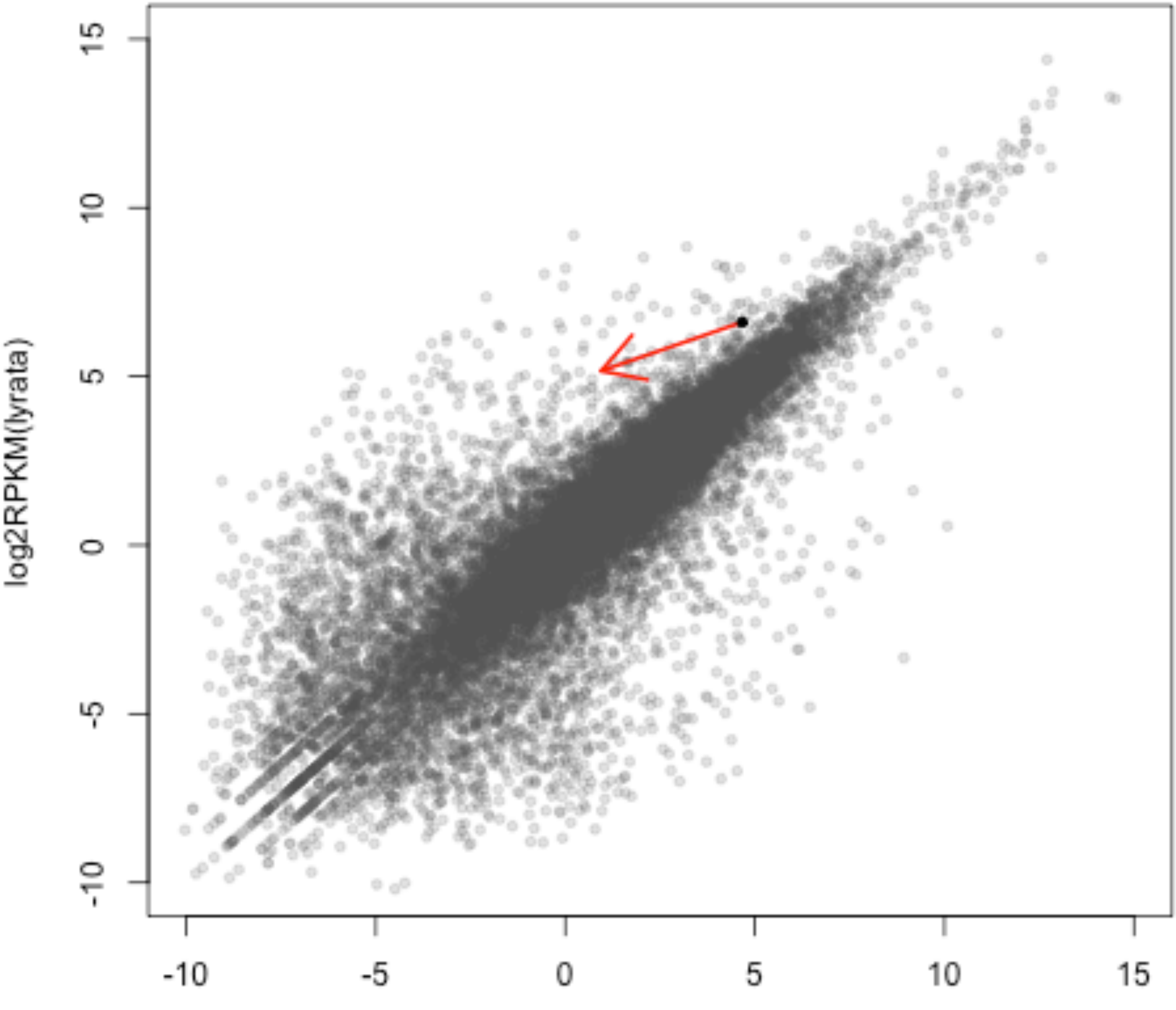

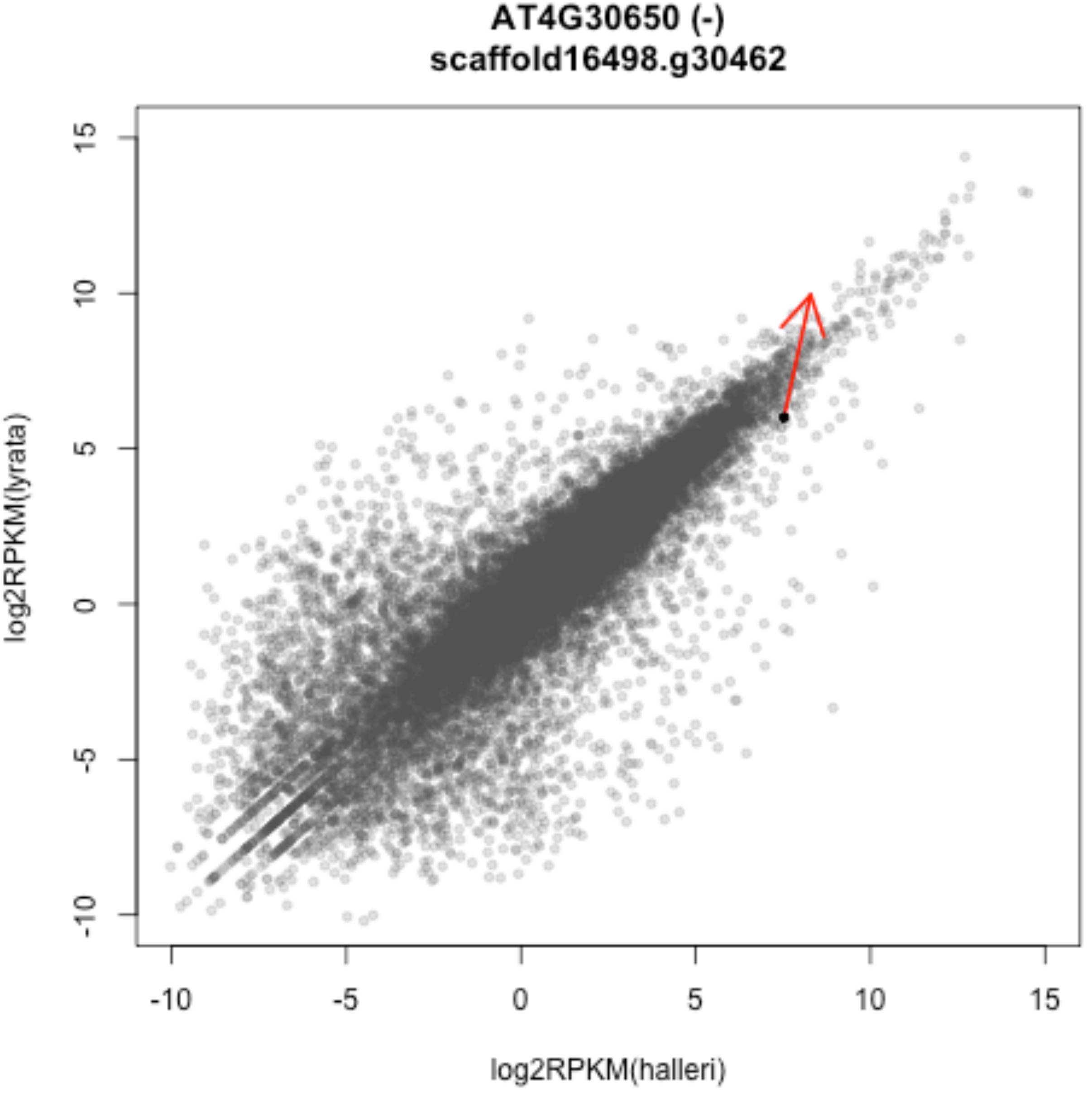
AT4G30650 (-)
scaffold16498.g30462
15
10
5
0
-5
-10
log2RPKM(lyrata)
-10
-5
0
5
10
15
log2RPKM(halleri)

AT5G07010 (ATST2A/ST2A)
scaffold2652.g6355

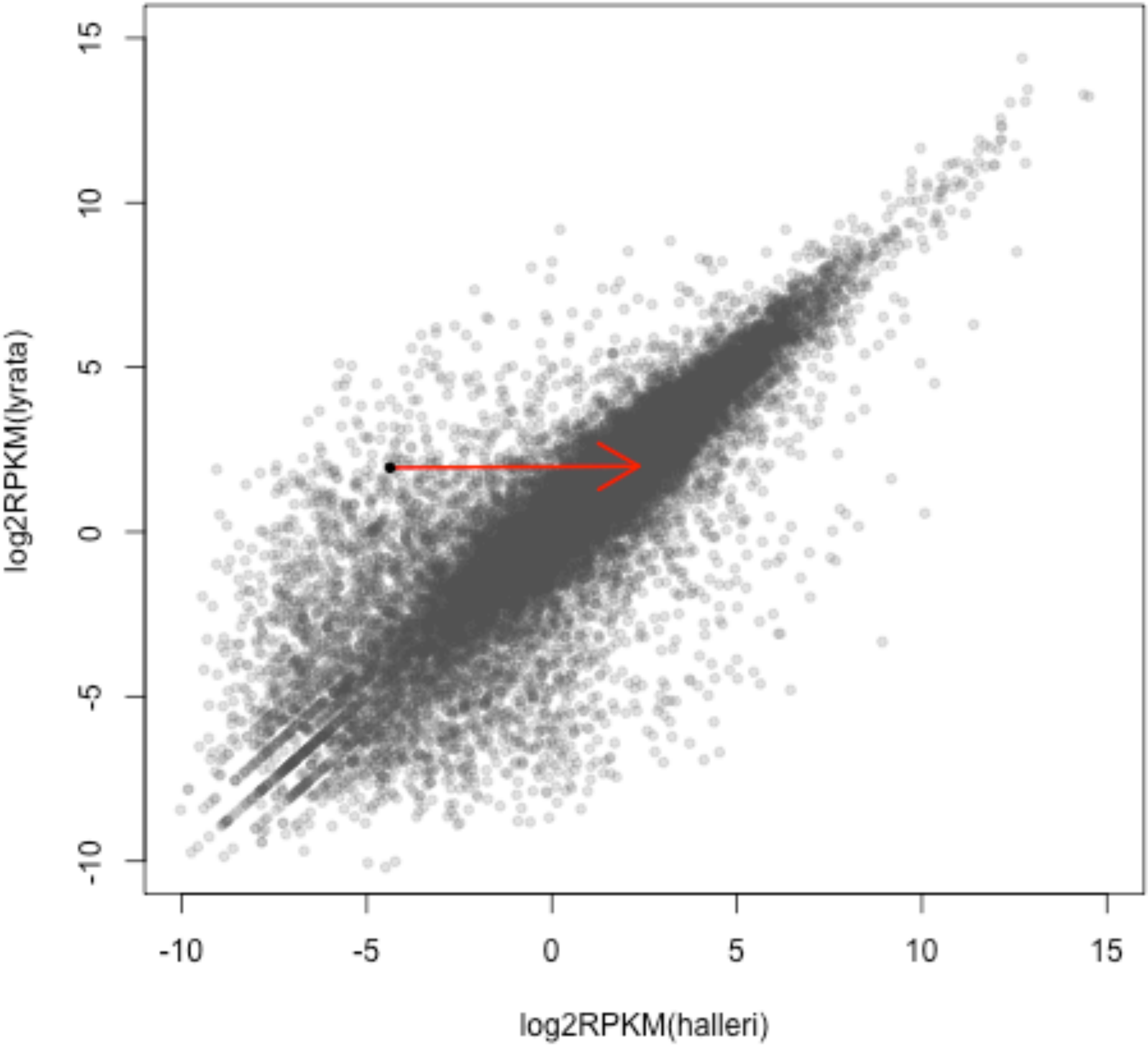

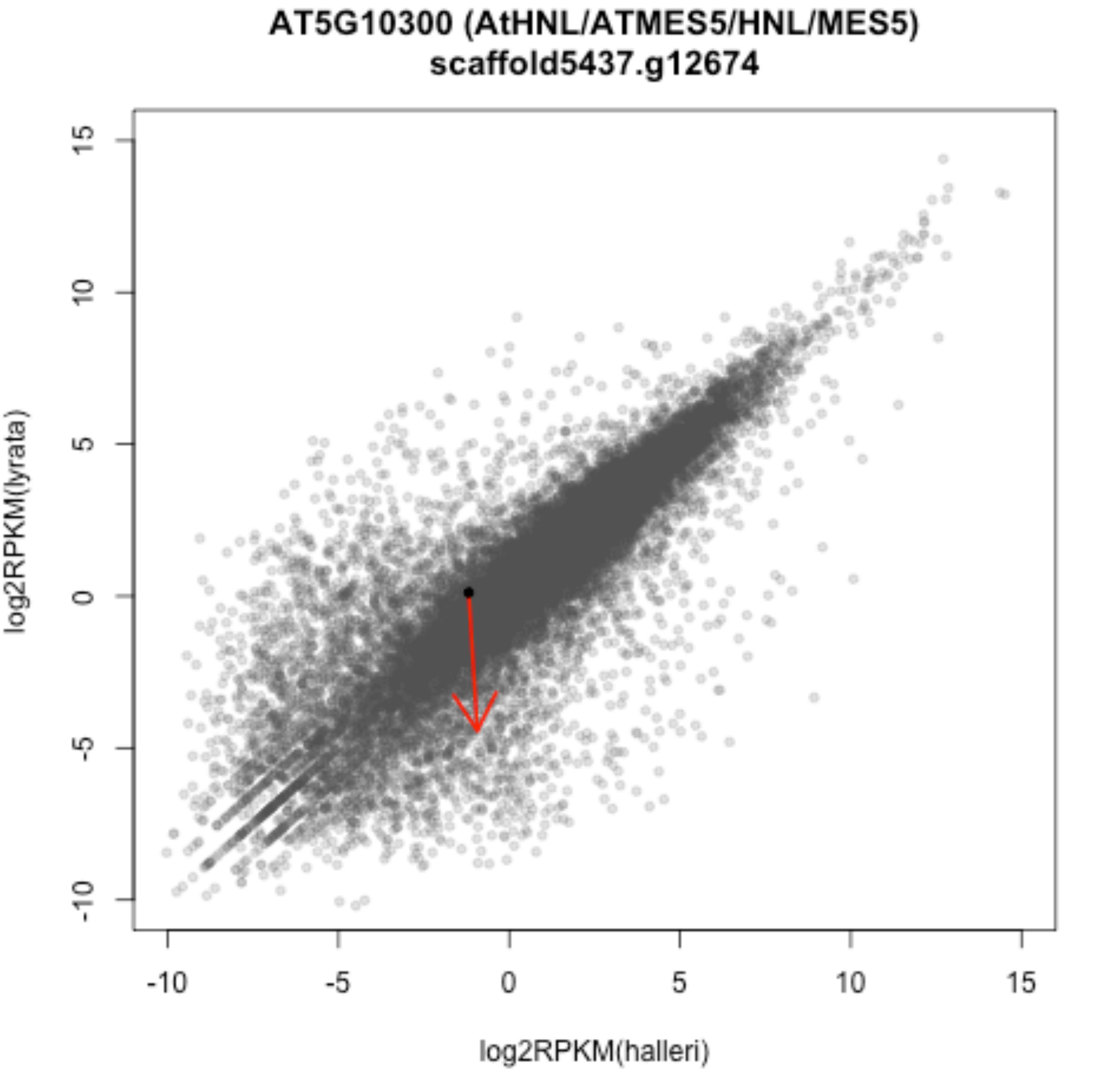
AT5G10300 (AtHNL/ATMES5/HNL/MES5)
scaffold5437.g12674
log2RPKM(lyrata)
log2RPKM(halleri)
-10
-5
0
5
10
15

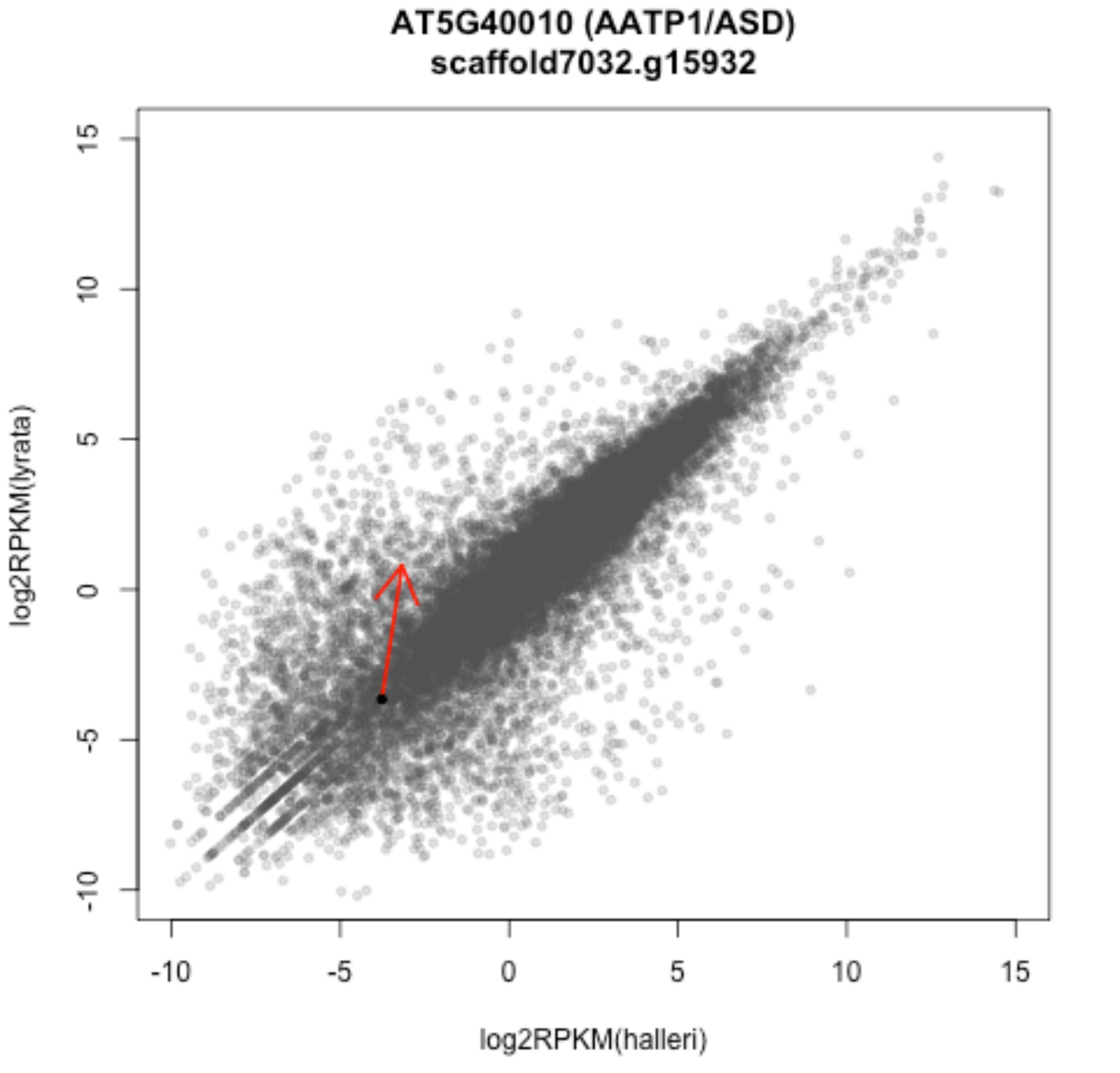
AT5G40010 (AATP1/ASD)
scaffold7032.g15932
log2RPKM(lyrata)
log2RPKM(halleri)
-10
-5
0
5
10
15

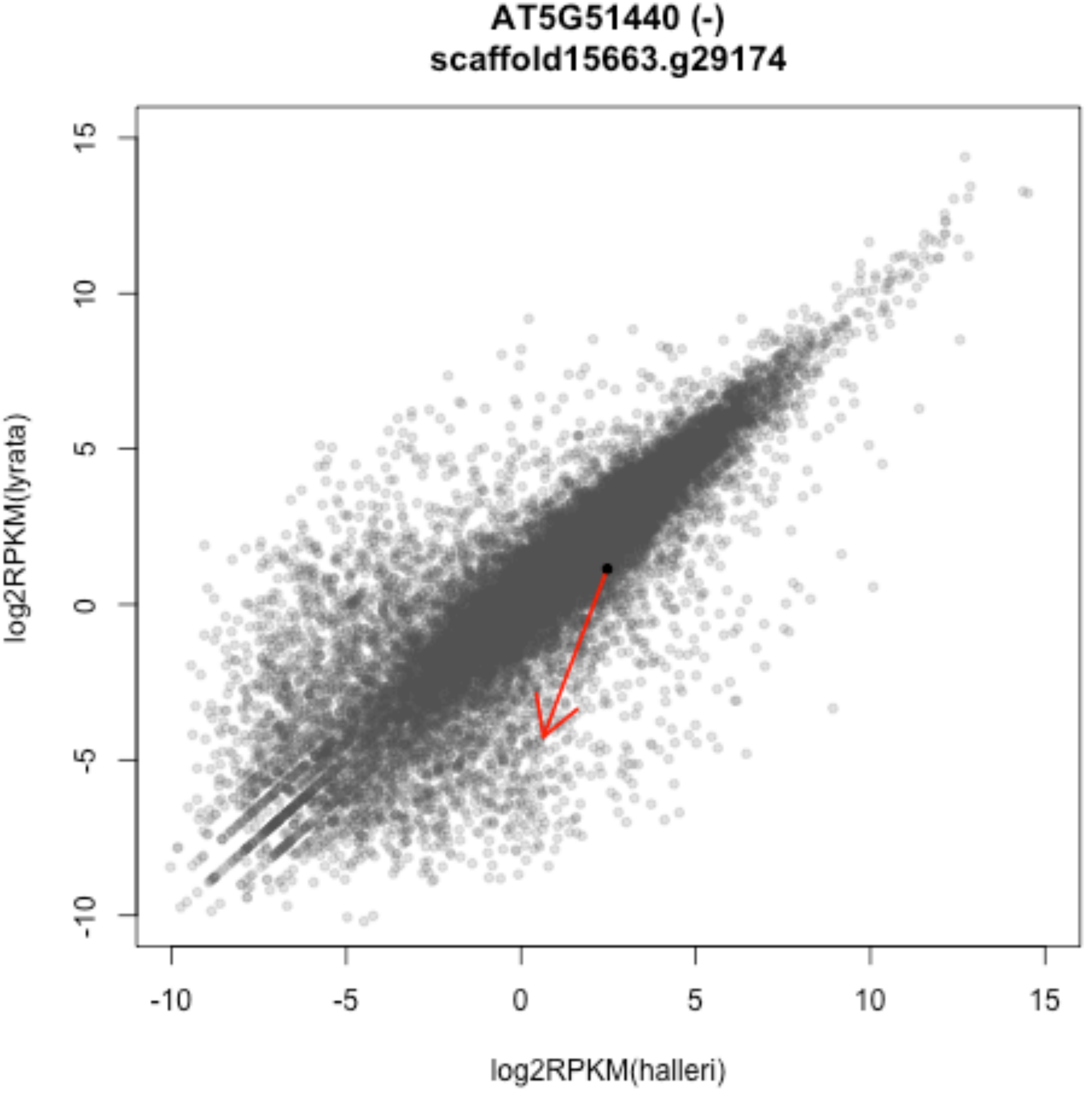

AT5G51440 (-)
scaffold15663.g29174
log2RPKM(lyrata)
log2RPKM(halleri)
-10
-5
0
5
10
15

AT5G52300 (LTI65/RD29B)
scaffold2691.g6439

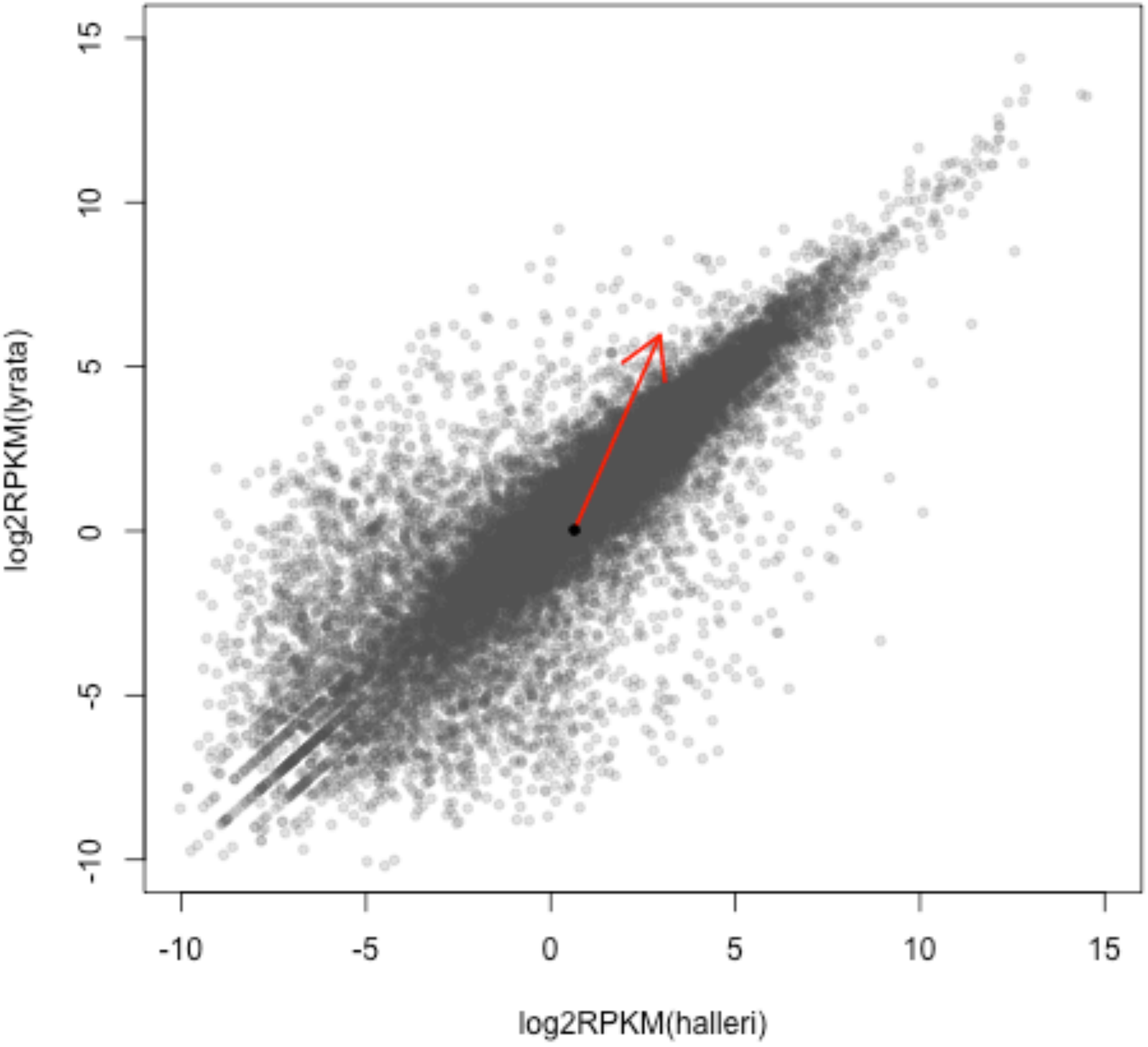

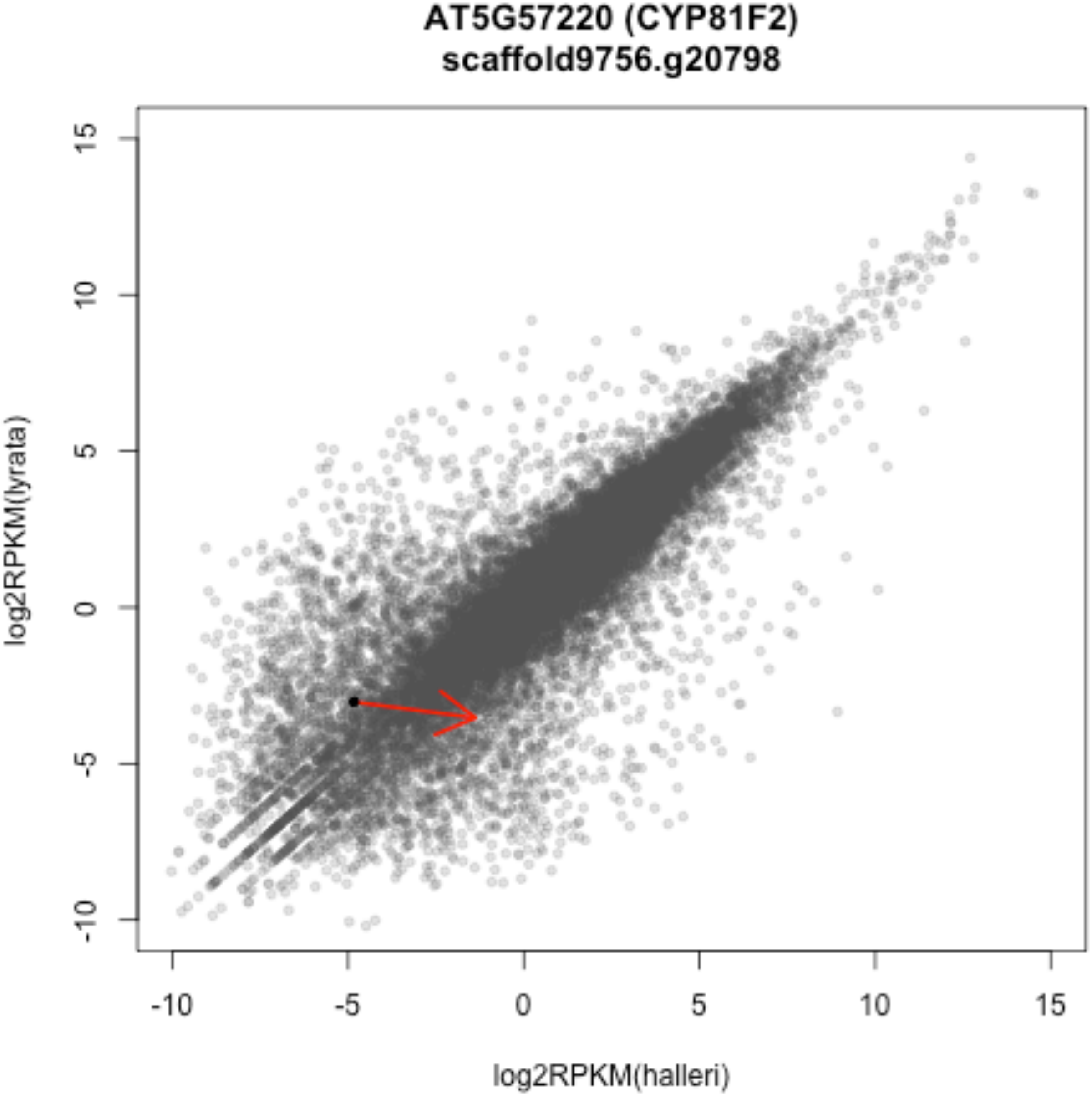
AT5G57220 (CYP81F2)
scaffold9756.g20798
log2RPKM(lyrata)
log2RPKM(halleri)
15
10
5
0
-5
-10
-10
-5
0
5
10
15

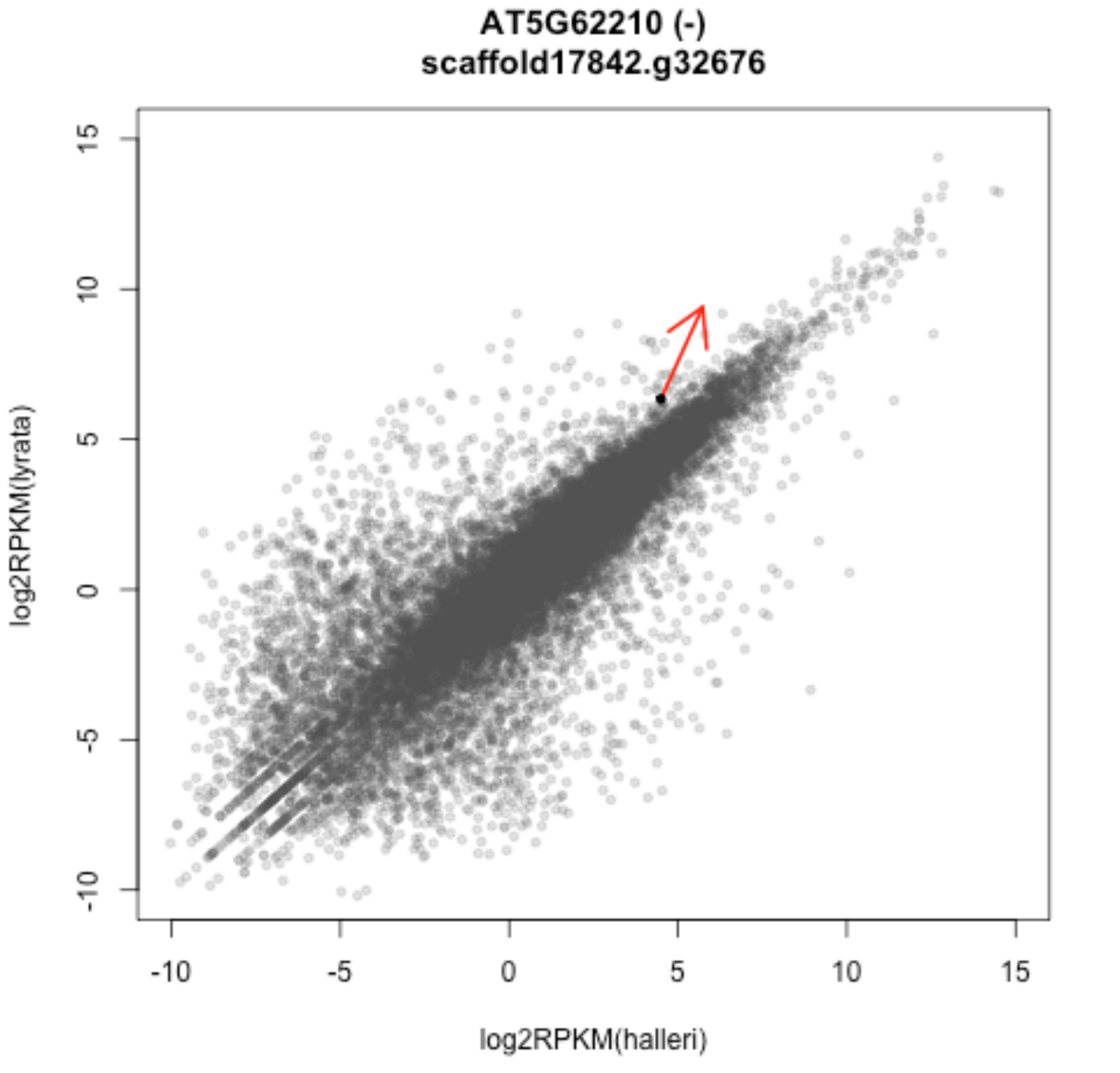
AT5G62210 (-)
scaffold17842.g32676
log2RPKM(lyrata)
log2RPKM(halleri)
-10
-5
0
5
10
15
