# Supplementary material for: Genome-wide quantification of homeolog expression ratio revealed nonstochastic gene regulation in synthetic allopolyploid Arabidopsis
Source: Nucleic Acids Res. 2014 Jan 13;42(6):e46. doi: 10.1093/nar/gkt1376 (PMC3973336; doi:10.1093/nar/gkt1376)
Supplement: Supplementary Data [file supp_gkt1376_nar-01585-met-k-2013-File007.doc]

Supplementary Table 1. Statistics of assembled *A. lyrata* and *A. halleri* genomes

|  | *A. halleri* | *A. lyrata* |
| --- | --- | --- |
| Sequenced nucleotides | 36,942,496,896 bp  (200, 500 and 800bp) | 61,445,605,532 bp  (200, 500 and 800bp) |
| Coverage (220Mbp) | 167.92x | 279.30x |
| K-mer’s K in SOAP denovo | 73 | 83 |
| # of contigs | 282,453 | 281,536 |
| N50 | 17,686 | 7,848 |
| Total length | 221.14Mbp | 202.97Mbp |
