# Supplementary material for: Genome-wide quantification of homeolog expression ratio revealed nonstochastic gene regulation in synthetic allopolyploid Arabidopsis
Source: Nucleic Acids Res. 2014 Jan 13;42(6):e46. doi: 10.1093/nar/gkt1376 (PMC3973336; doi:10.1093/nar/gkt1376)
Supplement: Supplementary Data [file supp_gkt1376_nar-01585-met-k-2013-File009.doc]

Supplementary Table 3. Statistics of expressed homeologs

|  | *A. halleri* | *A. lyrata* |
| --- | --- | --- |
| Number of homeologs | 31,749 | |
| Number of homeologs associated to A. thaliana | 20,815 (65.6%) | |
| # of expressed genes in control (*1)(ratio to total genes) | 18,683 (58.8%) | 18,988 (59.8%) |
| # of expressed genes in cold (*1) (ratio to total genes) | 18,670 (58.8%) | 18,932 (59.6%) |
| # of expressed genes in total RNA-seq samples (*1) | 18,928 (59.6%) | 19,186 (60.4%) |
|  | Expressed from both homeologs 17,745 (55.9%)  Expressed from at least one homeolog 20,369 (64.2%) | |

*1: Expressed gene are defined as RPKM > 10^-1
