# Supplementary material for: Genome-wide quantification of homeolog expression ratio revealed nonstochastic gene regulation in synthetic allopolyploid Arabidopsis
Source: Nucleic Acids Res. 2014 Jan 13;42(6):e46. doi: 10.1093/nar/gkt1376 (PMC3973336; doi:10.1093/nar/gkt1376)
Supplement: Supplementary Data [file supp_gkt1376_nar-01585-met-k-2013-File013.docx]

**Supplementary Text 1**

**Description of Supplemental Tables**

Supplementary Table 1: Statistics of assembled *A. lyrata* and *A. halleri* genomes

Supplementary Table 2: Simulated RNA-seq results. We varied the error rate of reads and the maximum number of mismatches between the read and each genome. (A) Raw count (B) Ratio of error rate

Supplementary Table 3: Statistics of expressed homeologs

Supplementary Table 4: The primers for gene fragment amplification and the targeteed SNP positions used for PyroMark analysis. The primers with * are 5'-Biotinylated. SNP position indicates the position in halleri scaffold. Two sequencing primers were designed per one gene amplification fragment as indicated in Material and Method.

Supplementary Table 5: List of homeologs whose expression ratio is significantly changed and their gene name of *A. thaliana*, closely related species.

Supplementary Table 6: GO terms statistically significant (p < 0.01 after Bonferroni correction) association with the homeologs whose expression ratio were changed in response to cold stress. The table includes GO ID, GO term, corrected p-value by Bonferroni, raw p-value, number of genes annotated to the GO term in the ratio changed homeologs, number of genes annotated to the GO term and list of the genes (TAIR IDs).

**Supplementary Figures**


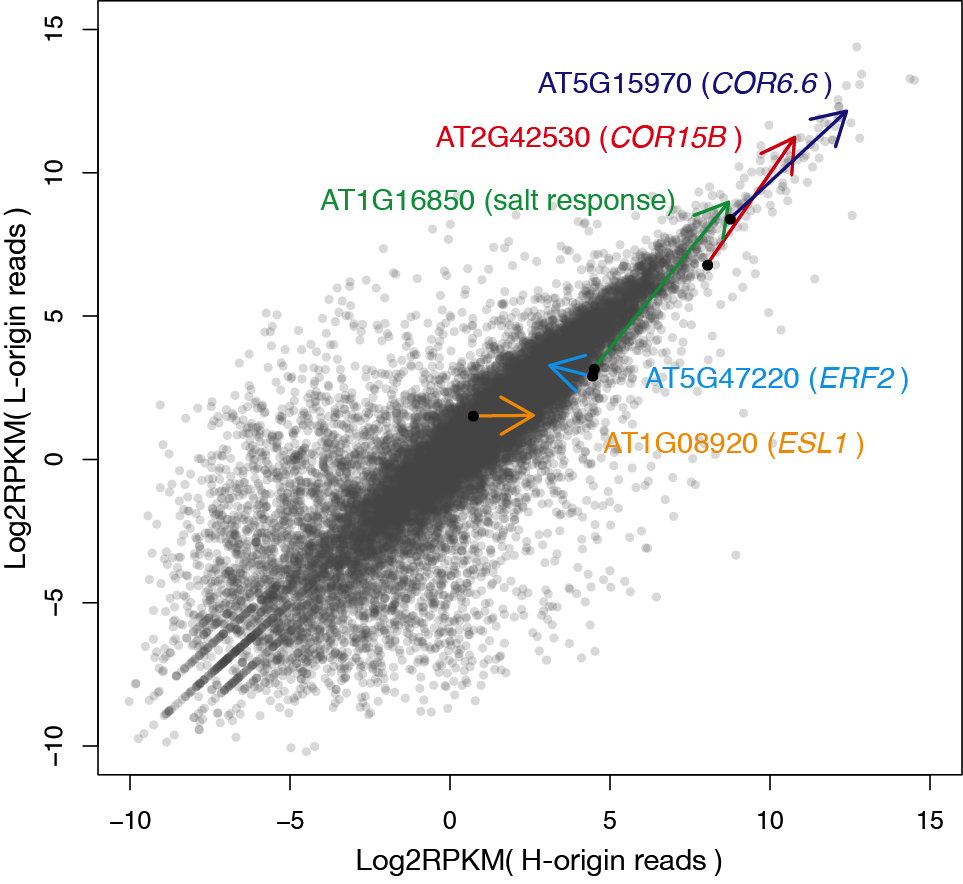


Supplementary Fig. 1: Expression level changes of homeologs used in the Pyrosequencing experiemnt. Arrows indicate the changes of the homeologs before and after cold stress treatment.


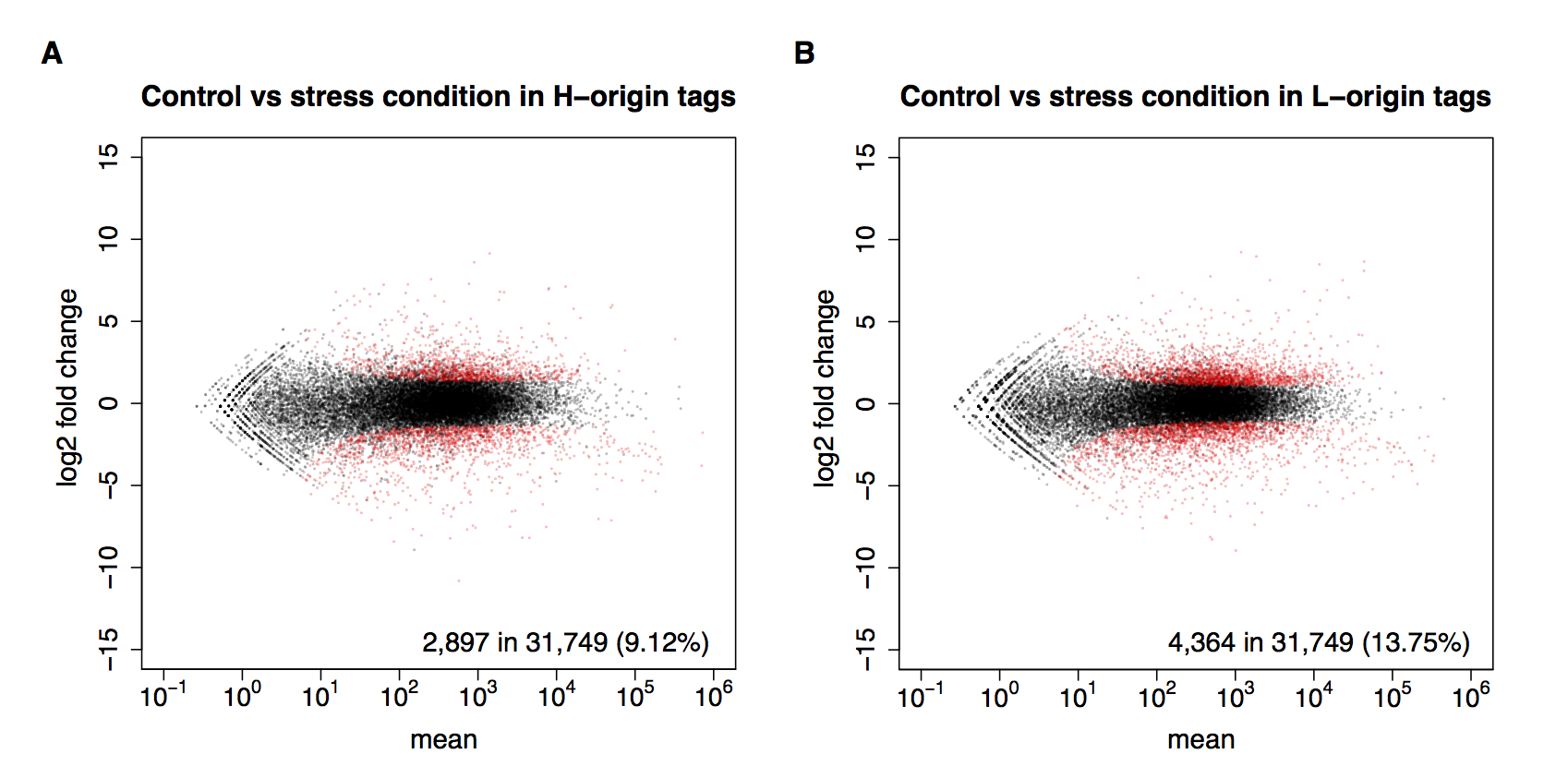


Supplementary Fig. 2. Significant expression changes between control and stress conditions. X- and Y-axis are mean of six samples over two conditions and their log2 fold changes, respectively. Red points are genes regarded as statistically significant changes in expression level. To identify the changes, DESeq were used with FDR < 0.05.


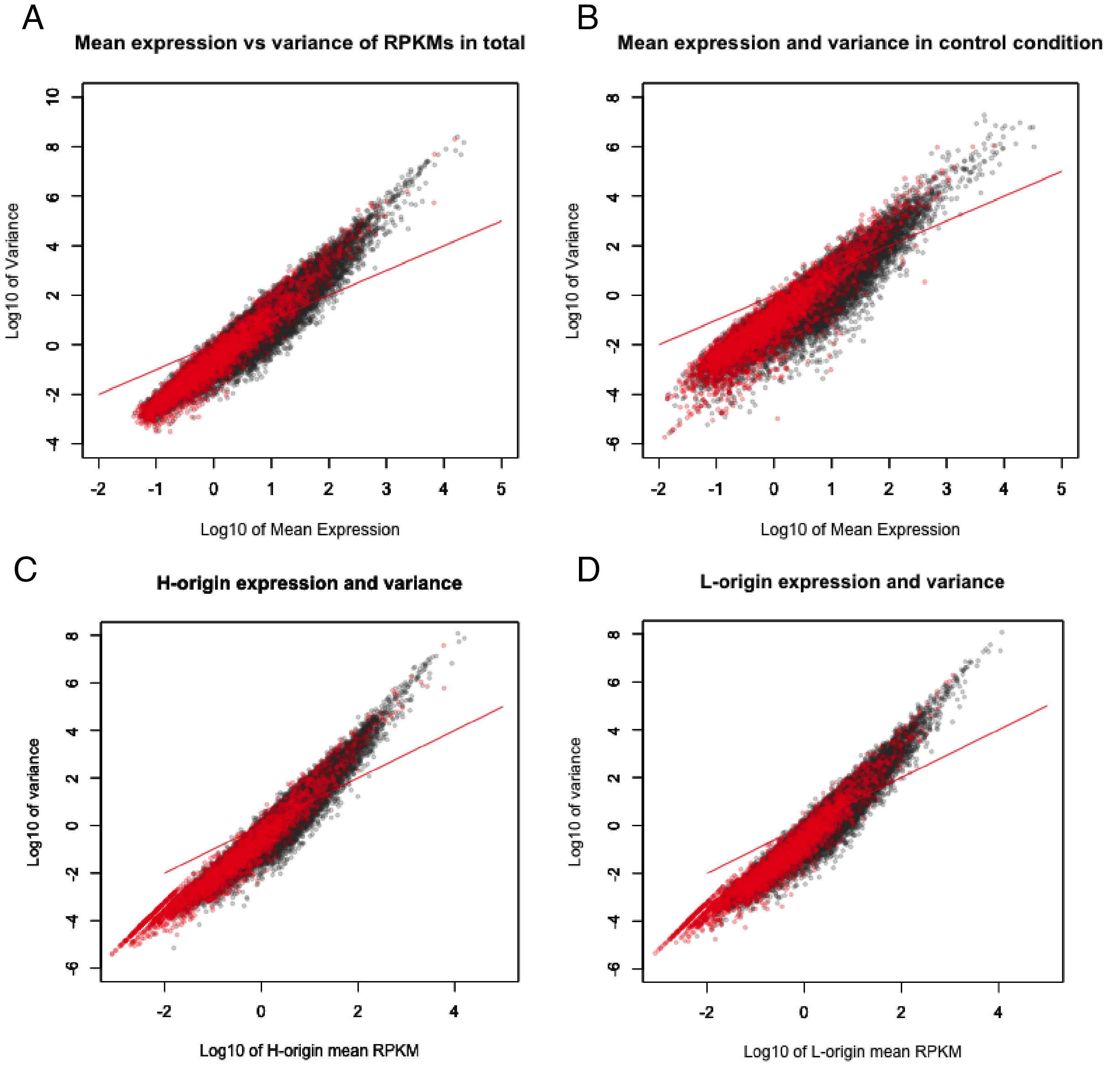
Supplementary Fig. 3. Plot of mean RPKMs of homeologs versus their variances.

(A) The relationship of means and variances of the sum of H- and L-homeolog expressions over all six samples. Each dot indicates a pair of homeologs. A black dot corresponds to a homeolog pair whose mean H-origin ratio is between 0.2 and 0.8; that is, not highly biased expression ratio. A red dot means a homeolog whose H-ratio is biased, meaning that the ratio is less than 0.2 or more than 0.8. Theoretical variance of the binomial test is shown with a red line. The variance is larger than the lines at high expression level, and hence this figure shows that the variance of the sum of homeolog expressions in *A. kamchatica* is overdispersed. Furthermore, the figure indicates that there is no clear difference between homeologs of low and high H-ratios.

(B) The relationship of means and variances only in control condition. Black dots, red dots and a red line are low H-ratio homeologs, high H-ratio homeologs and theoretically expected variance, respectively. This view shows the variance among biological replicates of three samples. We confirmed the existence of the overdispersion even only in control condition, and that the overdipersion did not come from the difference between control and cold stress conditions.

(C)(D) The relationships of means and variances over six samples only in H-origin and L-origin expressions, respectively. They have no clear difference, and hence we confirmed that the overdispersion exists even if we focused on homeologs derived from a single parent.

Supplementary Fig. 4. (All figures are in Supplementary Text 2)

Expression level changes of homeologs related to stress environment. This pdf include 49 figures, each of which indicates a change of single homeolog in response to cold stress. In each figure, X-axis and Y-axis are RPKM of H- and L-origin expressions, respectively. Gray dots represent expression levels of entire homeologs. A black dot in each figure indicates the expression level of the gene denoted at the top of each figure, and the red arrow from the point indicates the expression change after the cold stress. The tip of the arrow represents the expression level of the gene after the cold stress.


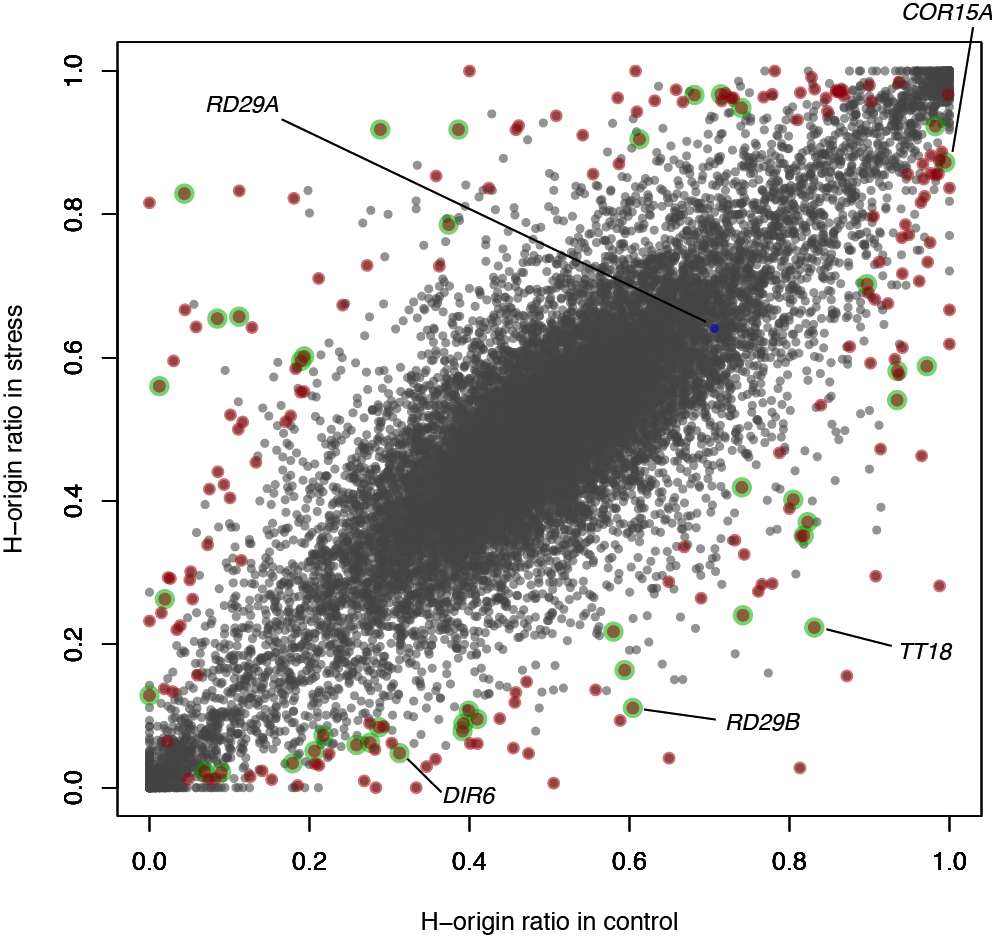


Supplementary Fig. 5. Overview of ratio changes about stress response genes. Red points are homeologs with statistical significance change of ratio, which are identical to Figure 4C. Among those homeologs, we further highlighted the genes included in the GO term “responses to stress” (GO: 0006950) with green.
